# Supplementary material for: The genomic impact of population connectivity and decline in Africa’s elephants
Source: Nat Commun. 2026 Apr 16;17:3223. doi: 10.1038/s41467-026-71262-w (PMC13086863; doi:10.1038/s41467-026-71262-w)
Supplement: Supplementary file 1 — Supplementary information [file 41467_2026_71262_MOESM1_ESM.pdf]

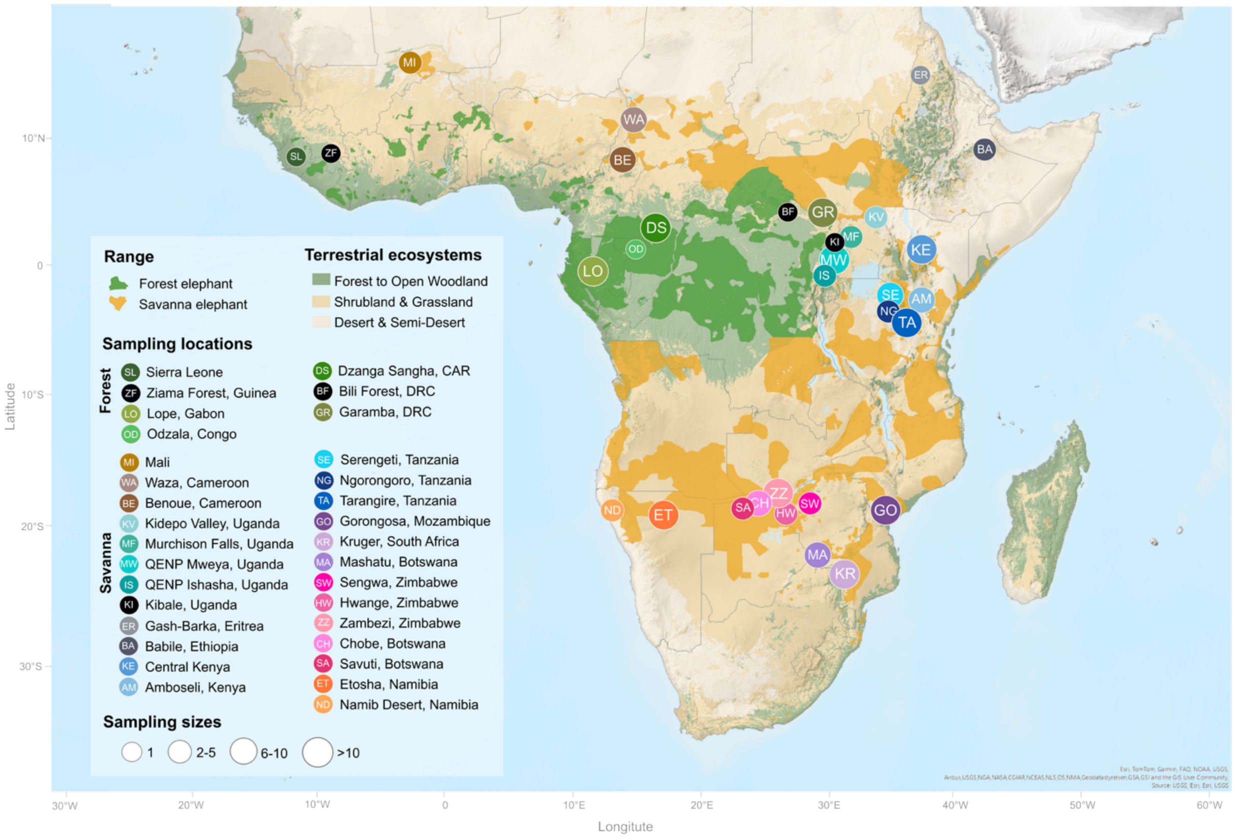

Fig. S1. An extended version of Fig. 1 showing a map of sub-Saharan Africa with the locations of origin of the samples analysed in this study plotted against the distribution of terrestrial ecosystems[1] and the current species ranges, including possible ranges, according to the IUCN Red List of Threatened Species[2,3]. Compared to Fig. 1, this version includes sampling sizes (size of circles) and includes locations for which samples failed quality control (in black). The map was created using ArcGIS® Online using basemap and spatial data[55–58] sourced by © Esri and its data providers. The map was edited in InkScape v1.2.1.

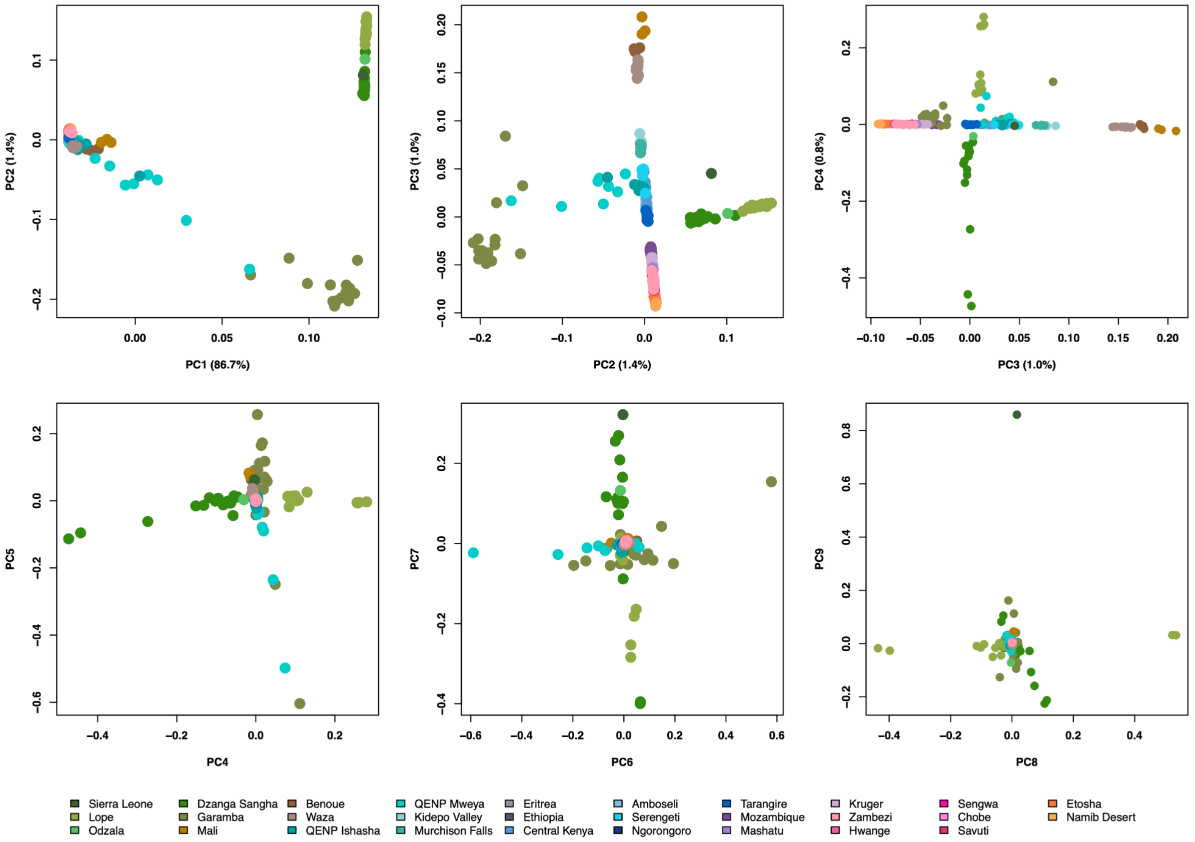

Fig. S2. Population structure of both species of African elephants based on the imputed dataset of 232 savanna and forest elephants. Principal component analysis was performed in PLINK v1.9[8] and shows principal components (PCs) 1 to 9.

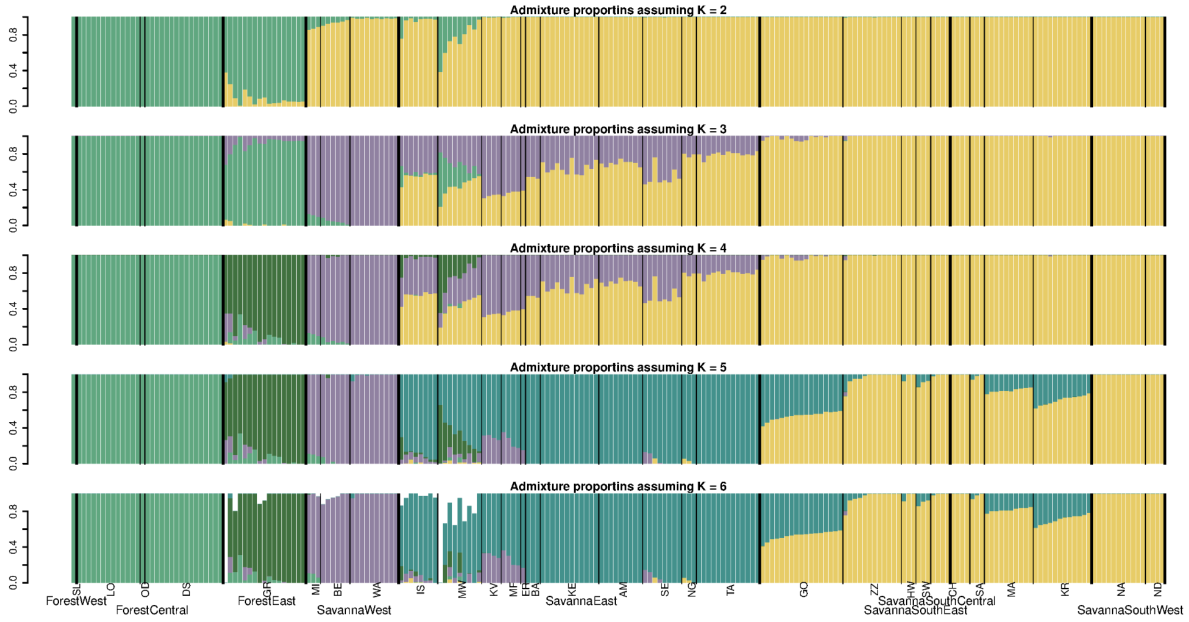

Fig. S3. Admixture analysis including both species of African elephants performed in ADMIXTURE v1.3.0[9] on the imputed dataset of 232 genomes. Analysis converged up to $K = 6$. At $K = 6$, a cluster that is challenging to explain biologically was inferred and as it does not have a clear proxy among our sampled locations, it is coloured as white.

Fig. S4. a)

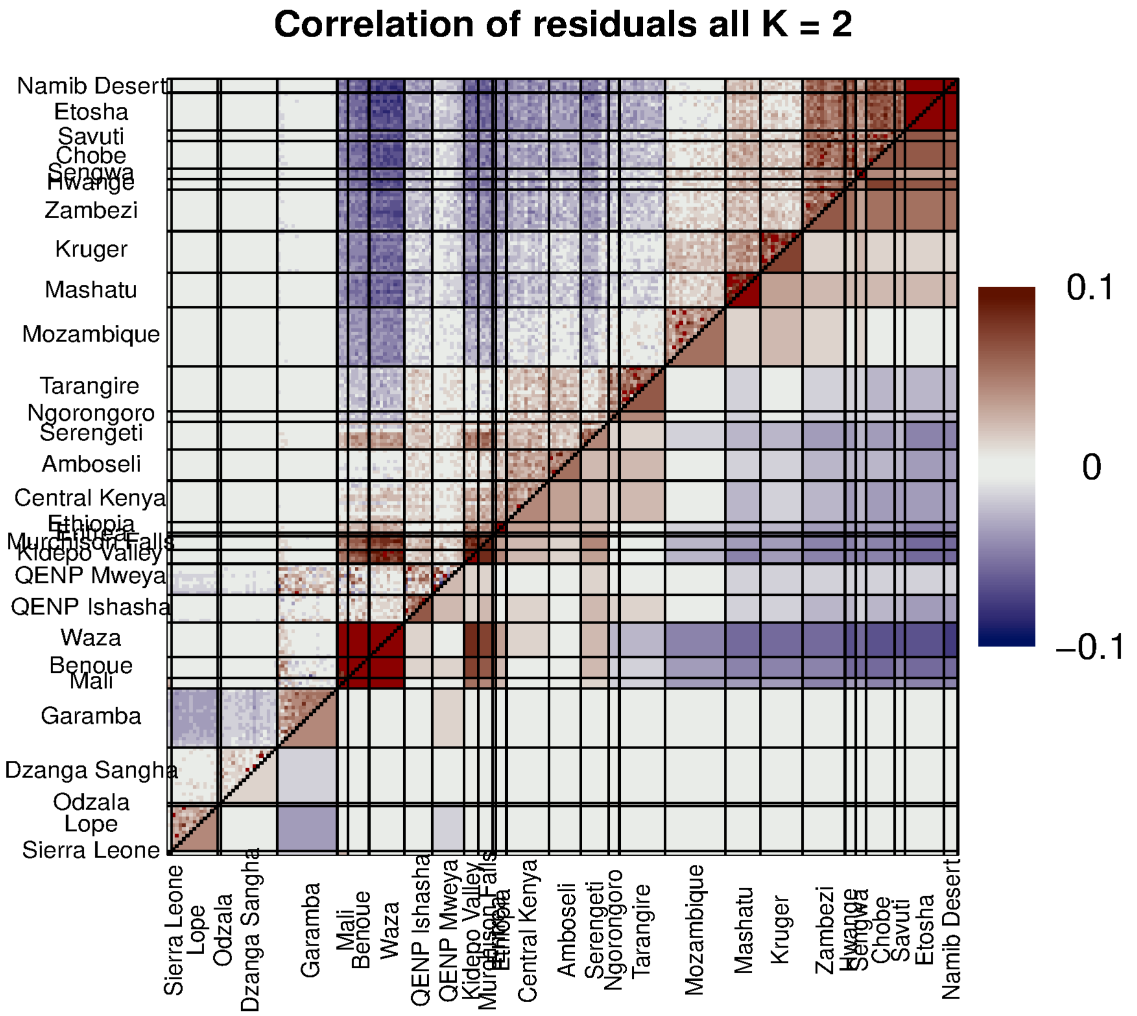

Fig. S4. Evaluation of the model fit of the inferred admixture proportions for all elephants of the maximum likelihood run for each converged K performed in evalAdmix v0.962[10]. a) K=2; b) K=3; c) K=4; d) K=5; e) K=6. Due to small sample sizes in some groups, the titles of some rows and columns are overlapping–the populations are ordered horizontally from top to bottom and vertically from right to left in the following order: “Namib Desert, Etosha, Savuti, Chobe, Sengwa, Hwange, Zambezi, Kruger, Mashatu, Mozambique, Tarangire, Ngorongoro, Serengeti, Amboseli, Central Kenya, Ethiopia, Eritrea, Murchison Falls, Kidepo Valley, QENP Mweya, QENP Ishasha, Waza, Benoue, Mali, Garamba, Dzanga Sangha, Odzala, Lope Sierra Leone”.

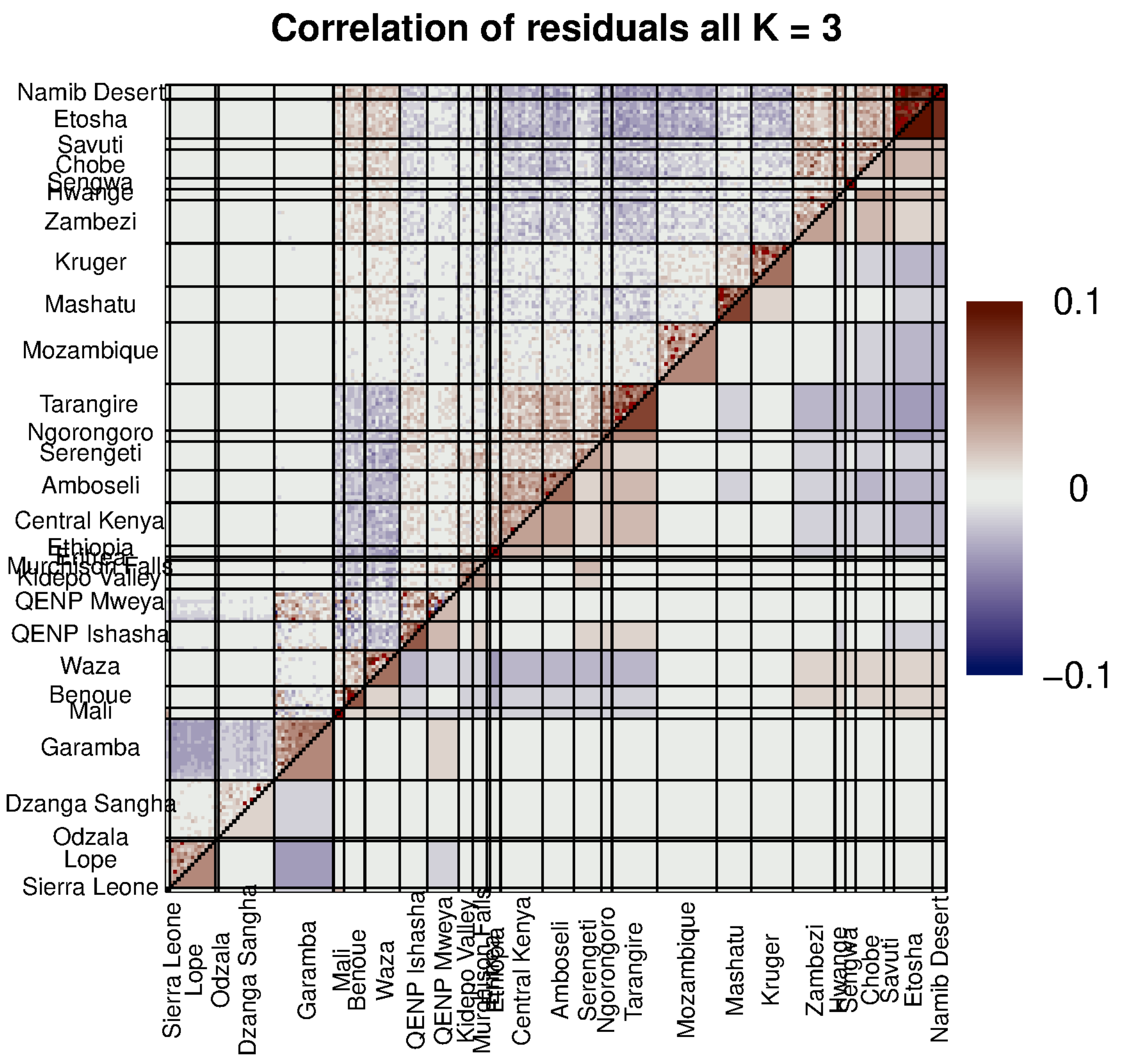
Correlation of residuals all K = 3
Namib Desert
Etosha
Savuti
Chobe
Sengwa
Hwange
Zambezi
Kruger
Mashatu
Mozambique
Tarangire
Ngorongoro
Serengeti
Amboseli
Central Kenya
Ethiopia
Eritrea
Murchison Falls
Kidepo Valley
QENP Mweya
QENP Ishasha
Waza
Benoue
Mali
Garamba
Dzanga Sangha
Odzala
Lope
Sierra Leone
0.1
0
−0.1

Fig. S4.b)

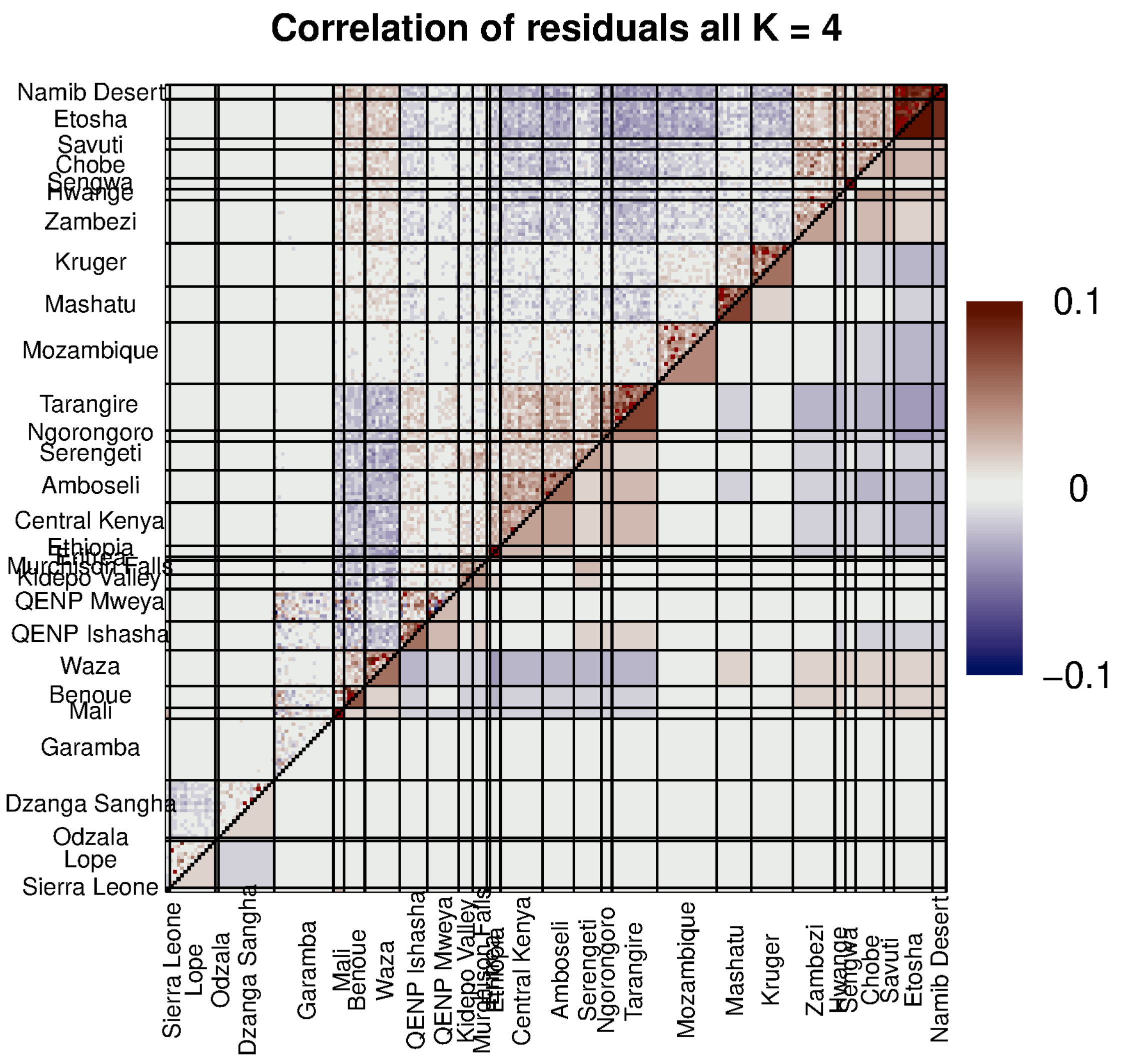

Correlation of residuals all K = 4
Namib Desert
Etosha
Savuti
Chobe
Sengwa
Hwange
Zambezi
Kruger
Mashatu
Mozambique
Tarangire
Ngorongoro
Serengeti
Amboseli
Central Kenya
Ethiopia
Eritrea
Murchison Falls
Kidepo Valley
QENP Mweya
QENP Ishasha
Waza
Benoue
Mali
Garamba
Dzanga Sangha
Odzala
Lope
Sierra Leone
0.1
0
−0.1

Fig. S4.c)

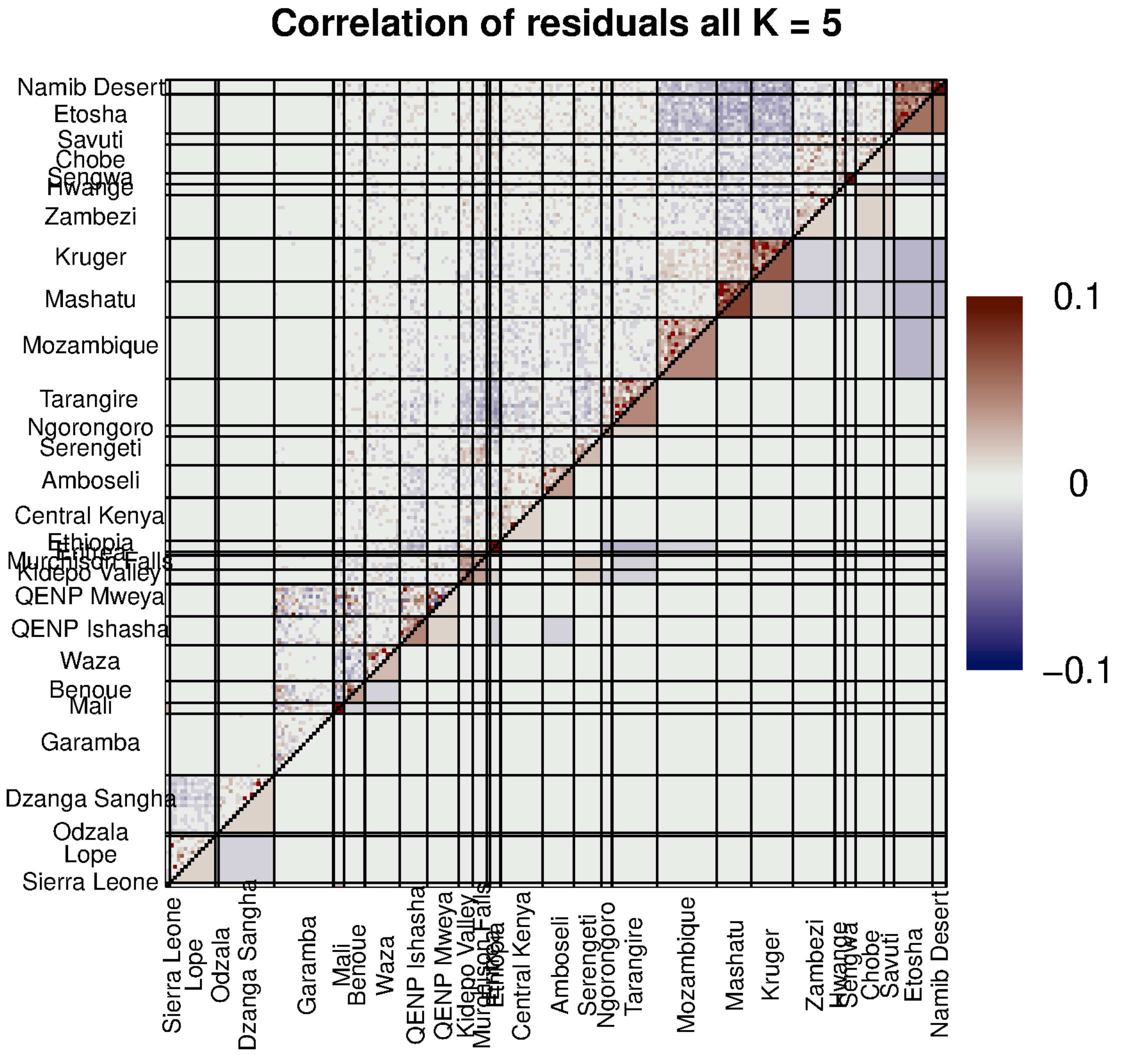
Correlation of residuals all K = 5
Namib Desert
Etosha
Savuti
Chobe
Sengwa
Hwange
Zambezi
Kruger
Mashatu
Mozambique
Tarangire
Ngorongoro
Serengeti
Amboseli
Central Kenya
Ethiopia
Eritrea
Murchison Falls
Kidepo Valley
QENP Mweya
QENP Ishasha
Waza
Benoue
Mali
Garamba
Dzanga Sangha
Odzala
Lope
Sierra Leone
0.1
0
−0.1

Fig. S4.d)

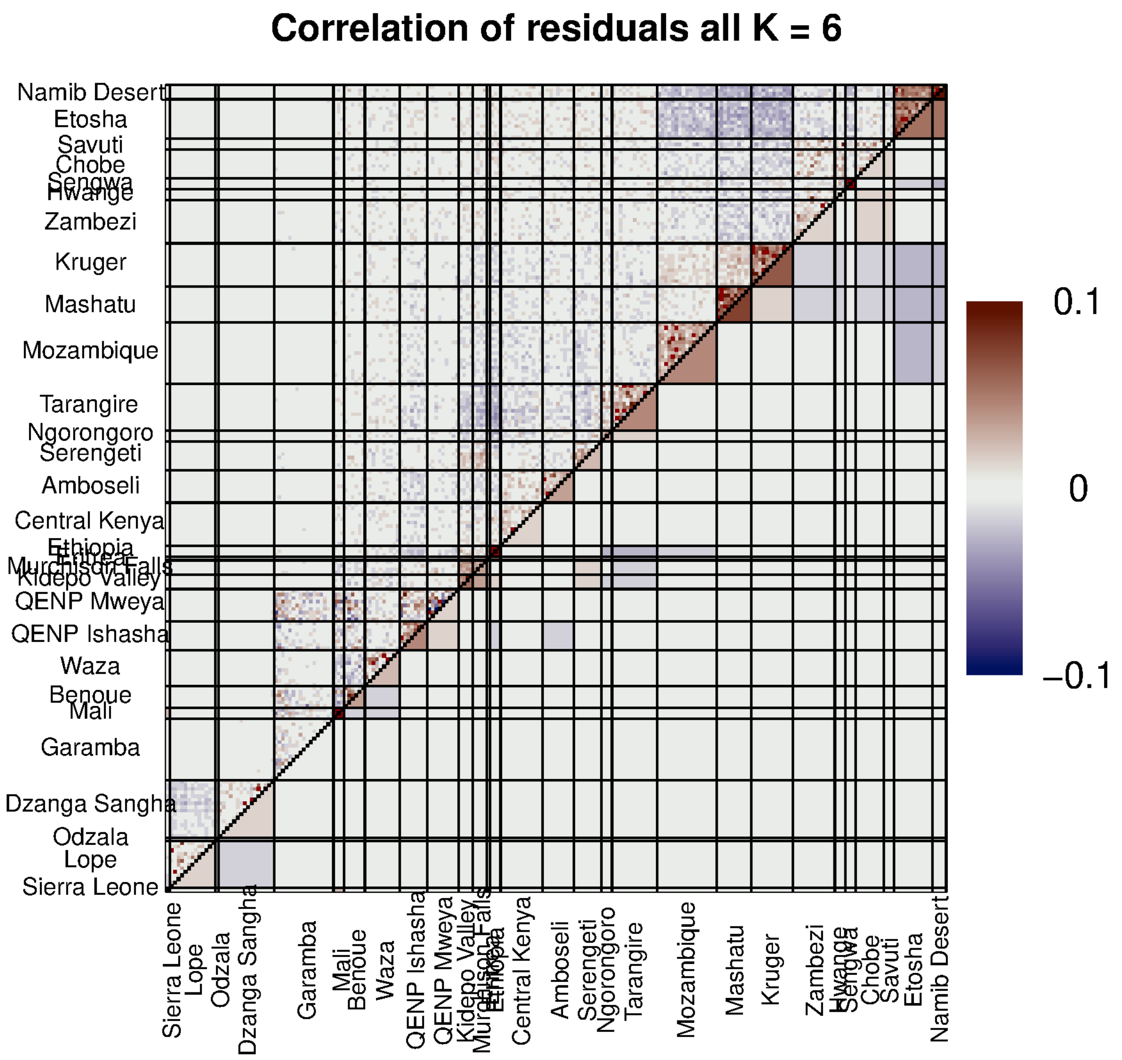
Correlation of residuals all K = 6
Namib Desert
Etosha
Savuti
Chobe
Sengwa
Hwange
Zambezi
Kruger
Mashatu
Mozambique
Tarangire
Ngorongoro
Serengeti
Amboseli
Central Kenya
Ethiopia
Eritrea
Murchison Falls
Kidepo Valley
QENP Mweya
QENP Ishasha
Waza
Benoue
Mali
Garamba
Dzanga Sangha
Odzala
Lope
Sierra Leone
0.1
0
−0.1

Fig. S4. e)

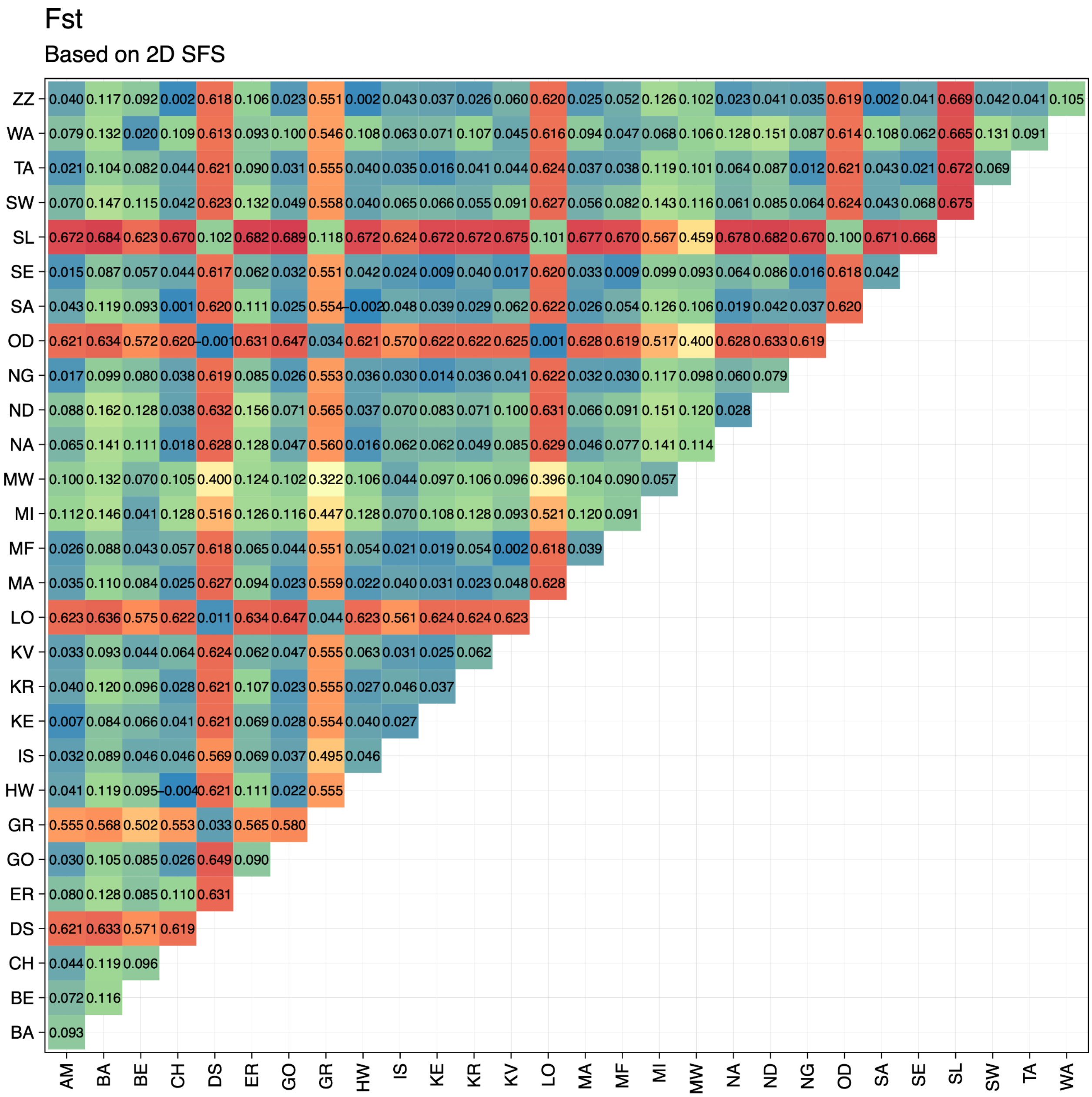

Fig. S5. F_{ST} estimated using the corrected Hudson's estimator[11] based on 2D site frequency spectra generated as part of the genotype calling pipeline[12] available at https://github.com/popgenDK/seqAfrica_wildebeest/tree/main/GT_calling. First-degree relatives were excluded.

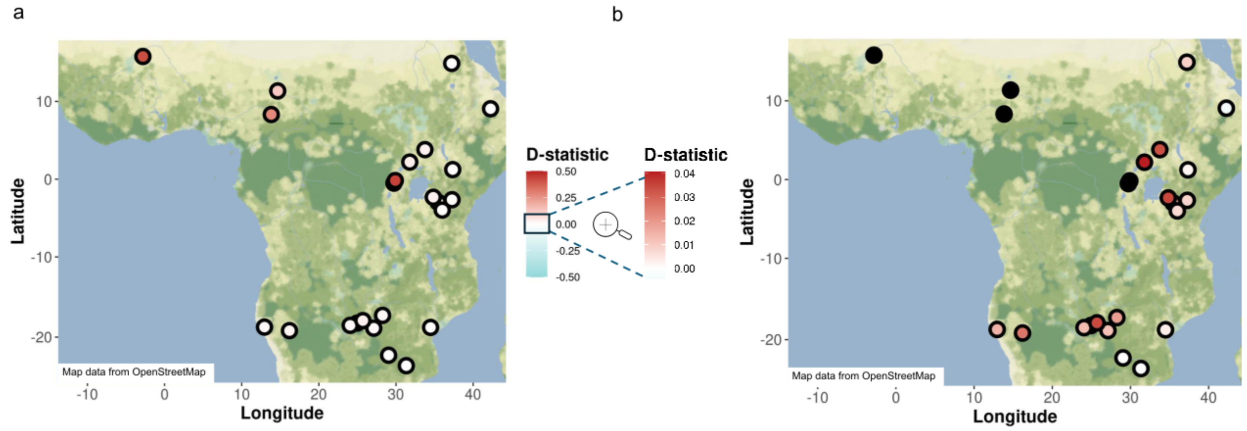

Fig. S6. *D*-statistics[13,14] showing patterns of forest elephant allele sharing among the savanna elephant populations. All D-statistics were summarized by fixing a relatively non-admixed savanna population (Kruger) as H1, a relatively non-admixed forest population (Lope) as H3, varying each population as H2, and finally the average D-statistics value across individuals was used to represent each population. a) Higher proportions of forest ancestry are evident in west-central Africa and QENP. b) Enhanced color saturation shows a weaker signal of forest ancestry in populations outside of the recognized hybrid zones. All D-statistics with individual samples are available in Fig. S7. The map was generated in R v4.2.2[15] using R package ggplot2[16] and the Stamen Terrain basemap accessed via Stadia Maps. Map tiles: © Stadia Maps © Stamen Design © OpenMapTiles © OpenStreetMap contributors. Map data: © OpenStreetMap contributors (ODbL).

a)

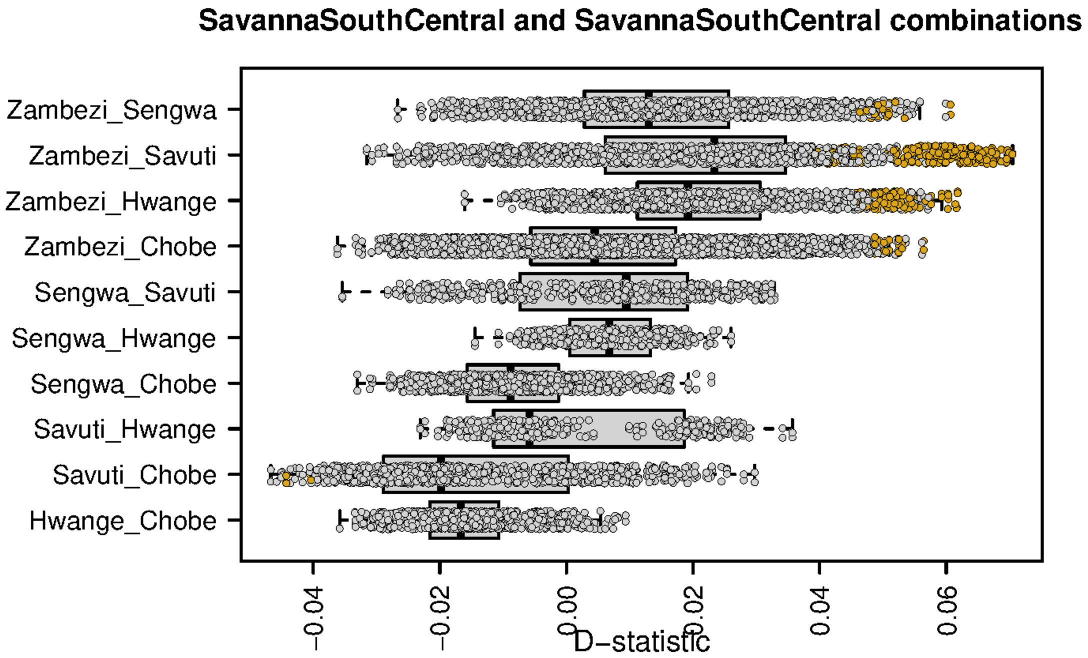

Fig. S7. Analysis of gene flow between species using *D*-statistics[13,14] on the dataset mapped to the Asian elephant. We tested whether any savanna elephant population showed an excess of allele sharing with the forest elephant by placing forest elephants (all forest elephant samples except the admixed Garamba population) as H3 and all combinations of savanna elephants in H1 and H2. Please observe that the labels are switched, thus, for example, the first example shows significant excess of allele sharing in Zambezi compared to Sengwa. a) savanna south-central vs savanna south-central; b) savanna south-central vs savanna south-west; c) savanna east vs savanna south-east; d) savanna east vs savanna south-west; e) savanna south-east vs savanna south-central; f) savanna south-east vs savanna south-east; g) savanna south-east vs savanna south-west; h) savanna south-west vs savanna south-west; i) savanna west vs savanna east; j) savanna west vs savanna south-central; k) savanna west vs savanna south-east; l) savanna west vs savanna south-west; m) savanna west vs savanna west; n) savanna east vs savanna south-central; o) savanna east vs savanna east.

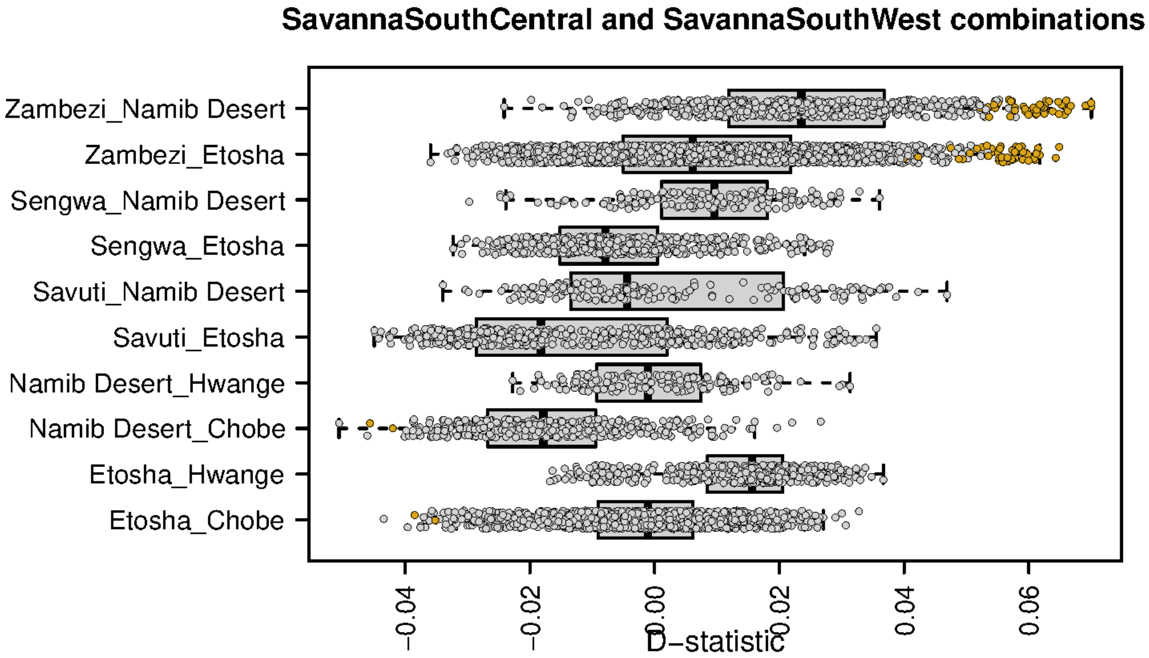

SavannaSouthCentral and SavannaSouthWest combinations
Zambezi_Namib Desert
Zambezi_Etosha
Sengwa_Namib Desert
Sengwa_Etosha
Savuti_Namib Desert
Savuti_Etosha
Namib Desert_Hwange
Namib Desert_Chobe
Etosha_Hwange
Etosha_Chobe
−0.04
−0.02
0.00
0.02
0.04
0.06
D−statistic

Fig. S7. b)

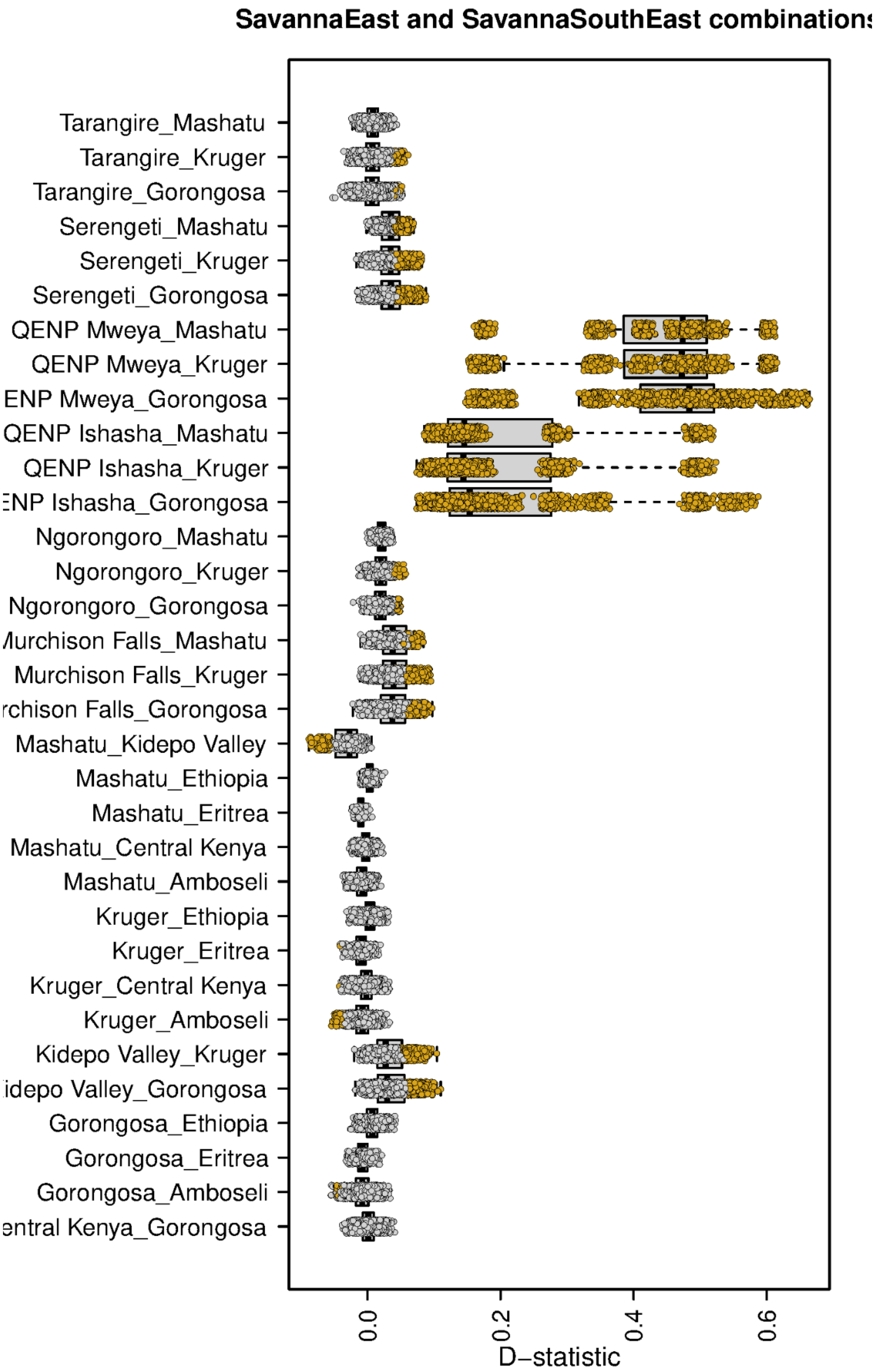
SavannaEast and SavannaSouthEast combination:
Tarangire_Mashatu
Tarangire_Kruger
Tarangire_Gorongosa
Serengeti_Mashatu
Serengeti_Kruger
Serengeti_Gorongosa
QENP Mweya_Mashatu
QENP Mweya_Kruger
ENP Mweya_Gorongosa
QENP Ishasha_Mashatu
QENP Ishasha_Kruger
ENP Ishasha_Gorongosa
Ngorongoro_Mashatu
Ngorongoro_Kruger
Ngorongoro_Gorongosa
/lurchison Falls_Mashatu
Murchison Falls_Kruger
rchison Falls_Gorongosa
Mashatu_Kidepo Valley
Mashatu_Ethiopia
Mashatu_Eritrea
Mashatu_Central Kenya
Mashatu_Amboseli
Kruger_Ethiopia
Kruger_Eritrea
Kruger_Central Kenya
Kruger_Amboseli
Kidepo Valley_Kruger
idepo Valley_Gorongosa
Gorongosa_Ethiopia
Gorongosa_Eritrea
Gorongosa_Amboseli
entral Kenya_Gorongosa
0.0
0.2
0.4
0.6
D−statistic

Fig. S7. c)

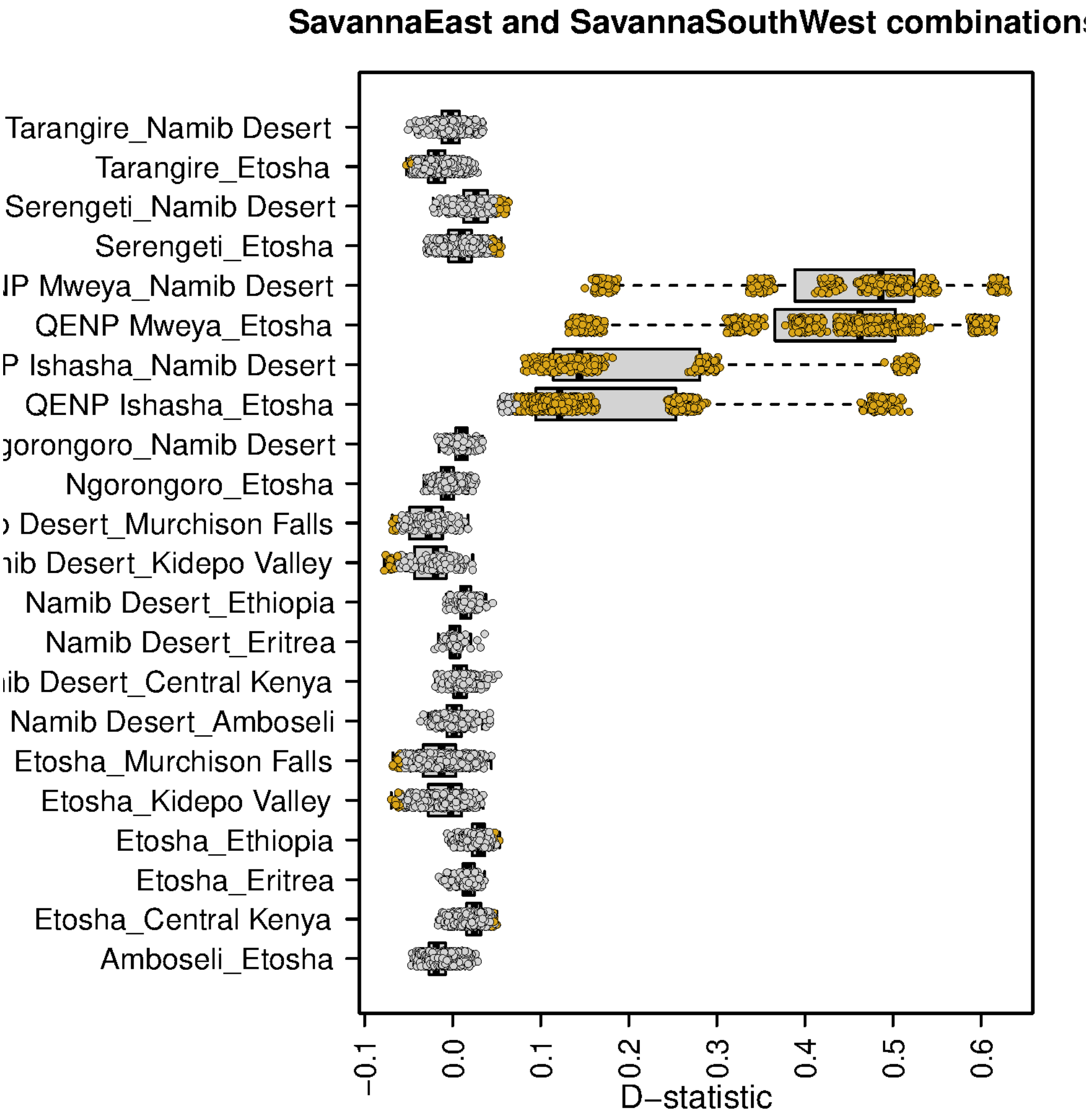
SavannaEast and SavannaSouthWest combination
Tarangire_Namib Desert
Tarangire_Etosha
Serengeti_Namib Desert
Serengeti_Etosha
IP Mweya_Namib Desert
QENP Mweya_Etosha
P Ishasha_Namib Desert
QENP Ishasha_Etosha
jorongoro_Namib Desert
Ngorongoro_Etosha
Desert_Murchison Falls
iib Desert_Kidepo Valley
Namib Desert_Ethiopia
Namib Desert_Eritrea
iib Desert_Central Kenya
Namib Desert_Amboseli
Etosha_Murchison Falls
Etosha_Kidepo Valley
Etosha_Ethiopia
Etosha_Eritrea
Etosha_Central Kenya
Amboseli_Etosha
−0.1
0.0
0.1
0.2
0.3
0.4
0.5
0.6
D−statistic

Fig. S7. d)

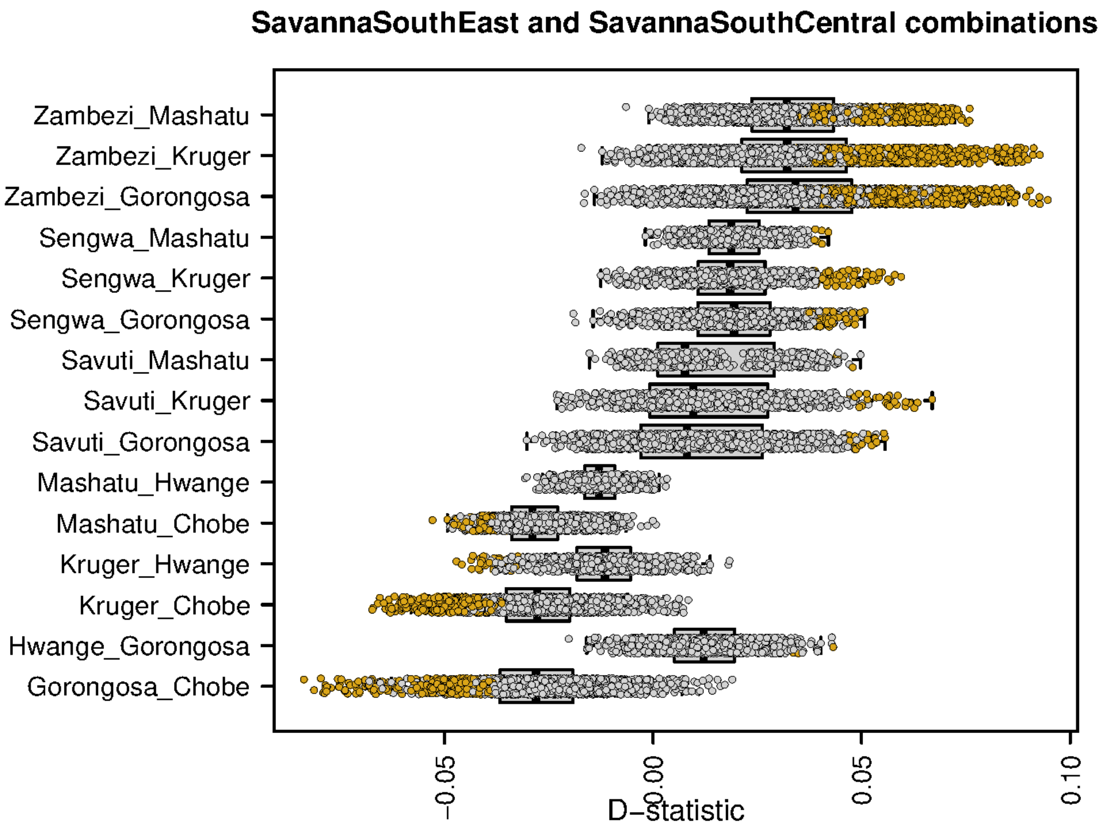

Fig. S7. e)

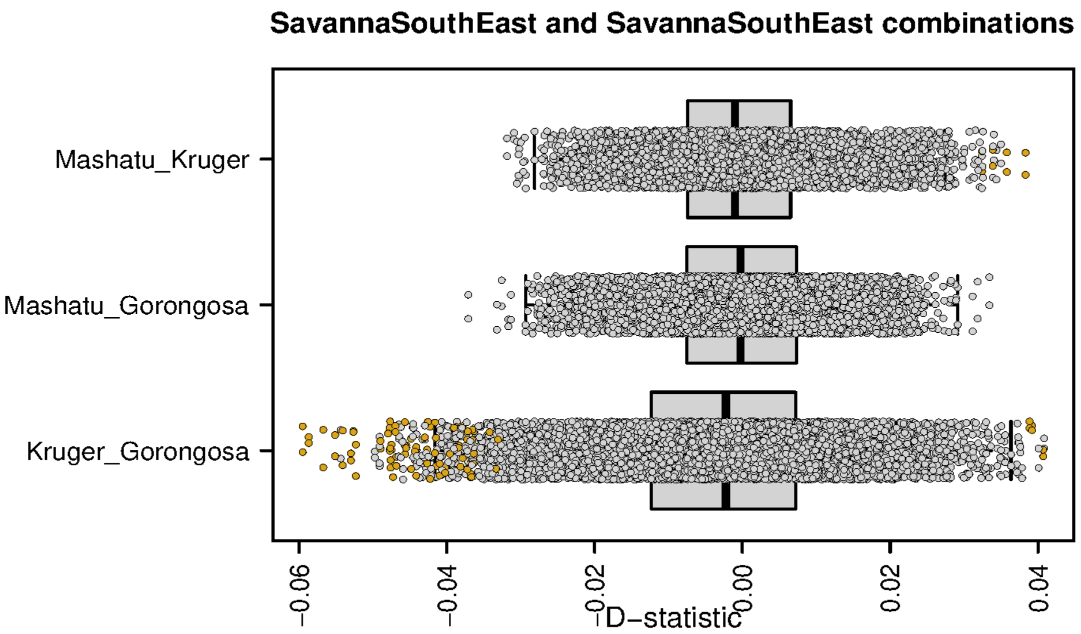

Fig. S7. f)

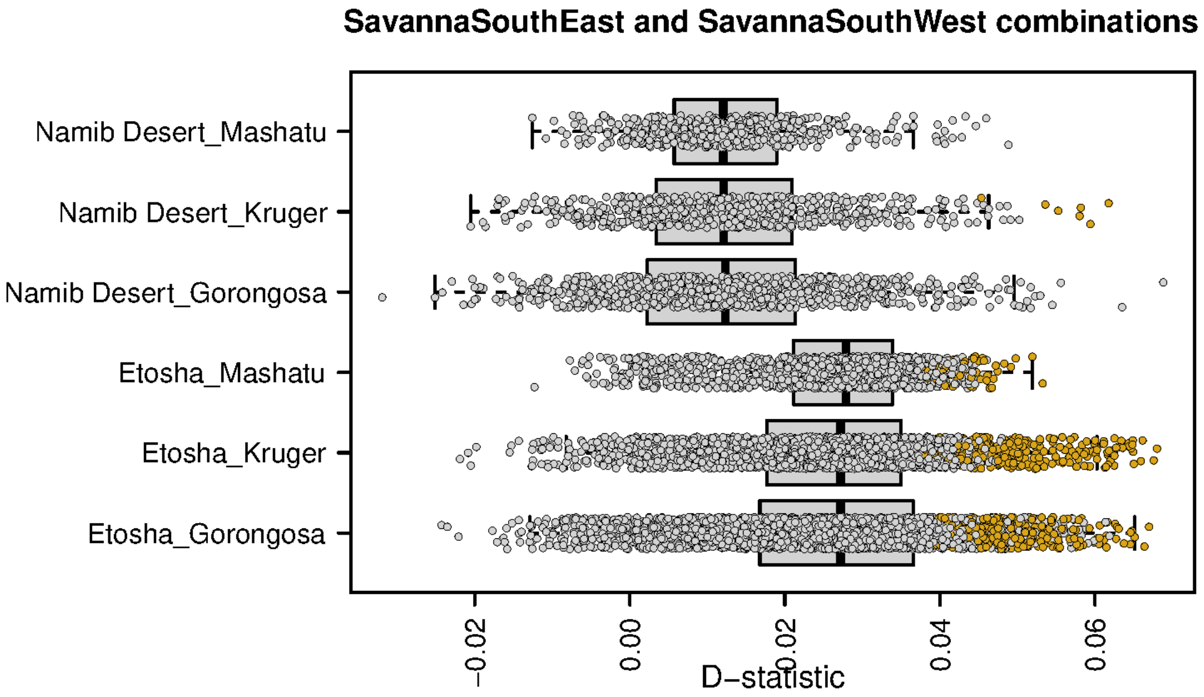

Fig. S7. g)

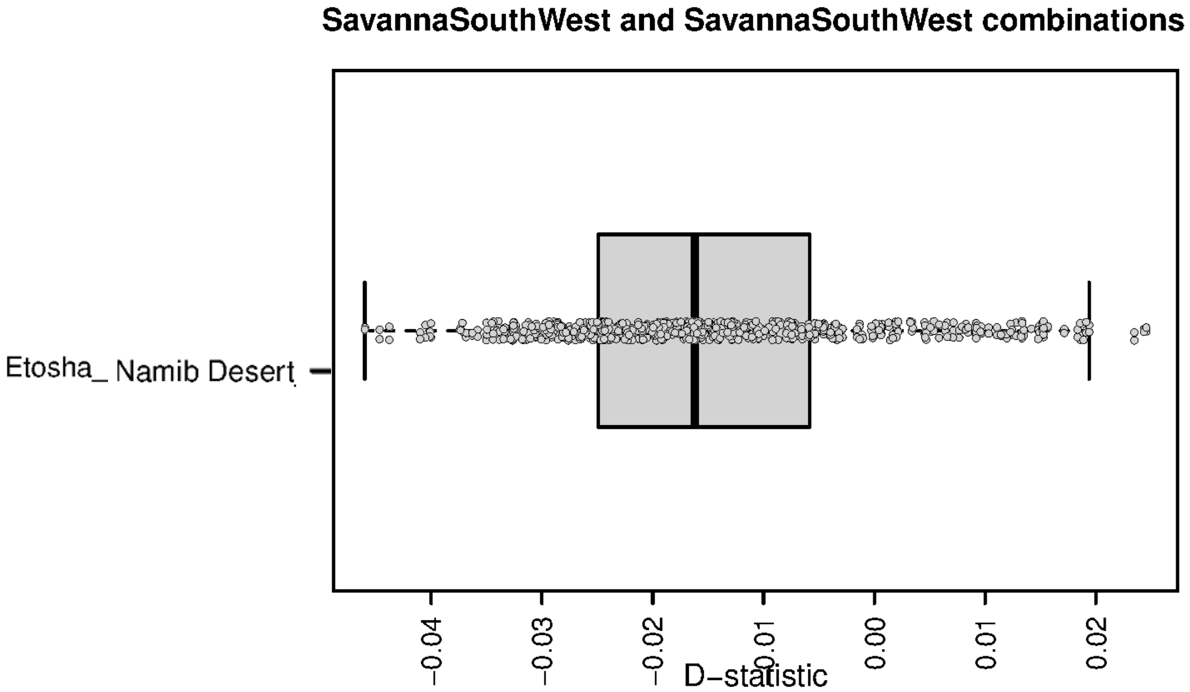

Fig. S7. h)

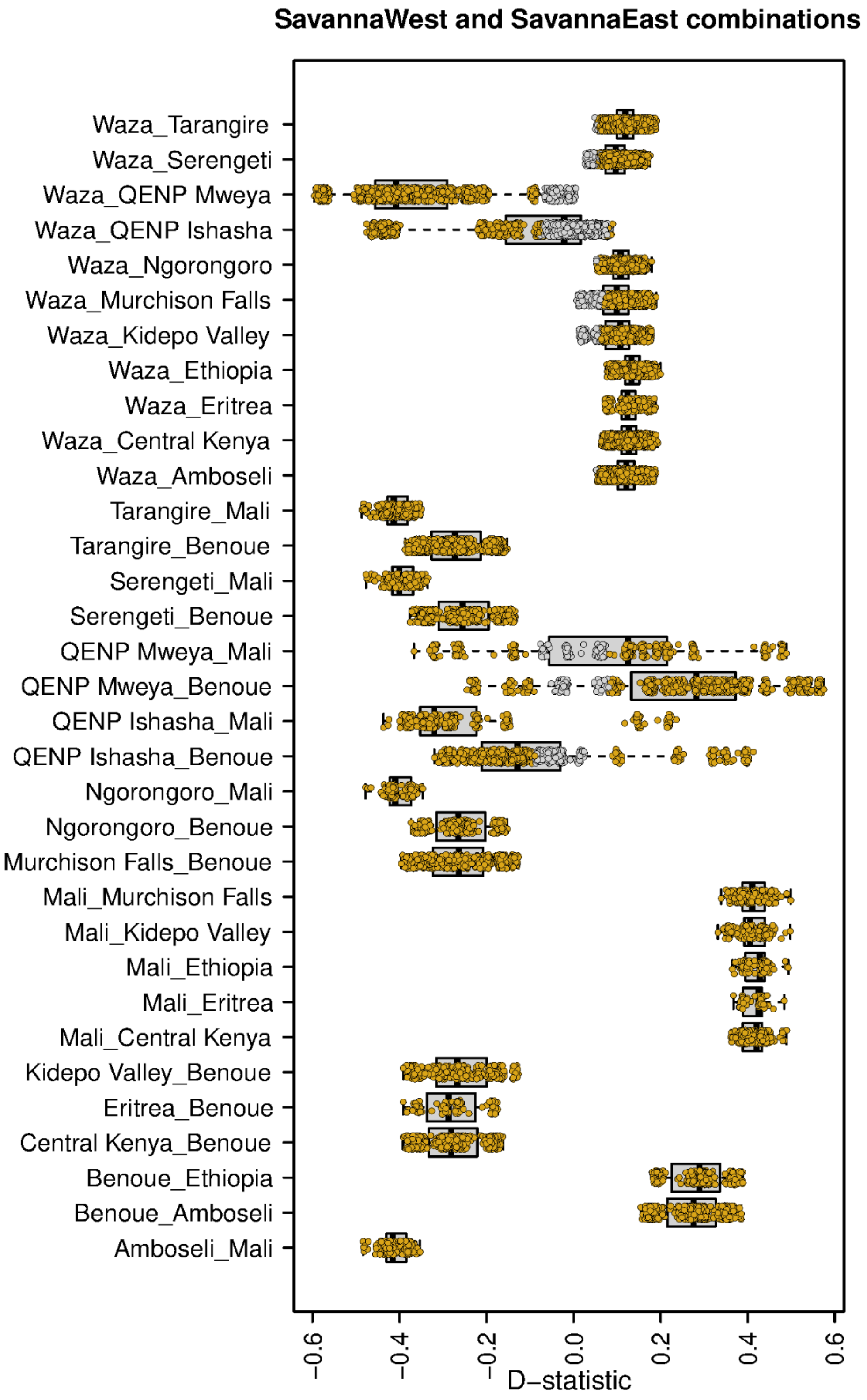
SavannaWest and SavannaEast combinations
Waza_Tarangire
Waza_Serengeti
Waza_QENP Mweya
Waza_QENP Ishasha
Waza_Ngorongoro
Waza_Murchison Falls
Waza_Kidepo Valley
Waza_Ethiopia
Waza_Eritrea
Waza_Central Kenya
Waza_Amboseli
Tarangire_Mali
Tarangire_Benoue
Serengeti_Mali
Serengeti_Benoue
QENP Mweya_Mali
QENP Mweya_Benoue
QENP Ishasha_Mali
QENP Ishasha_Benoue
Ngorongoro_Mali
Ngorongoro_Benoue
Murchison Falls_Benoue
Mali_Murchison Falls
Mali_Kidepo Valley
Mali_Ethiopia
Mali_Eritrea
Mali_Central Kenya
Kidepo Valley_Benoue
Eritrea_Benoue
Central Kenya_Benoue
Benoue_Ethiopia
Benoue_Amboseli
Amboseli_Mali
−0.6
−0.4
−0.2
0.0
0.2
0.4
0.6
D−statistic

Fig. S7. i)

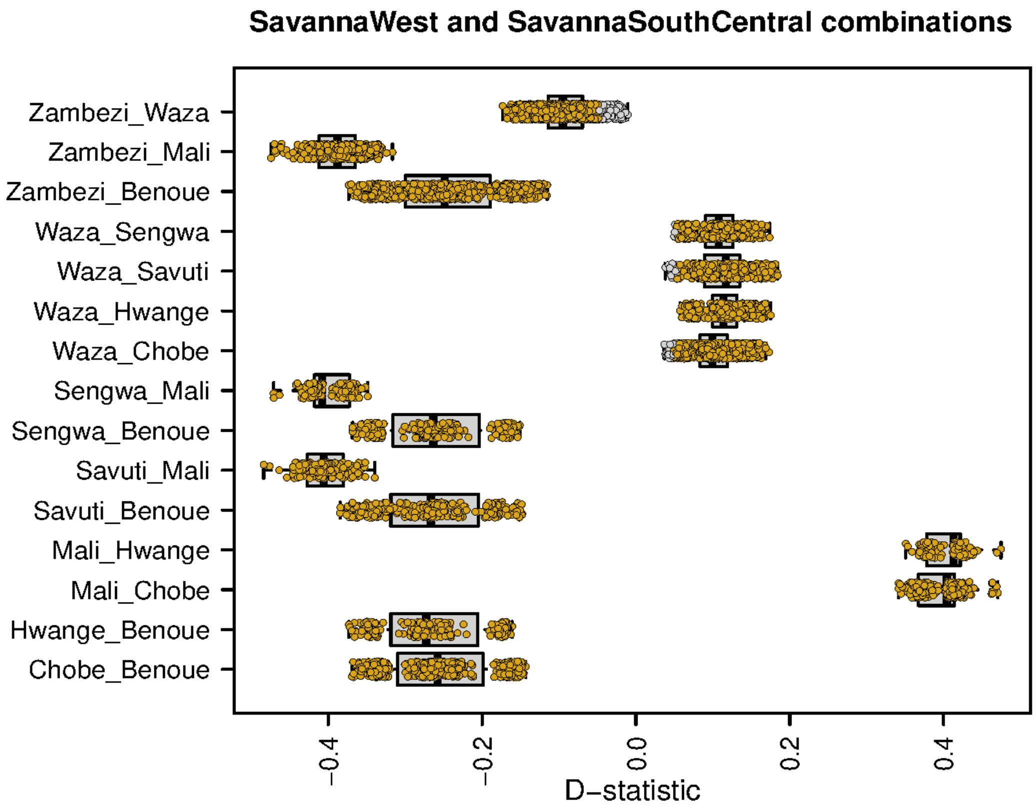

Fig. S7. j)

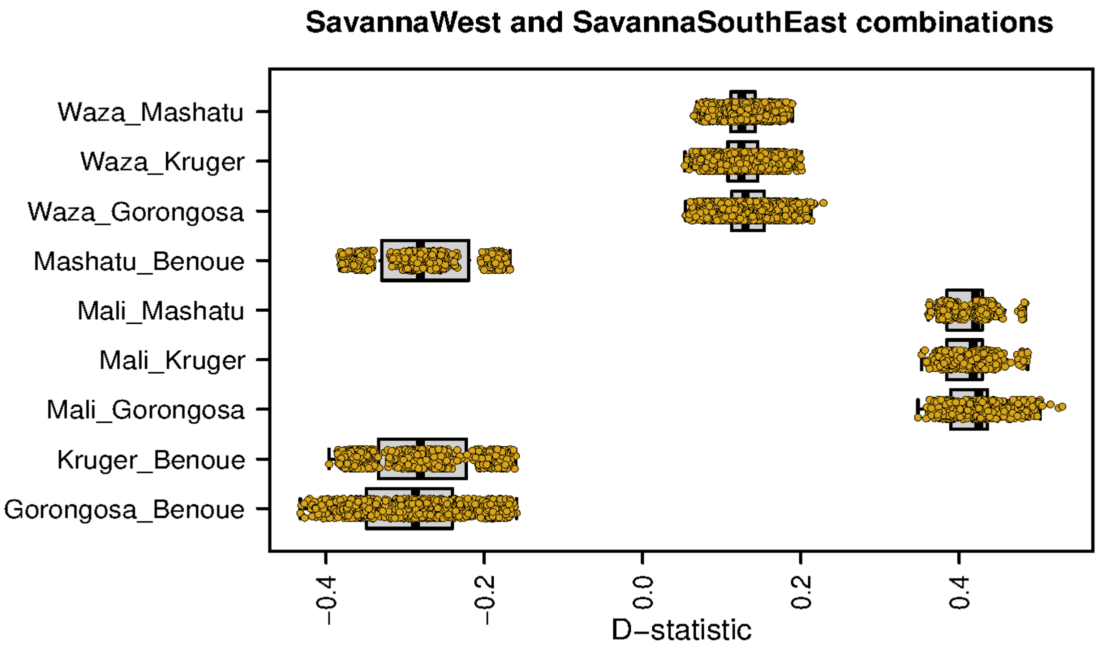

Fig. S7. k)

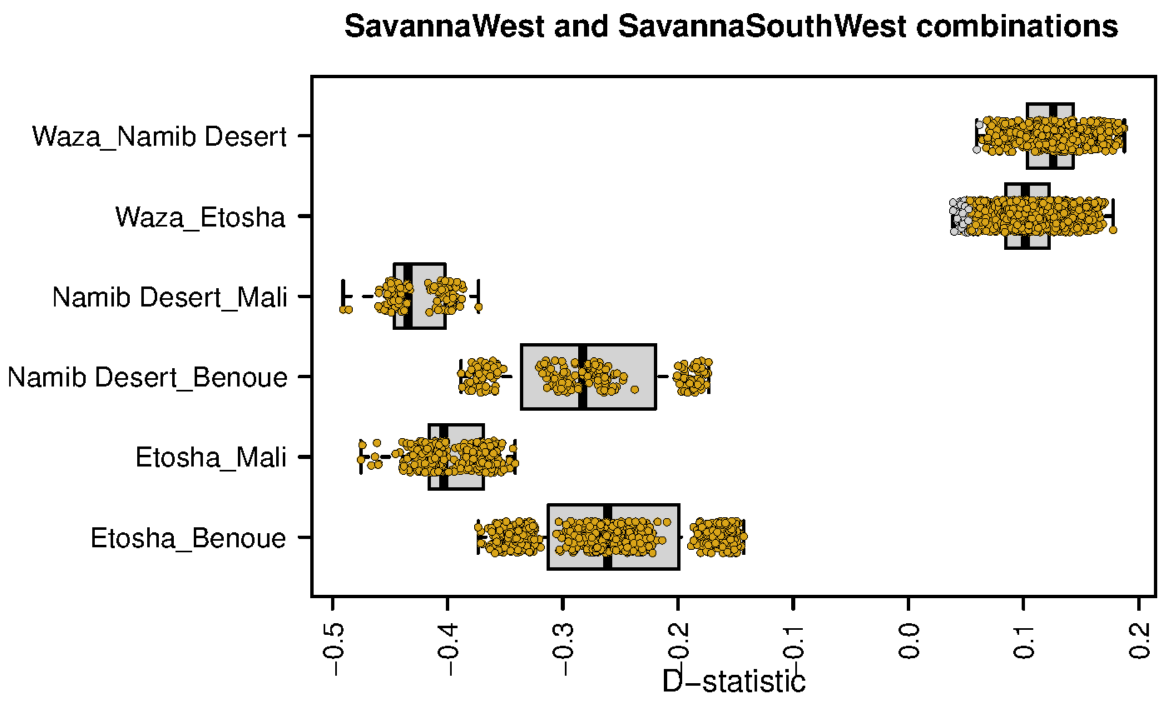

Fig. S7. l)

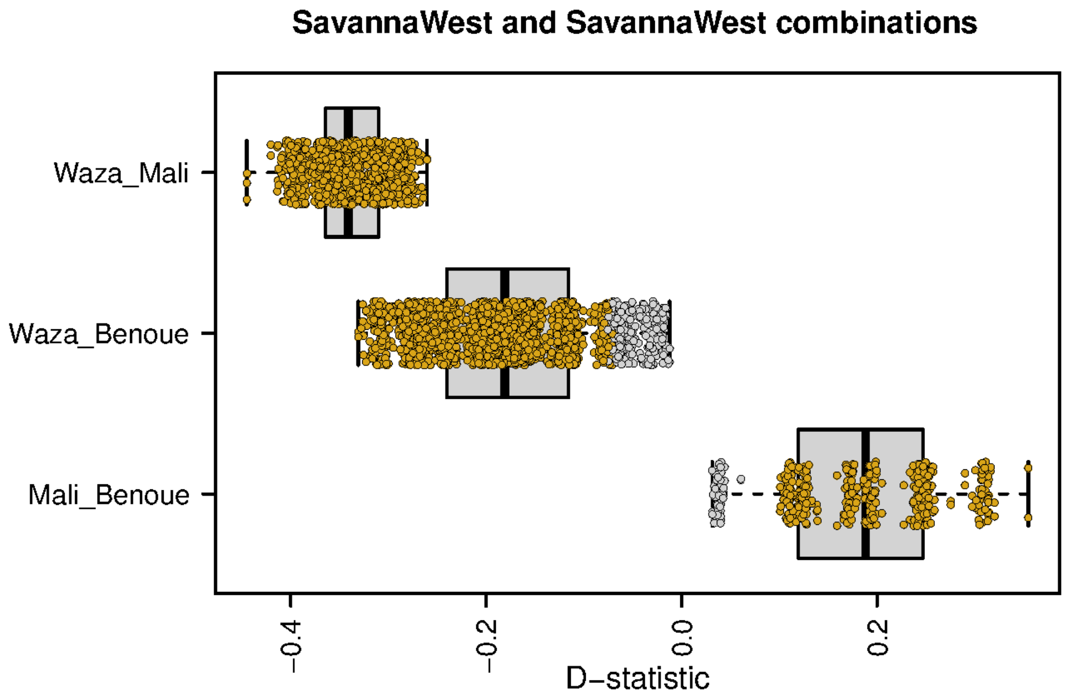

Fig. S7. m)

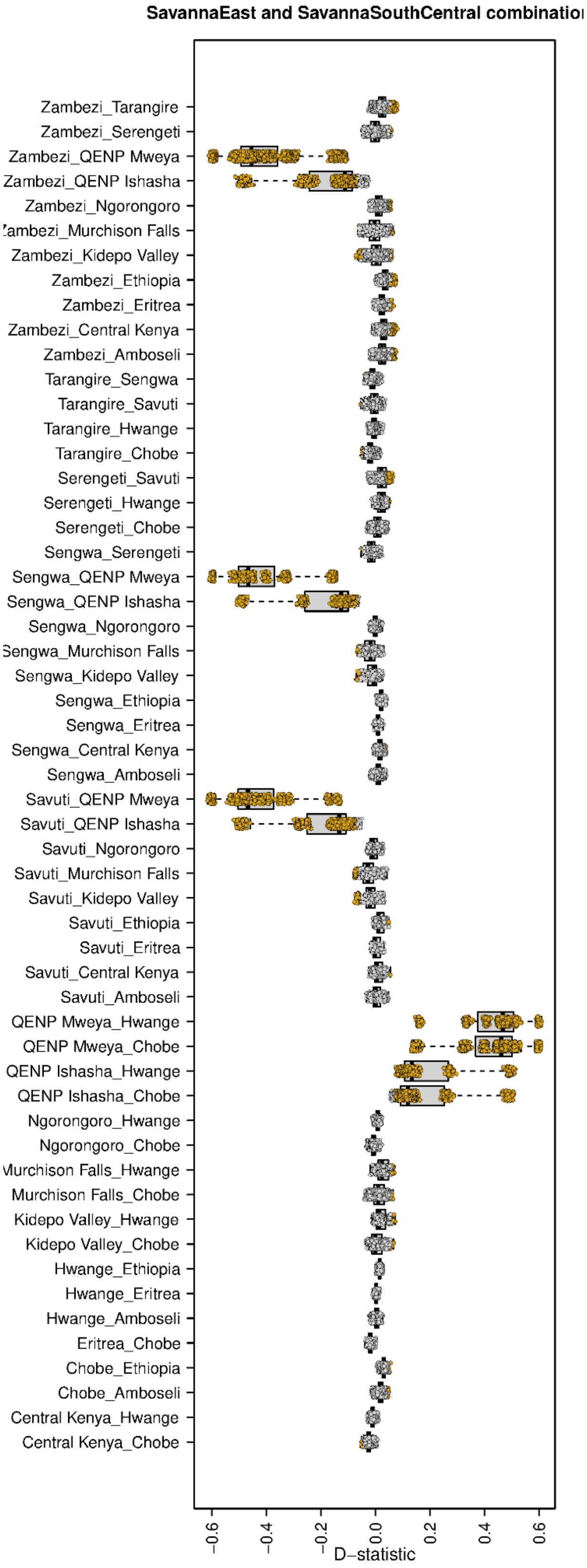
SavannaEast and SavannaSouthCentral combinatio
Zambezi_Tarangire
Zambezi_Serengeti
Zambezi_QENP Mweya
Zambezi_QENP Ishasha
Zambezi_Ngorongoro
'ambezi_Murchison Falls
Zambezi_Kidepo Valley
Zambezi_Ethiopia
Zambezi_Eritrea
Zambezi_Central Kenya
Zambezi_Amboseli
Tarangire_Sengwa
Tarangire_Savuti
Tarangire_Hwange
Tarangire_Chobe
Serengeti_Savuti
Serengeti_Hwange
Serengeti_Chobe
Sengwa_Serengeti
Sengwa_QENP Mweya
Sengwa_QENP Ishasha
Sengwa_Ngorongoro
Sengwa_Murchison Falls
Sengwa_Kidepo Valley
Sengwa_Ethiopia
Sengwa_Eritrea
Sengwa_Central Kenya
Sengwa_Amboseli
Savuti_QENP Mweya
Savuti_QENP Ishasha
Savuti_Ngorongoro
Savuti_Murchison Falls
Savuti_Kidepo Valley
Savuti_Ethiopia
Savuti_Eritrea
Savuti_Central Kenya
Savuti_Amboseli
QENP Mweya_Hwange
QENP Mweya_Chobe
QENP Ishasha_Hwange
QENP Ishasha_Chobe
Ngorongoro_Hwange
Ngorongoro_Chobe
Murchison Falls_Hwange
Murchison Falls_Chobe
Kidepo Valley_Hwange
Kidepo Valley_Chobe
Hwange_Ethiopia
Hwange_Eritrea
Hwange_Amboseli
Eritrea_Chobe
Chobe_Ethiopia
Chobe_Amboseli
Central Kenya_Hwange
Central Kenya_Chobe
−0.6
−0.4
−0.2
0.0
0.2
0.4
0.6
D−statistic

Fig. S7. n)

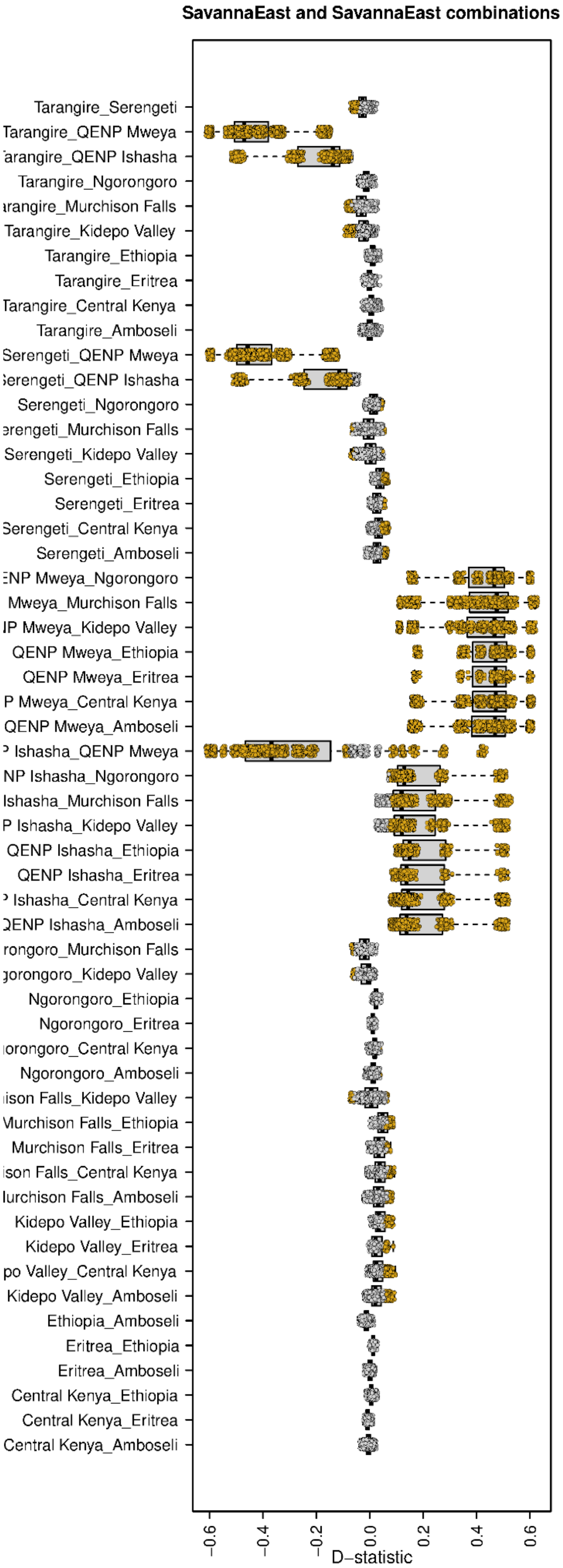
SavannaEast and SavannaEast combinations
Tarangire_Serengeti
Tarangire_QENP Mweya
'arangire_QENP Ishasha
Tarangire_Ngorongoro
ırangire_Murchison Falls
Tarangire_Kidepo Valley
Tarangire_Ethiopia
Tarangire_Eritrea
Tarangire_Central Kenya
Tarangire_Amboseli
Serengeti_QENP Mweya
Serengeti_QENP Ishasha
Serengeti_Ngorongoro
erengeti_Murchison Falls
Serengeti_Kidepo Valley
Serengeti_Ethiopia
Serengeti_Eritrea
Serengeti_Central Kenya
Serengeti_Amboseli
ENP Mweya_Ngorongoro
Mweya_Murchison Falls
IP Mweya_Kidepo Valley
QENP Mweya_Ethiopia
QENP Mweya_Eritrea
P Mweya_Central Kenya
QENP Mweya_Amboseli
P Ishasha_QENP Mweya
NP Ishasha_Ngorongoro
Ishasha_Murchison Falls
P Ishasha_Kidepo Valley
QENP Ishasha_Ethiopia
QENP Ishasha_Eritrea
P Ishasha_Central Kenya
QENP Ishasha_Amboseli
rongoro_Murchison Falls
gorongoro_Kidepo Valley
Ngorongoro_Ethiopia
Ngorongoro_Eritrea
orongoro_Central Kenya
Ngorongoro_Amboseli
ison Falls_Kidepo Valley
Murchison Falls_Ethiopia
Murchison Falls_Eritrea
ison Falls_Central Kenya
lurchison Falls_Amboseli
Kidepo Valley_Ethiopia
Kidepo Valley_Eritrea
po Valley_Central Kenya
Kidepo Valley_Amboseli
Ethiopia_Amboseli
Eritrea_Ethiopia
Eritrea_Amboseli
Central Kenya_Ethiopia
Central Kenya_Eritrea
Central Kenya_Amboseli
−0.6
−0.4
−0.2
0.0
0.2
0.4
0.6
D−statistic

Fig. S7. o)

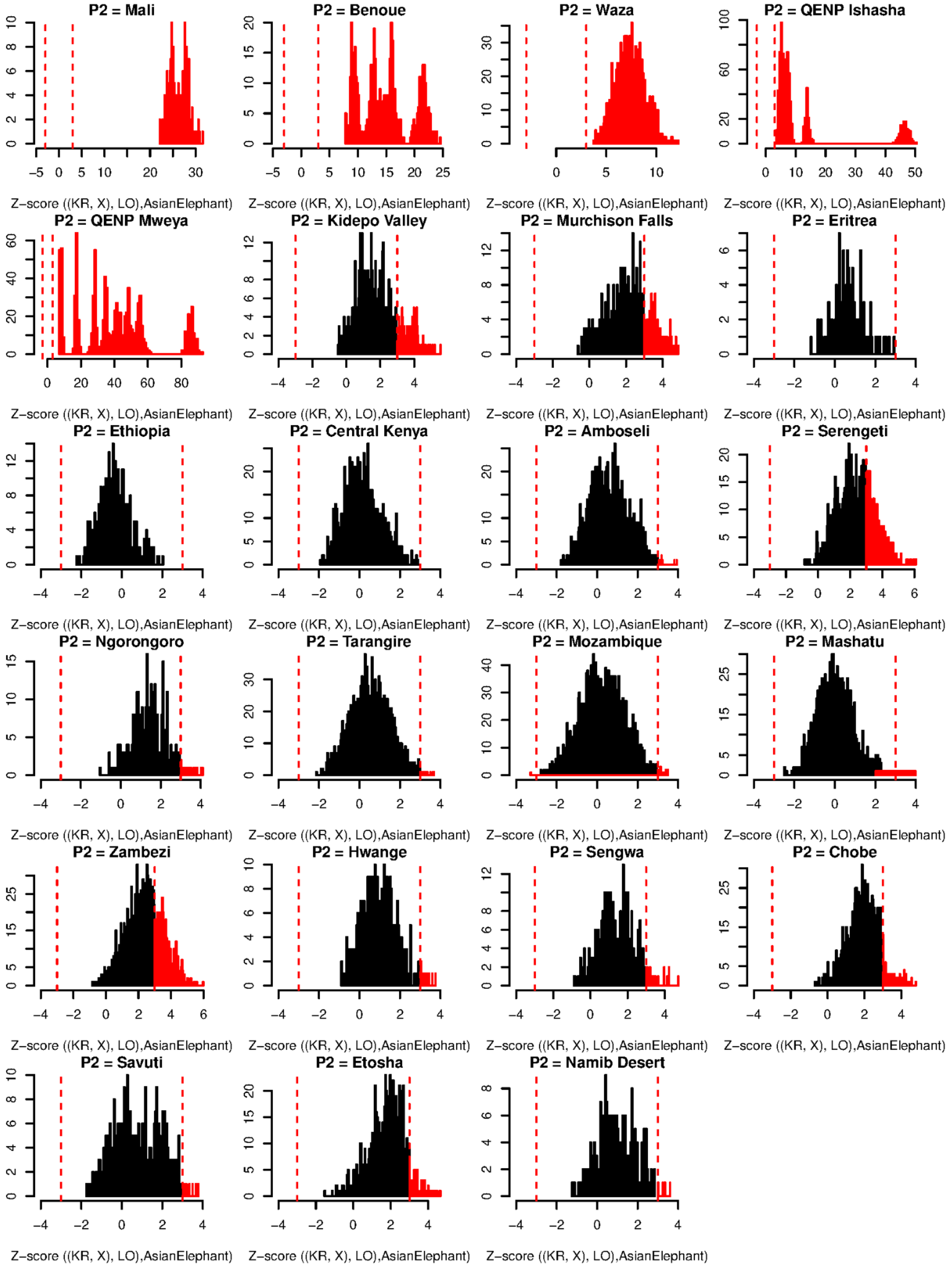

Fig. S8. Histogram of all Z-scores from the D-statistics analysis (Fig. S7) per locality, with significant scores in red.

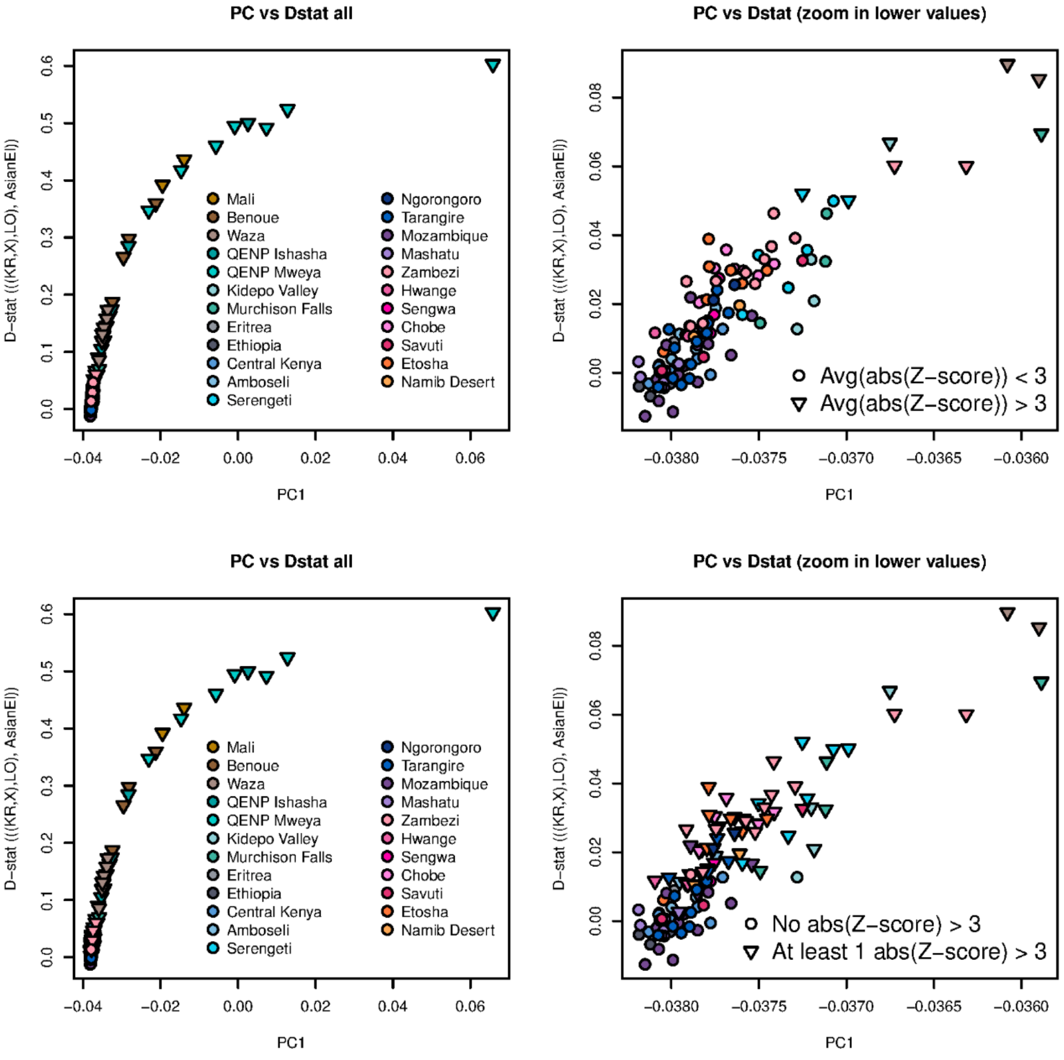

Fig. S9. The relationship between values at the principal component 1 (PC1) against the *D*-statistics. Triangles indicate statistical significance (otherwise a circle is used), with the top two panels showing significance if the average absolute Z-score across all comparisons for a given population is above 3 and the bottom two panels showing significance if the absolute Z-score is above 3 in any of the comparisons.

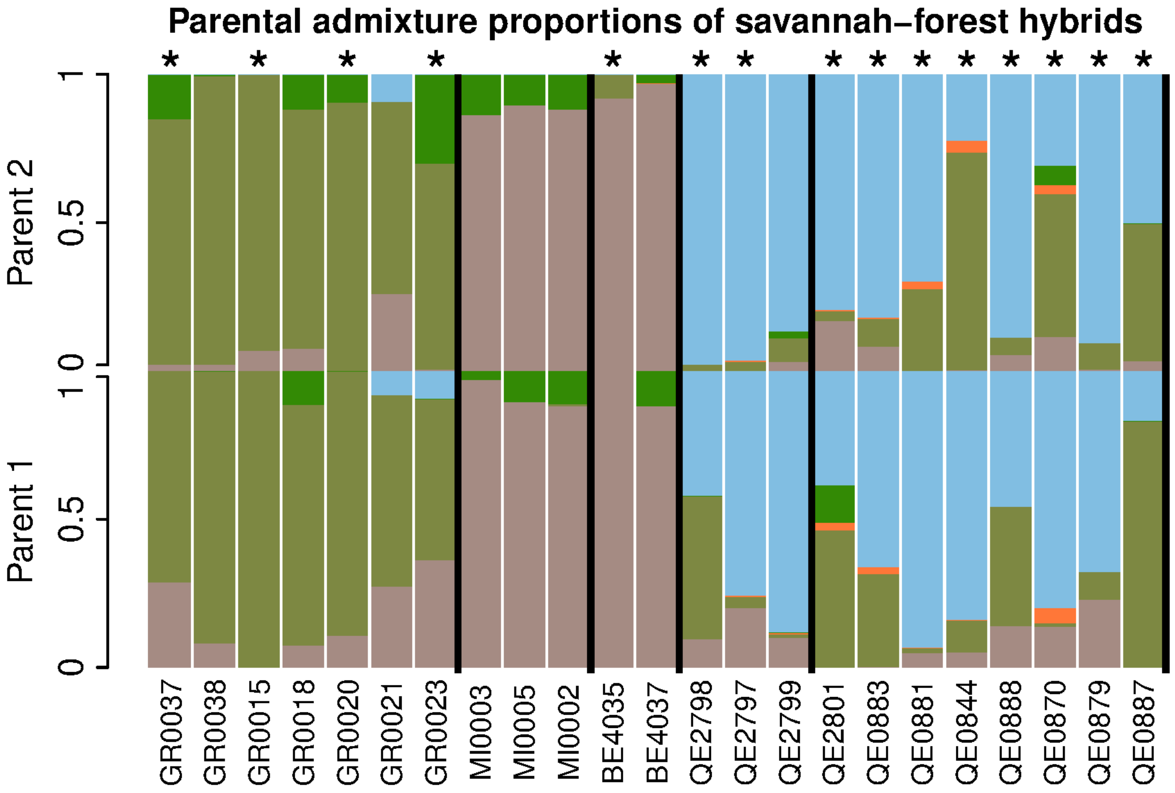

Fig. S10. Analysis of recent admixture in *apoh*[17]. For each individual with interspecific admixture when assuming $K = 5$ in the admixture analyses, the estimated admixture proportions of each of its parents are shown, with colors corresponding to the clusters shown in Figure 2b at $K = 5$. Different admixture proportions in the parents are indicative that admixture is recent. An asterisk indicates individuals for which the expected parental ancestry proportions under a pedigree with recent admixture are closer to the observed estimates than a pedigree without recent admixture.

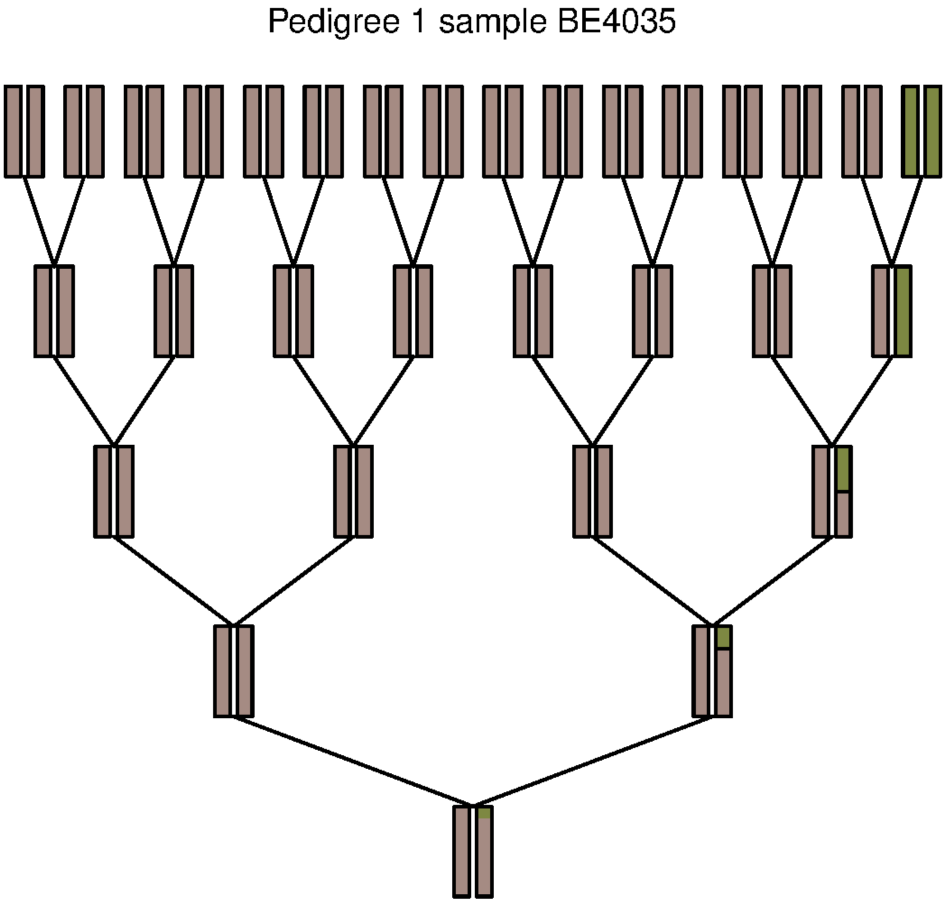

Fig. S11. Individual pedigrees from in *apoh*[17] that best fit the estimated parental admixture proportions of genomes with evidence of recent admixture 2-6 generations ago (marked with an asterisk in Figure S10). The individuals are colored by their admixture proportion, with colors corresponding to the clusters found in Figure 2b at $K = 5$. Pedigrees for specimens: a) BE4035; b) GR0015; c) GR0020; d) GR0023; e) GR0037; f) QE0844; g) QE0870; h) QE0881; i) QE0883; j) QE0887; k) QE0887; l) QE2798; m) QE2801.

Pedigree 1 sample GR0015

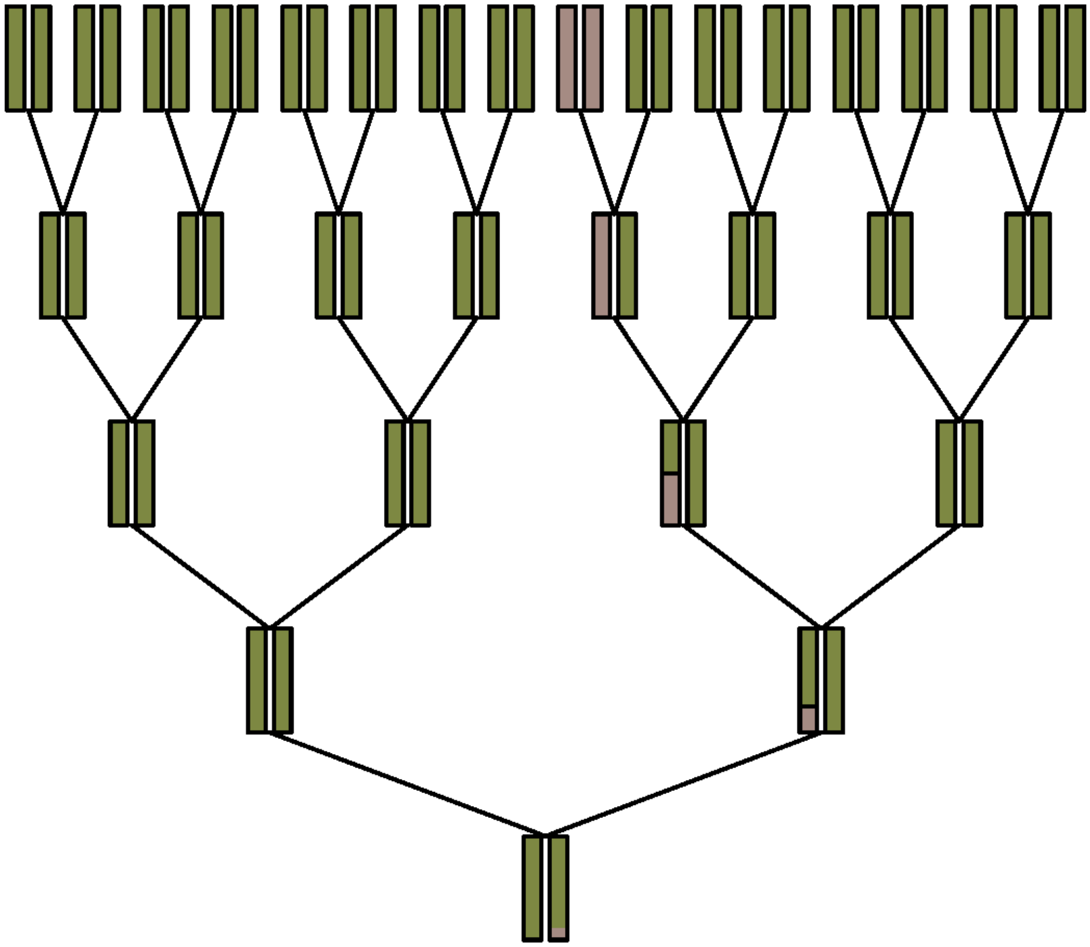

Fig. S1.1b)

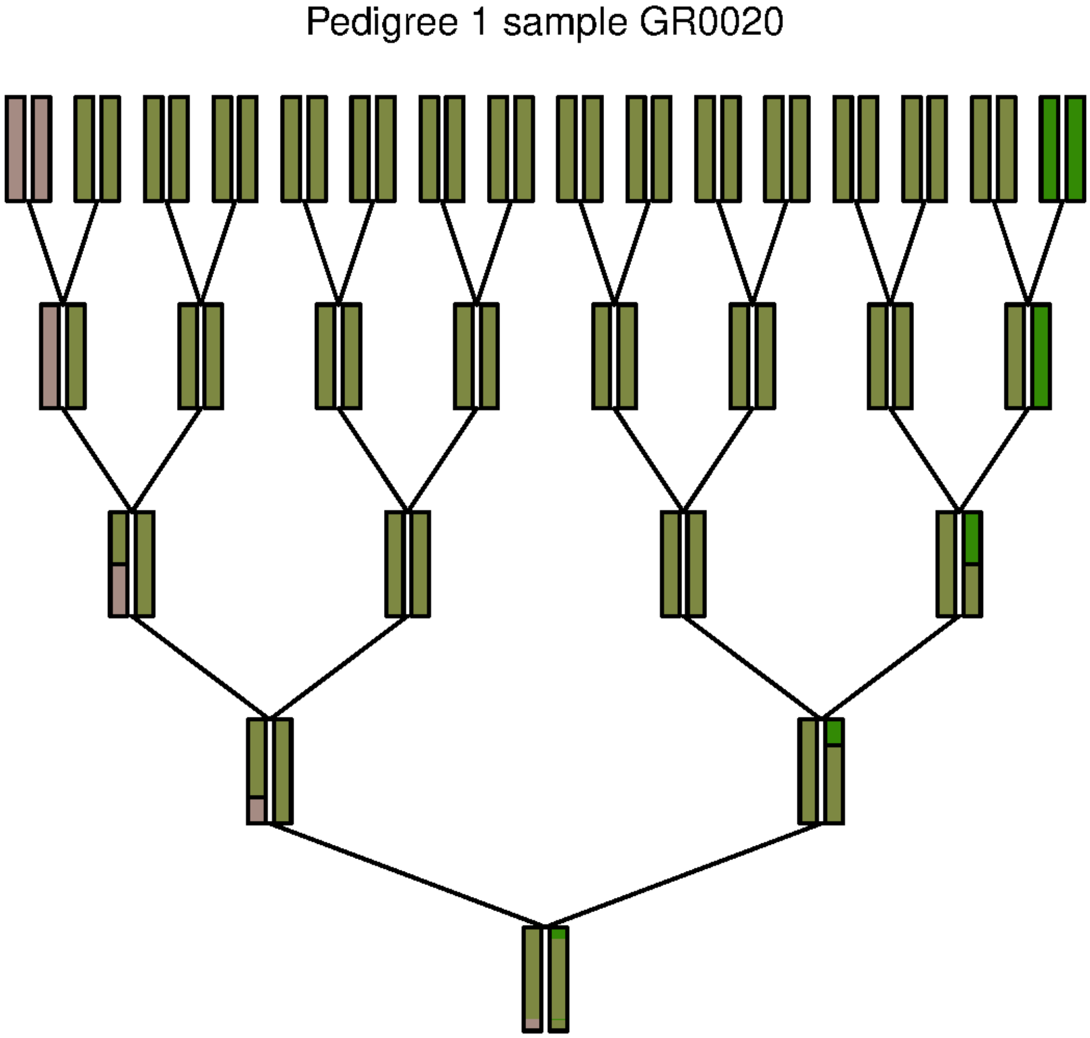

Fig. S11. c)

Pedigree 1 sample GR0023

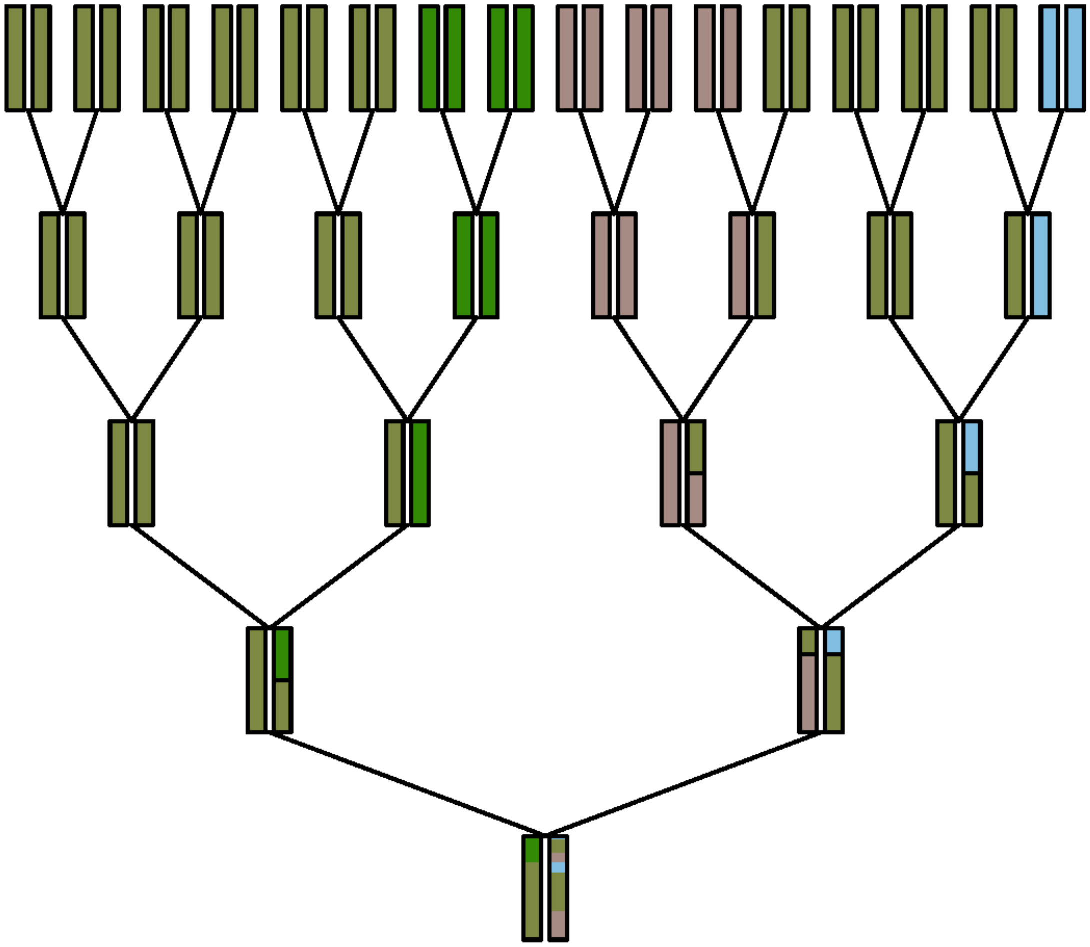

Fig. S11. d)

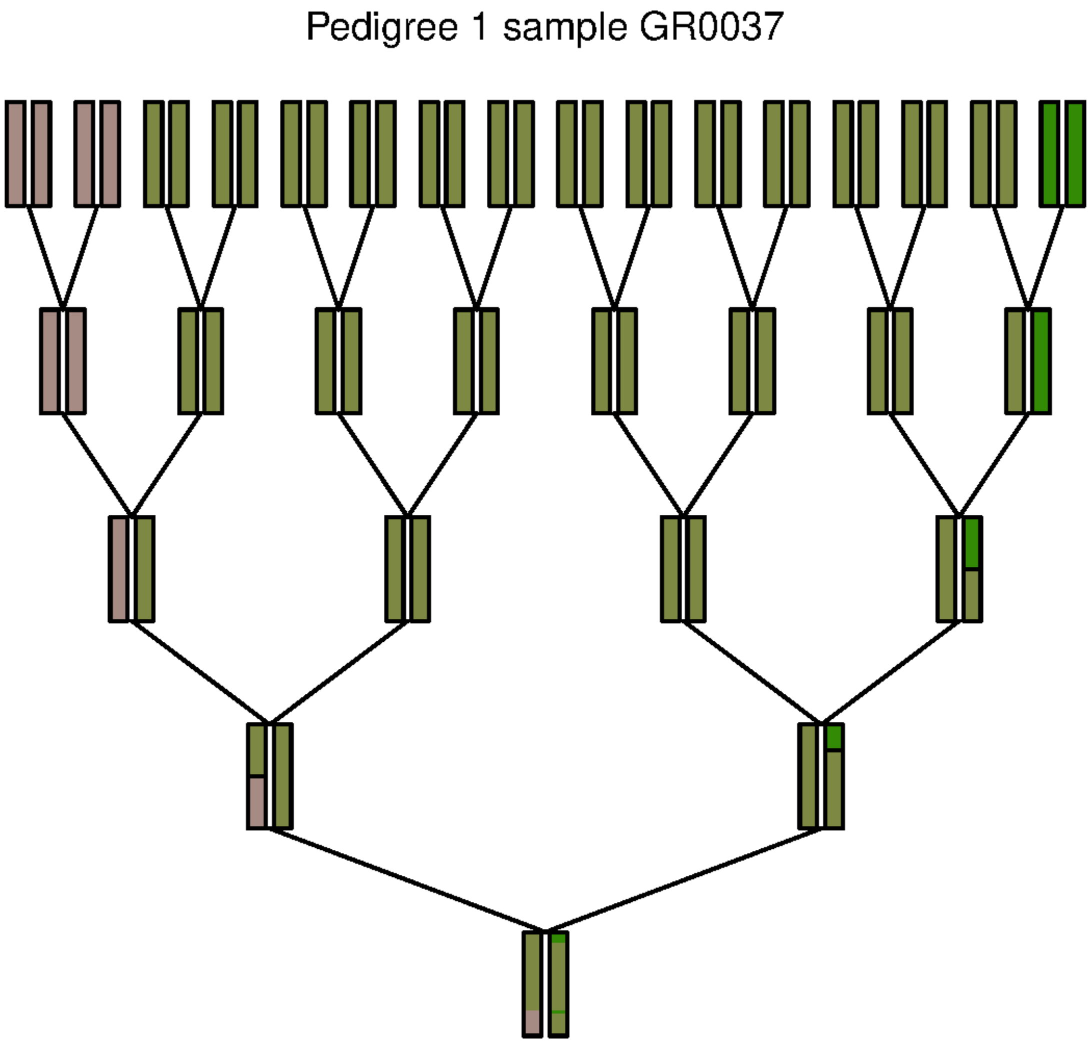

Fig. S11. e)

Pedigree 1 sample QE0844

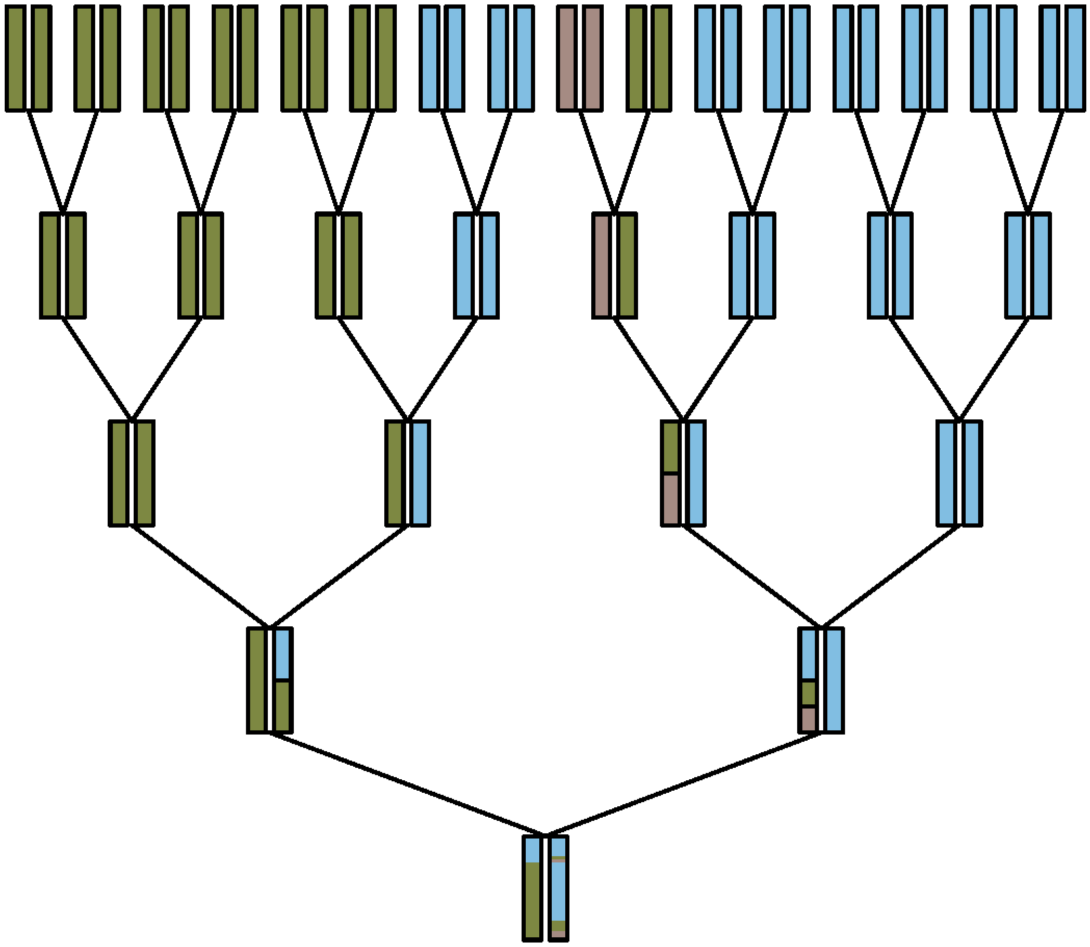

Fig. S11. f)

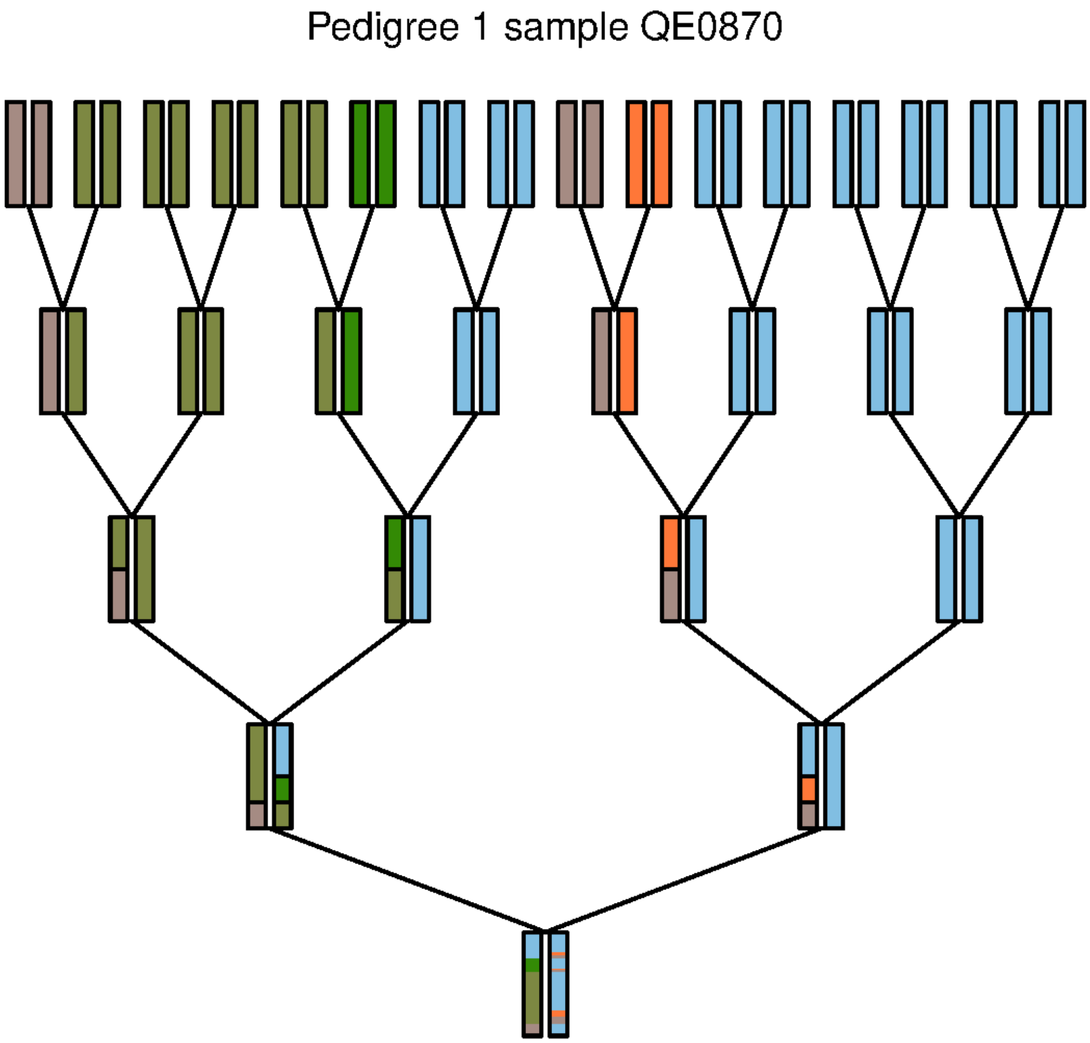

Fig. S11. g)

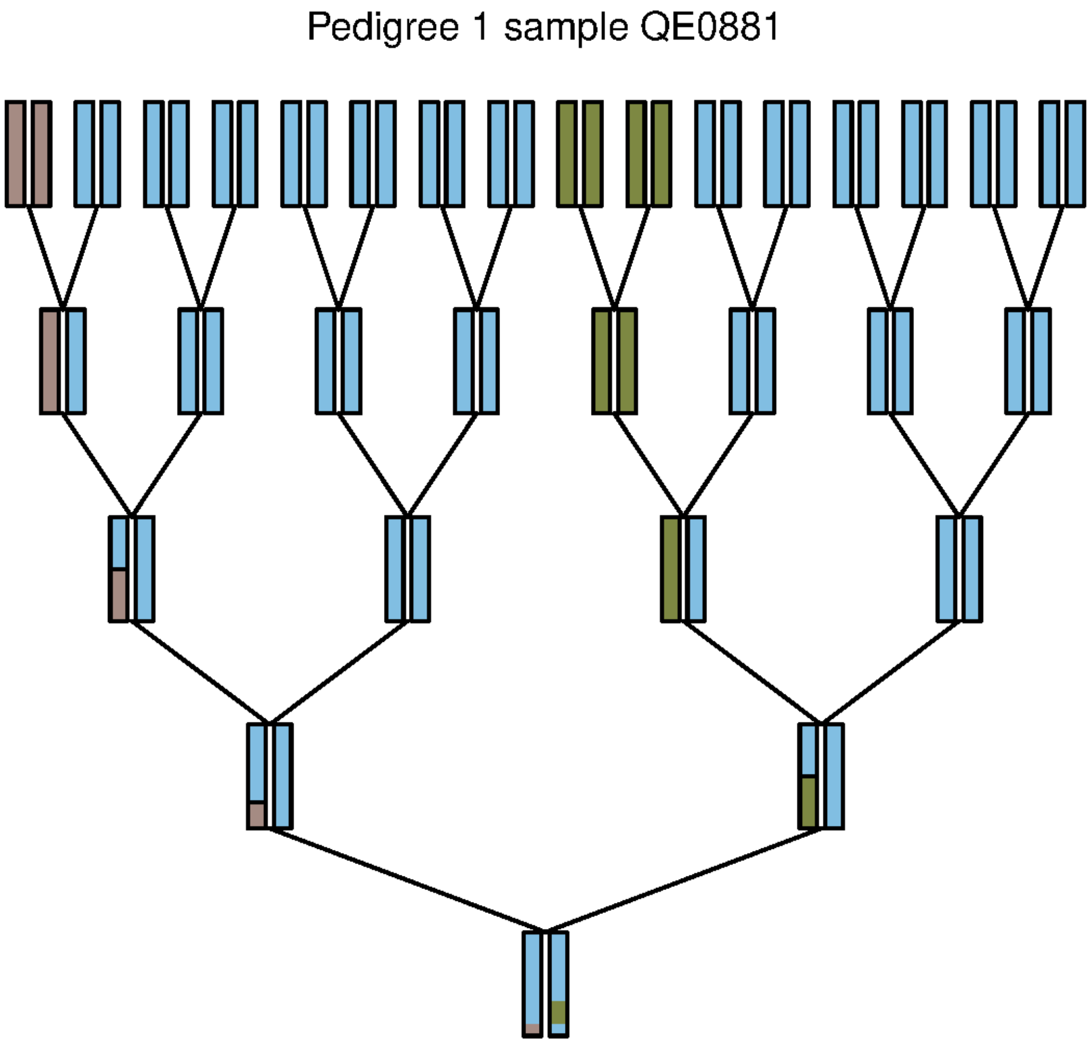

Fig. S11. h)

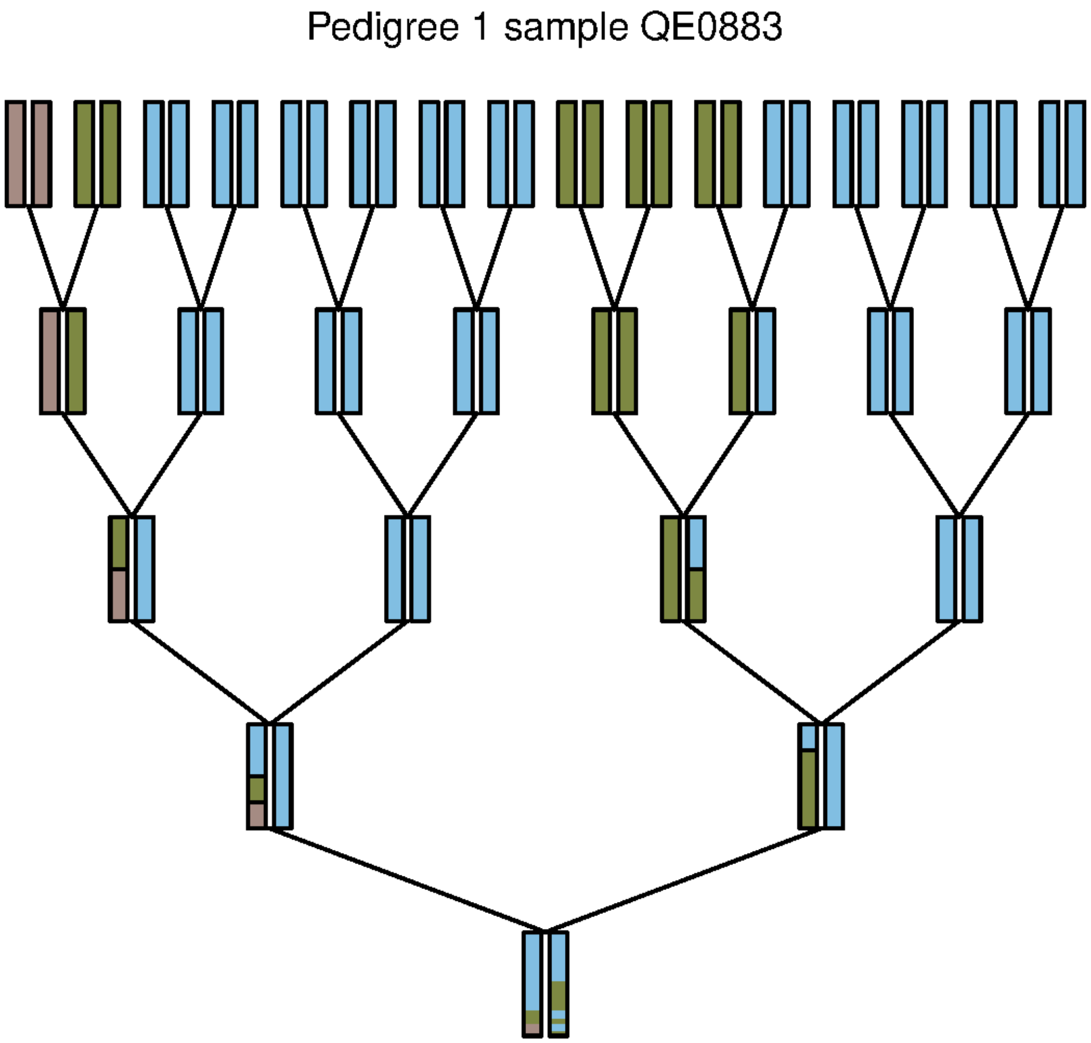

Fig. S11. i)

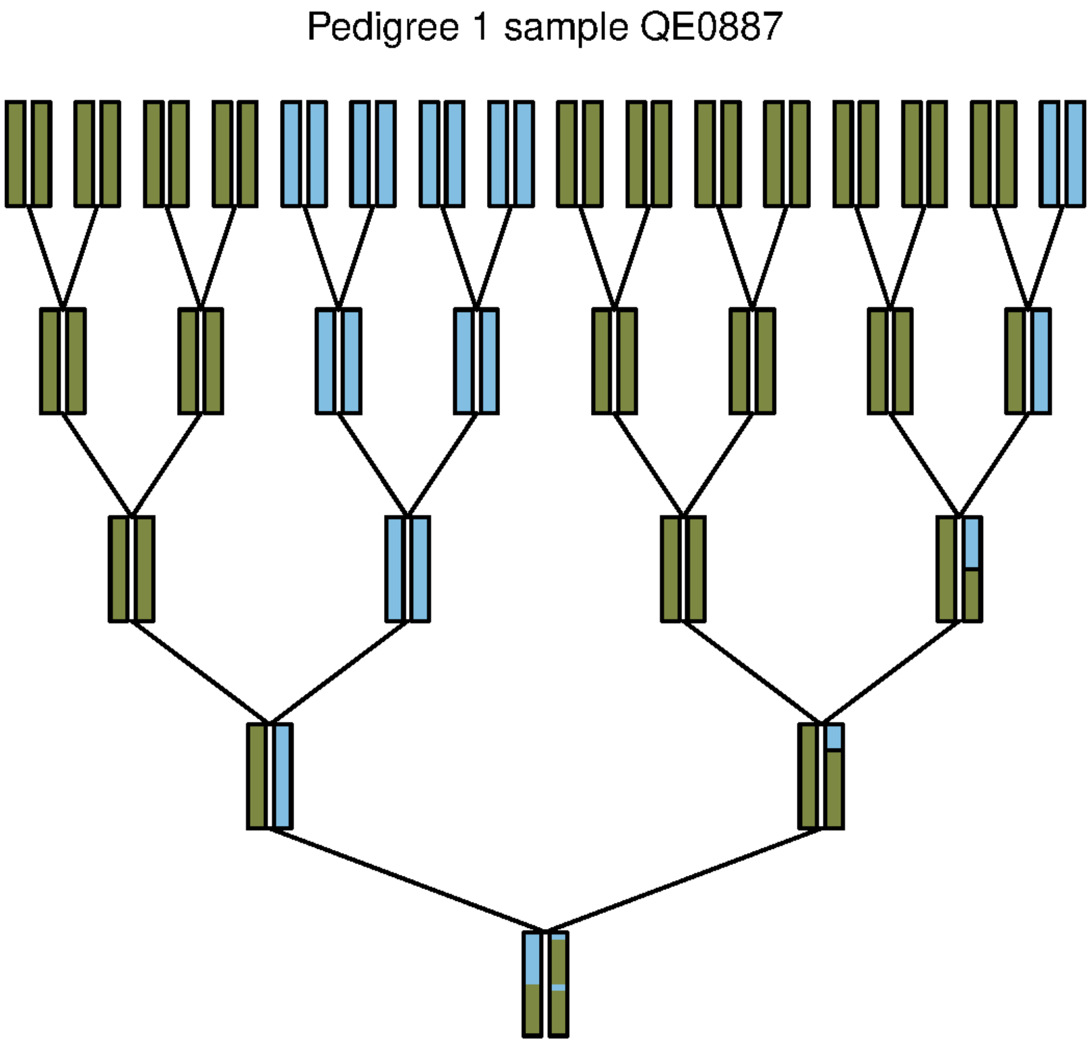

Fig. S11. j)

Pedigree 1 sample QE0888

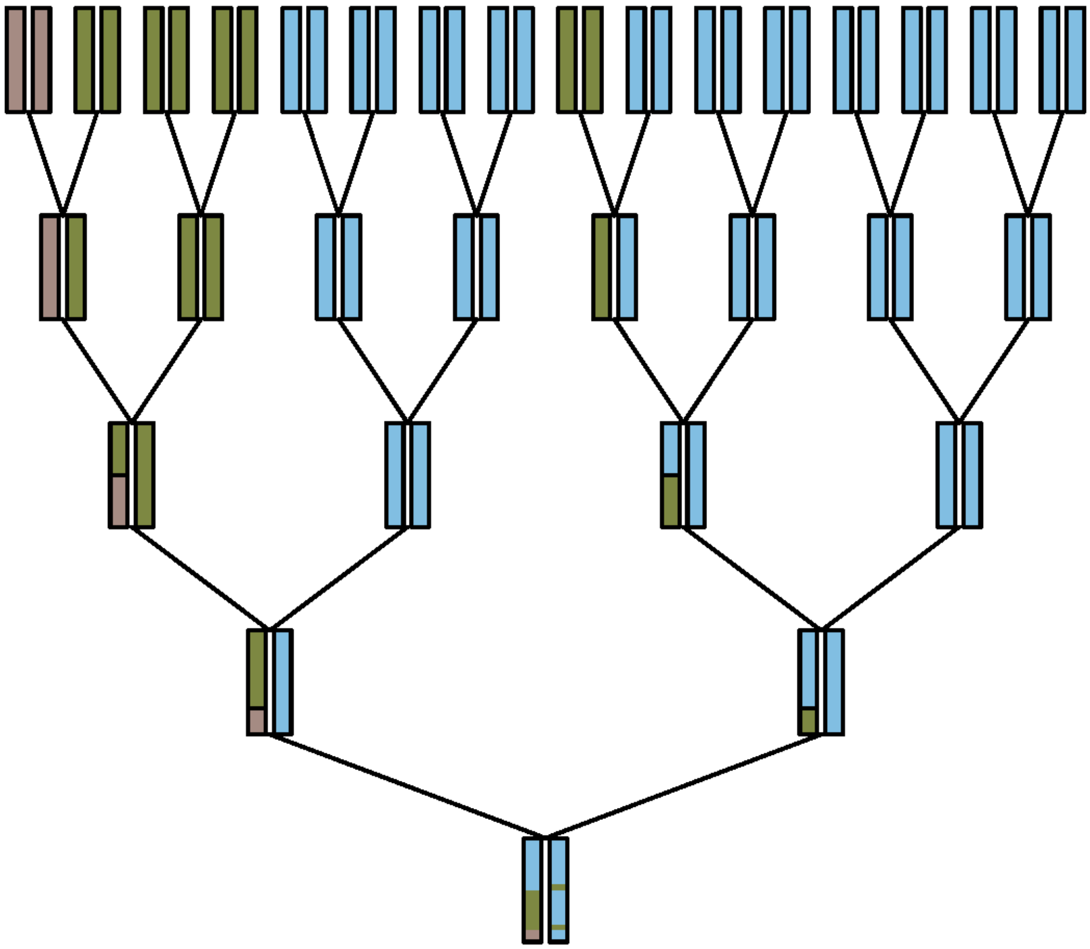

Fig. S11. k)

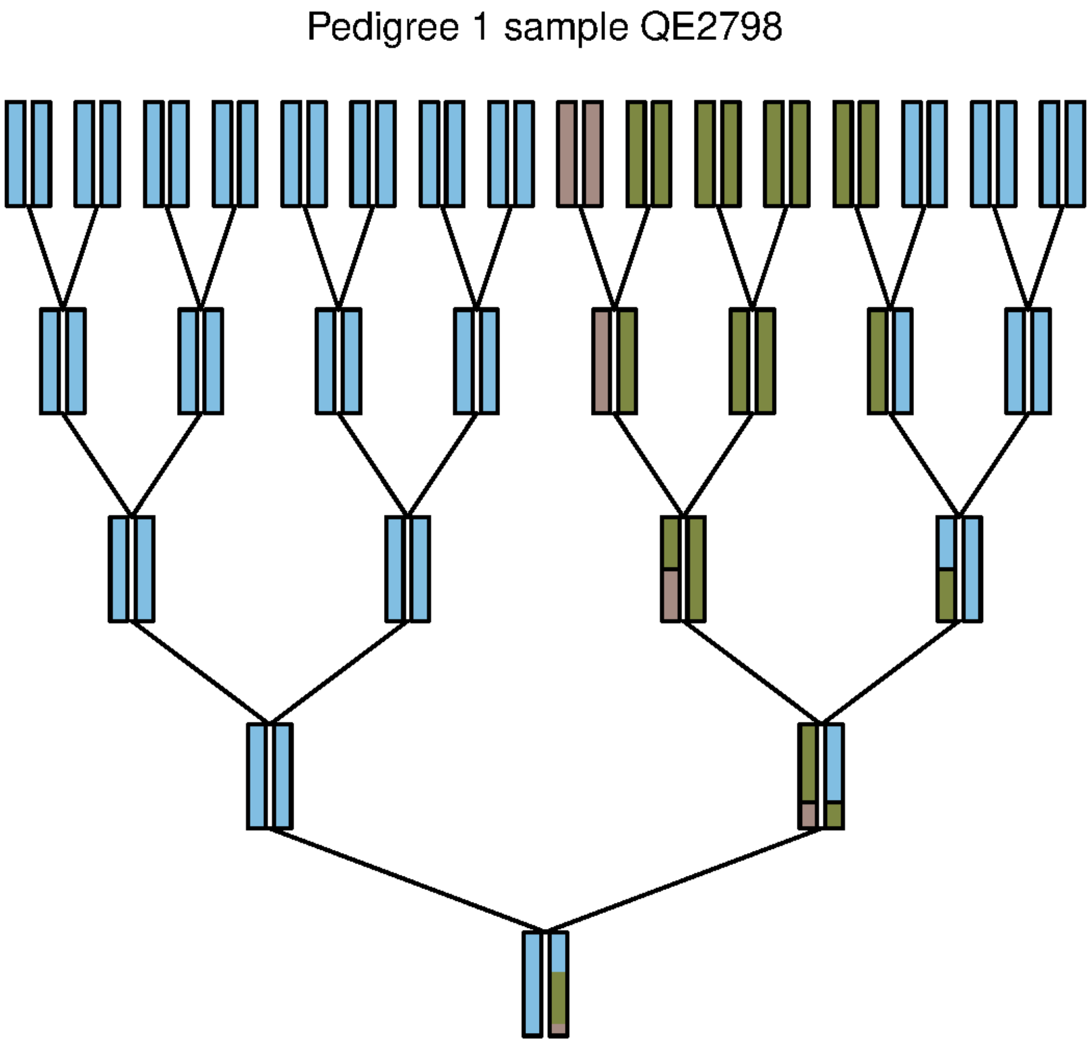

Fig. S11. l)

Pedigree 1 sample QE2801

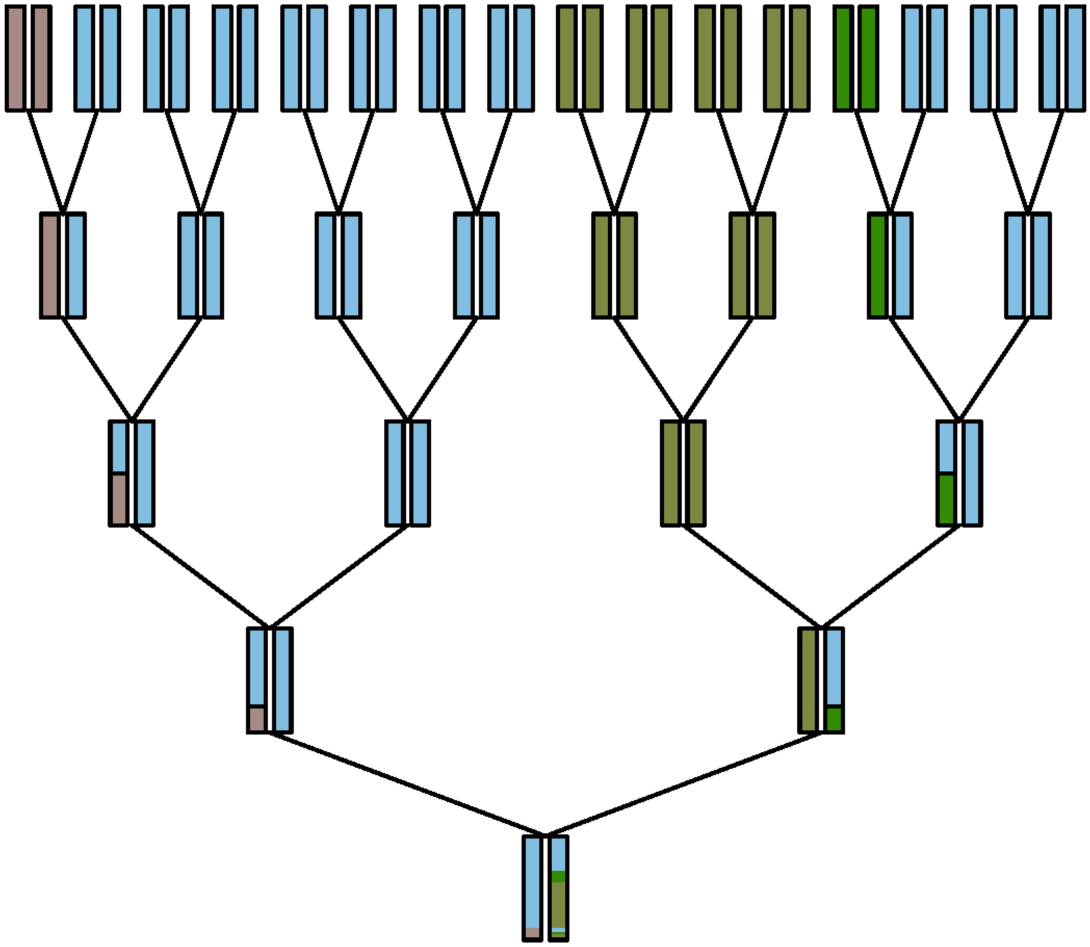

Fig. S11. m)

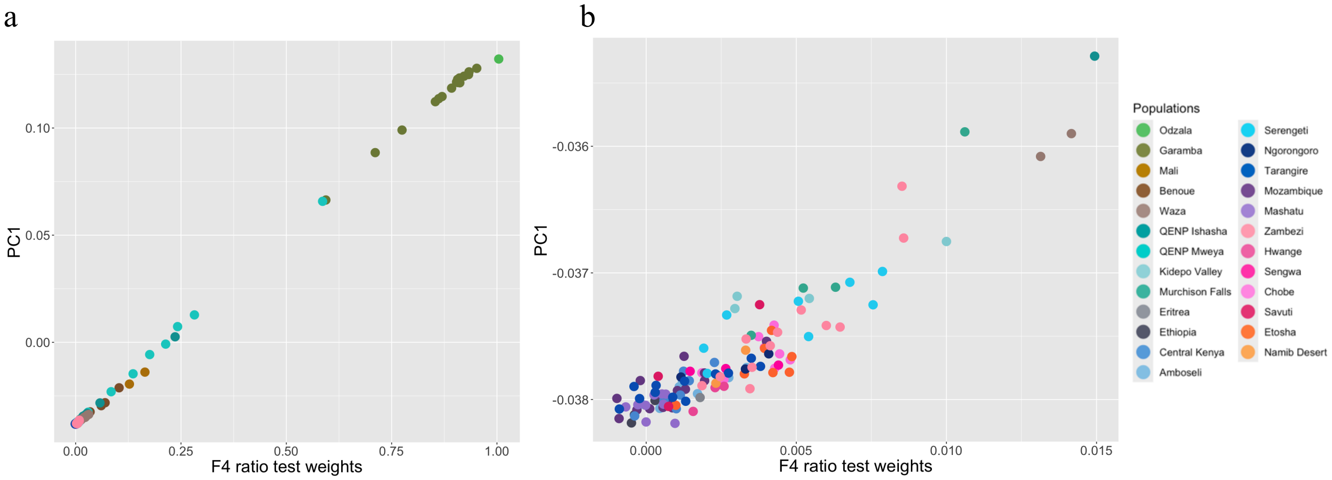

Fig. S12. a) The linear relationship between the F4 ratio values of forest ancestry and the PC1 values for individual elephants. b) An enlarged version to increase resolution.

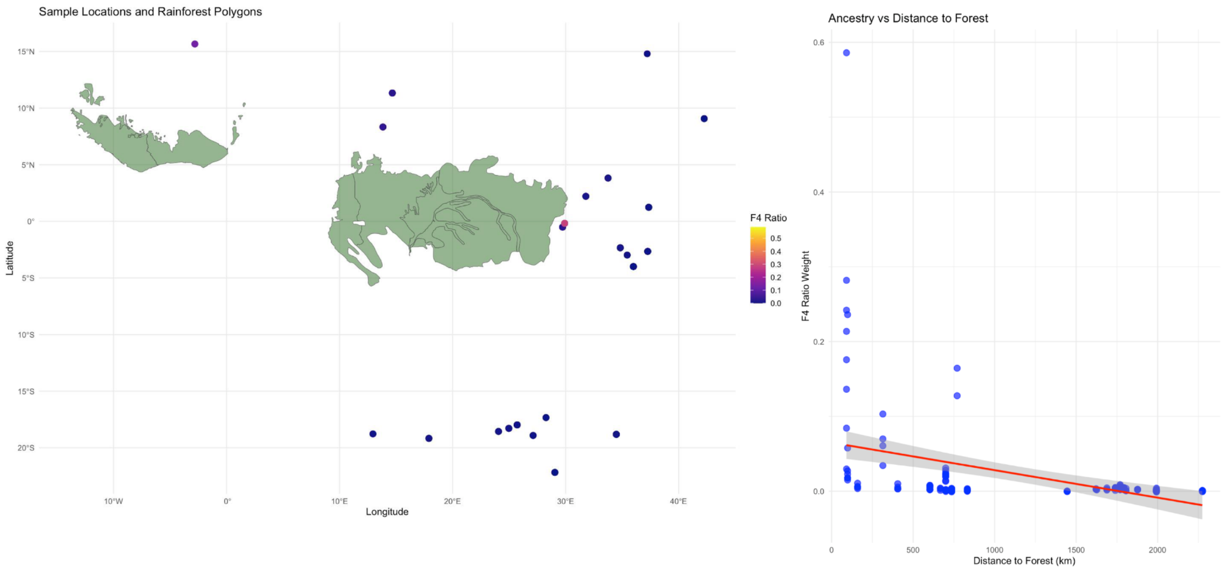

Fig. S13. A Mantel test performed in R v4.4.3[15] in RStudio v2024.12.1.563[18] with the vegan package[19] revealed a weak but significant correlation between forest ancestry and distance to the Congo-Guinean forest (Spearman's ρ = 0.234, p = 1e^{-04}), indicating that forest elephant-associated ancestry tends to decline with increasing distance from the tropical forest. The left panel shows the distribution of forest polygons and the sampled locations, while the right panel shows the correlation between F4 ratio and the distance to the forest in km. The Congo-Guinean forest polygons were extracted from the WWF Terrestrial Ecoregions of the World shapefile[20] and the plots were generate in R v4.4.3[15] in RStudio v2024.12.1.563[18] with packages vegan[19,] ggplot2[16], dplyr[21], sf[22,23], and geosphere[24].

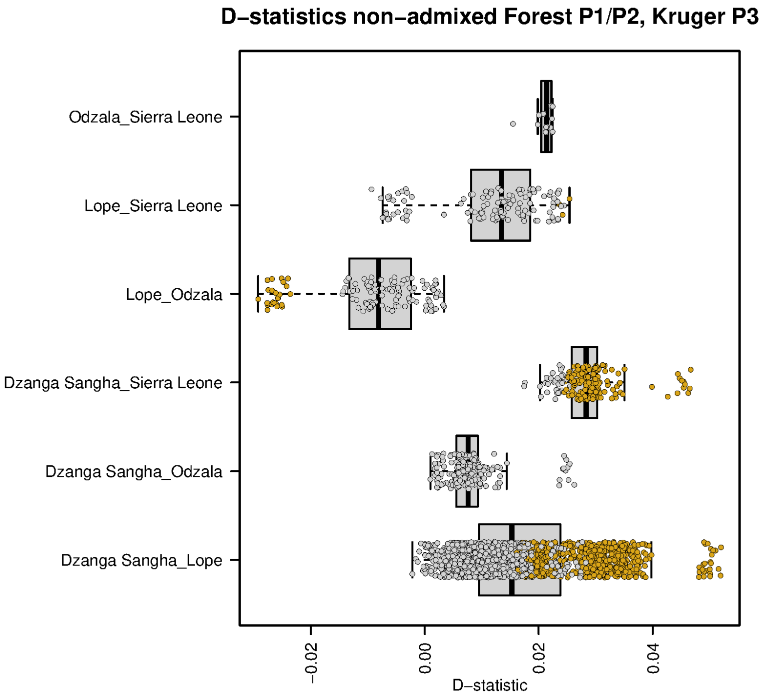

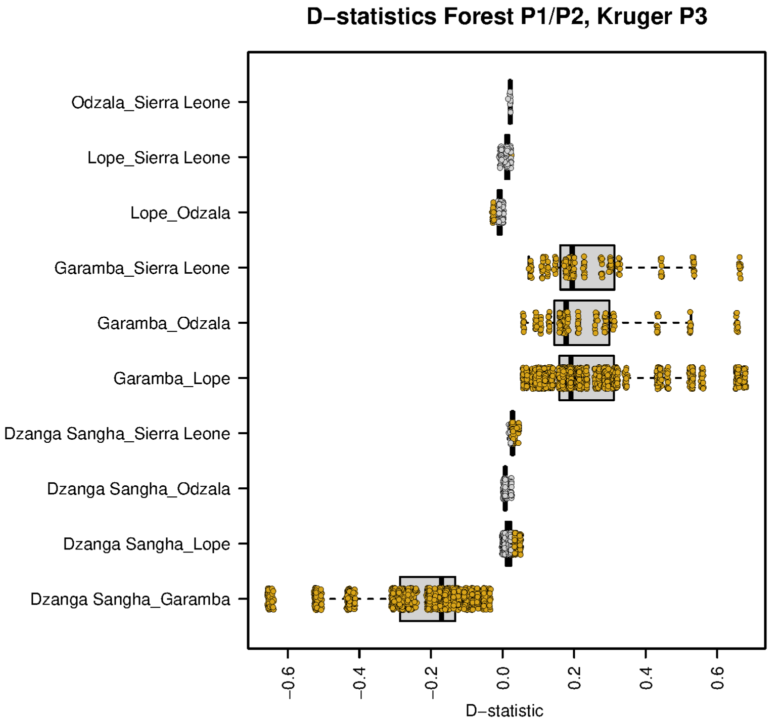

Fig. S14. Analysis of gene flow between species using *D*-statistics[13,14] on the dataset mapped to the Asian elephant. We tested whether any forest elephant population showed an excess of allele sharing with the savanna elephant by placing a relatively unadmixed savanna elephant population (Kruger) as H3 and all combinations of forest elephants in H1 and H2. Note that labels are switched, thus, for example, the third row shows a significant excess of allele sharing in Odzala compared to Lope.

a)

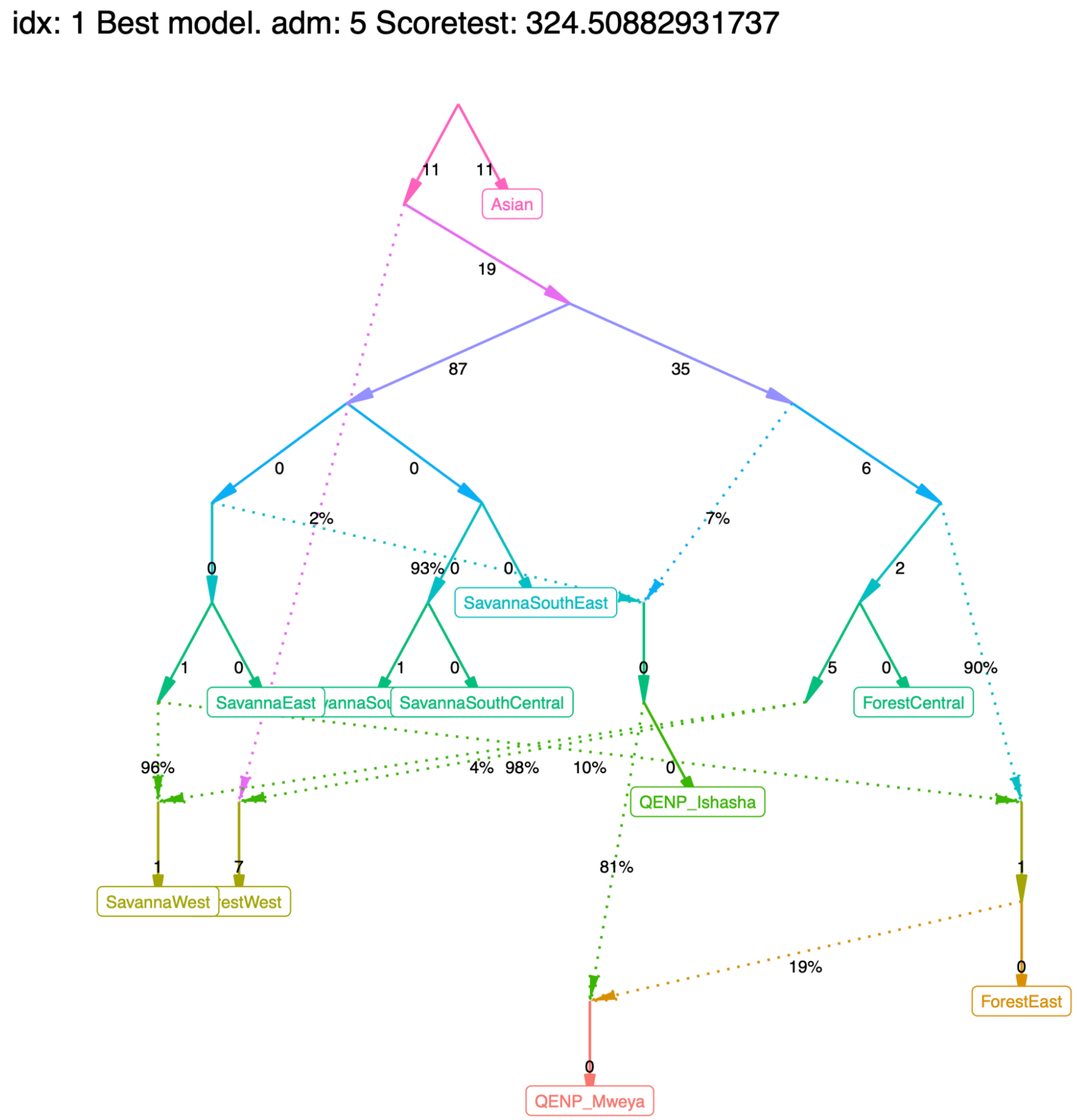

Fig. S15. Genetic relationships in our data estimated with the addition of admixture events in qpGraph performed in ADMIXTOOLS2[25]. For each number of admixture events, we chose the model with the best score. Overall, the model with 5 admixture events had the best score. a) model with 5 admixture events; b) model fit with 5 admixture events; c) model with 4 admixture events; d) model fit with 4 admixture events; e) model with 3 admixture events; f) model fit with 3 admixture events; g) model with 2 admixture events; h) model fit with 2 admixture events; i) model with 1 admixture event; j) model fit with 1 admixture event; k) model with 0 admixture events; l) model fit with 0 admixture events.

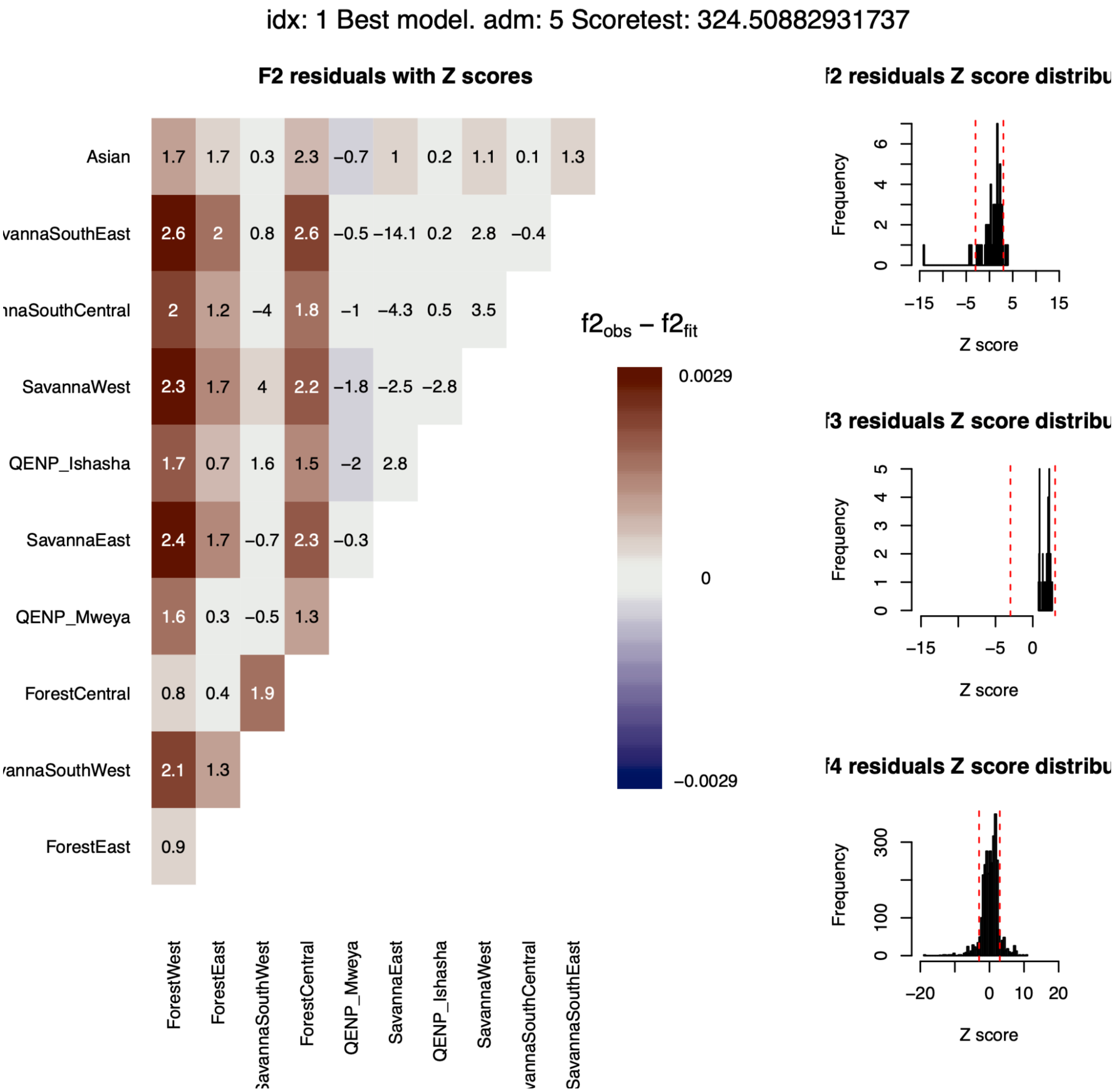
idx: 1 Best model. adm: 5 Scoretest: 324.50882931737
F2 residuals with Z scores
f2 residuals Z score distribu
f3 residuals Z score distribu
f4 residuals Z score distribu
Frequency
Z score
Asian
ForestEast
ForestCentral
QENP_Mweya
SavannaEast
QENP_Ishasha
SavannaWest
ForestWest

Fig. S15. b)

idx: 441 Best model. adm: 4 Scoretest: 436.510136520422

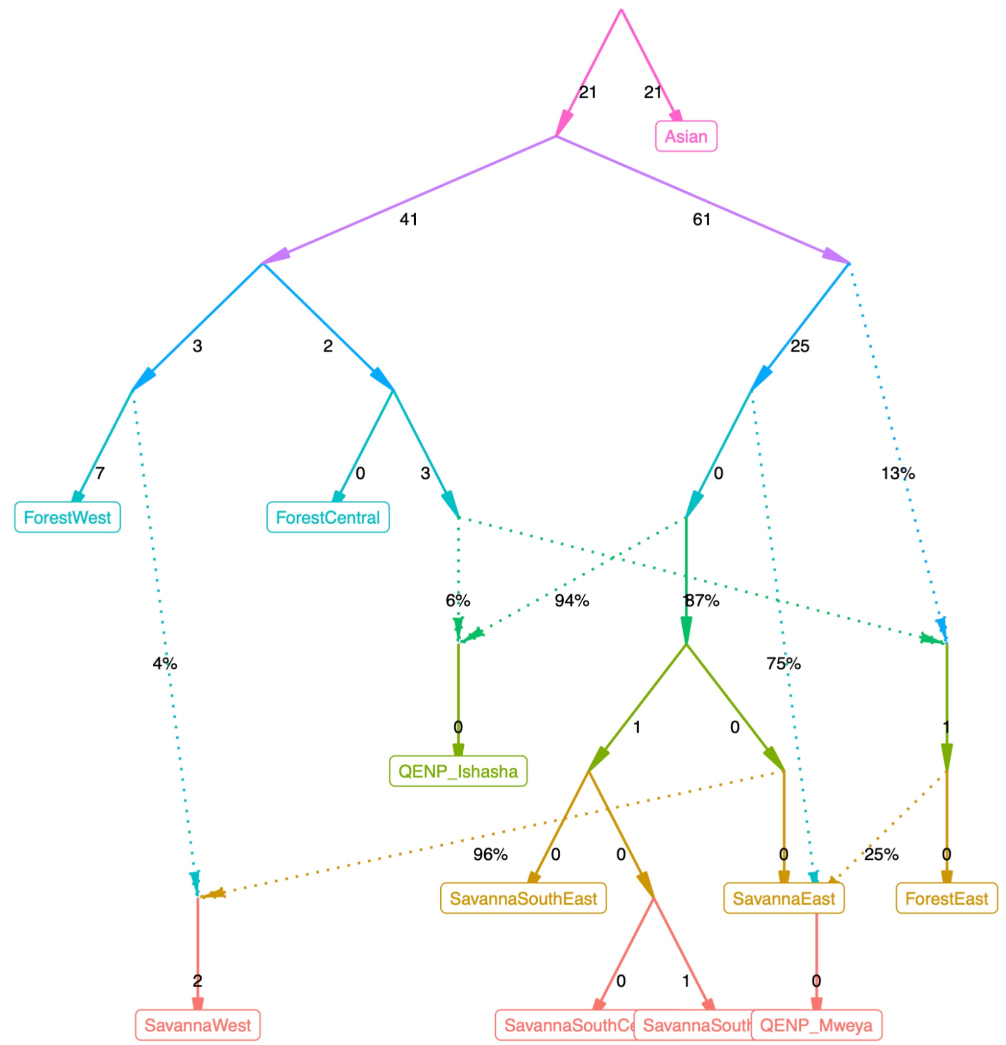

Fig. S15. c)

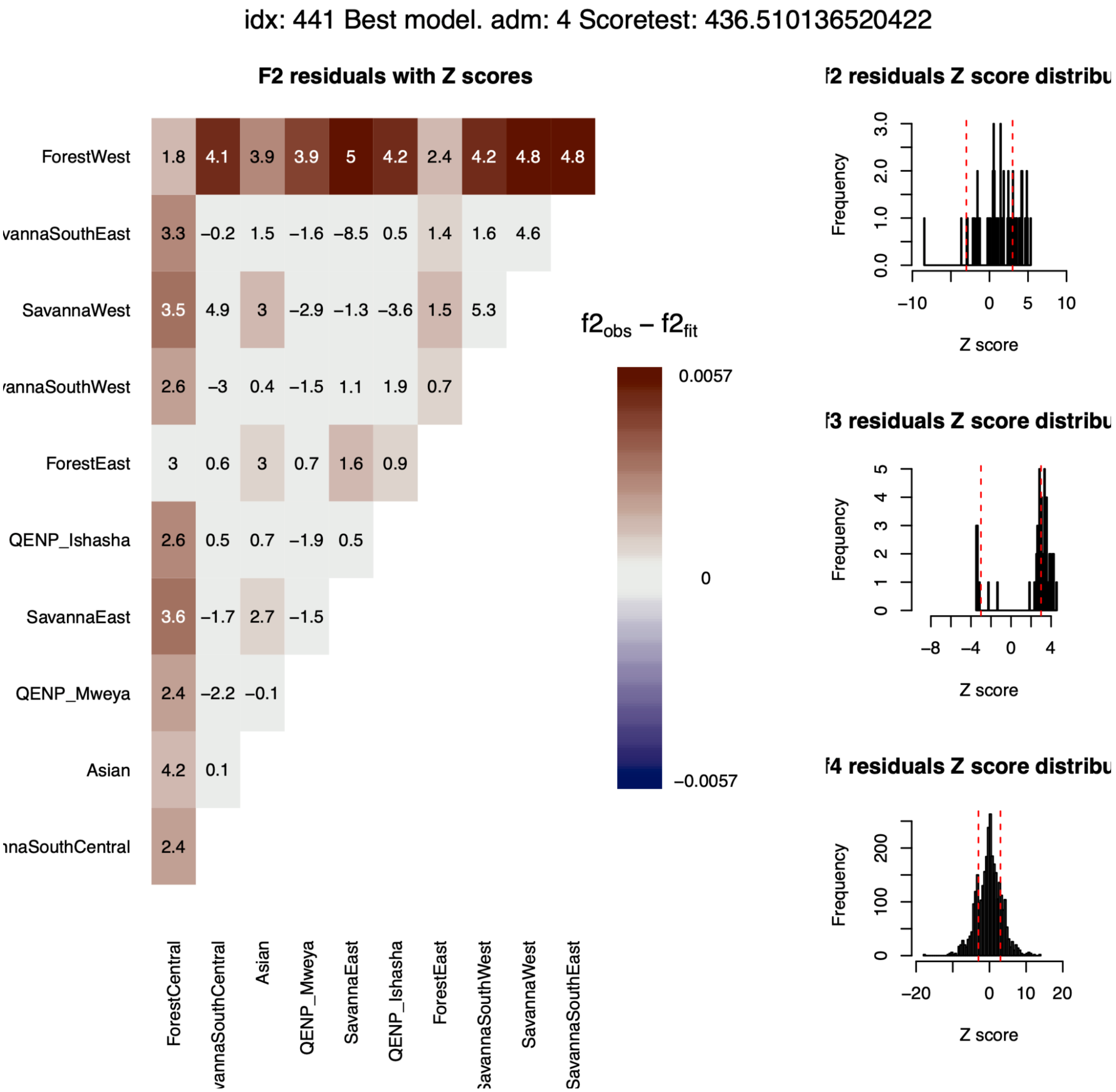

idx: 441 Best model. adm: 4 Scoretest: 436.510136520422
F2 residuals with Z scores
ForestWest
vannaSouthEast
SavannaWest
vannaSouthWest
ForestEast
QENP_Ishasha
SavannaEast
QENP_Mweya
Asian
nnaSouthCentral
1.8 4.1 3.9 3.9 5 4.2 2.4 4.2 4.8 4.8
3.3 −0.2 1.5 −1.6 −8.5 0.5 1.4 1.6 4.6
3.5 4.9 3 −2.9 −1.3 −3.6 1.5 5.3
2.6 −3 0.4 −1.5 1.1 1.9 0.7
3 0.6 3 0.7 1.6 0.9
2.6 0.5 0.7 −1.9 0.5
3.6 −1.7 2.7 −1.5
2.4 −2.2 −0.1
4.2 0.1
2.4
ForestCentral
vannaSouthCentral
Asian
QENP_Mweya
SavannaEast
QENP_Ishasha
ForestEast
SavannaSouthWest
SavannaWest
SavannaSouthEast
f2obs − f2fit
0.0057
0
−0.0057
f2 residuals Z score distribu
f3 residuals Z score distribu
f4 residuals Z score distribu
Frequency
Z score

Fig. S15. d)

idx: 772 Best model. adm: 3 Scoretest: 608.085982201833

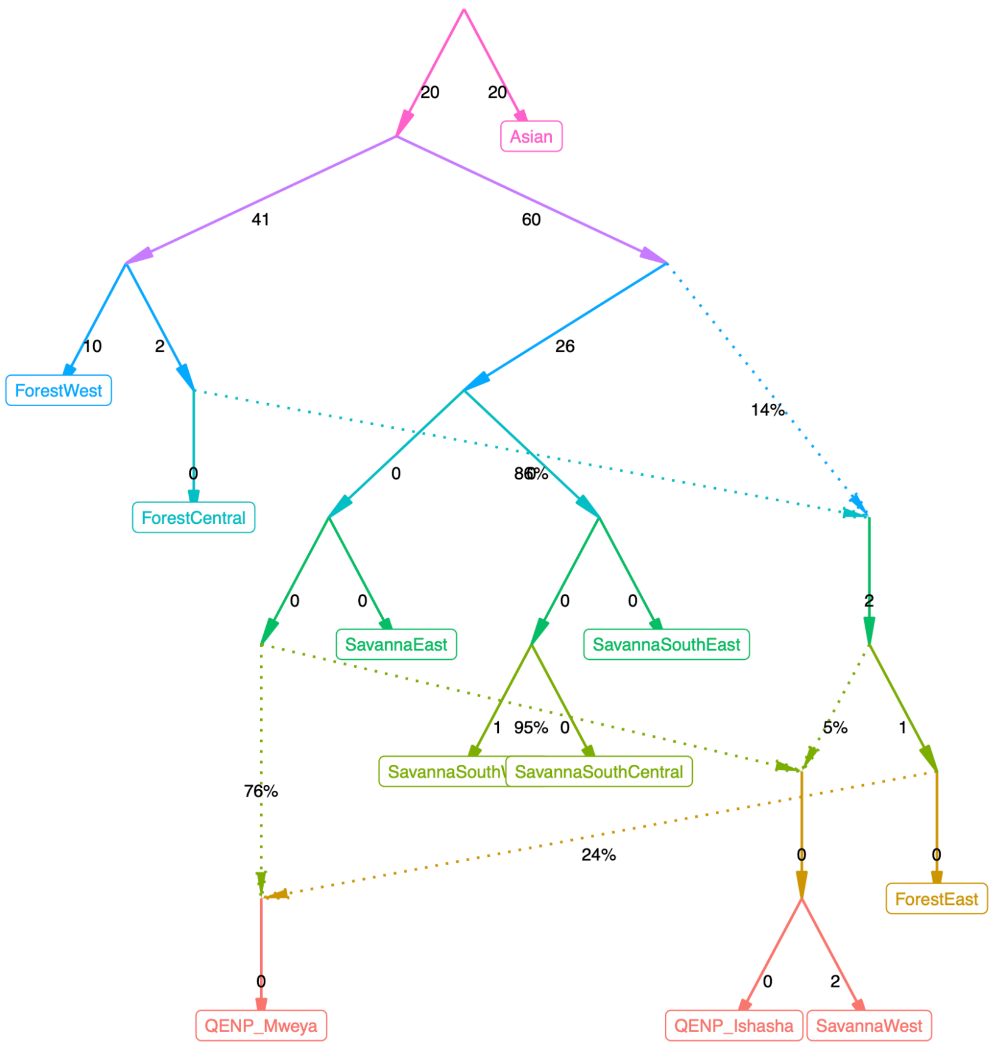

Fig. S15. e)

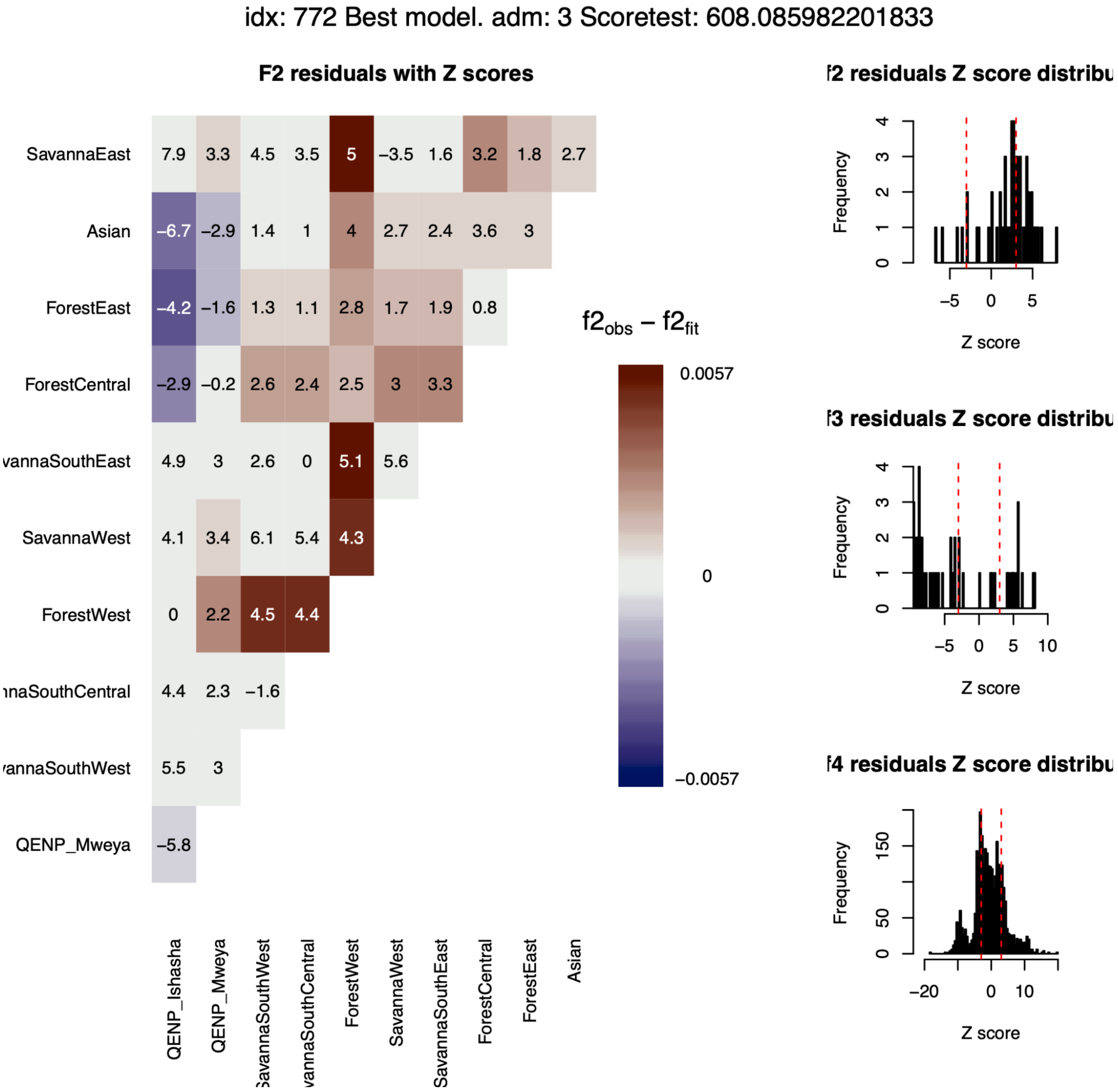
idx: 772 Best model. adm: 3 Scoretest: 608.085982201833
F2 residuals with Z scores
f2 residuals Z score distribu
f3 residuals Z score distribu
f4 residuals Z score distribu
SavannaEast
Asian
ForestEast
ForestCentral
vannaSouthEast
SavannaWest
ForestWest
ınaSouthCentral
/annaSouthWest
QENP_Mweya
QENP_Ishasha
QENP_Mweya
SavannaSouthWest
vannaSouthCentral
ForestWest
SavannaWest
SavannaSouthEast
ForestCentral
ForestEast
Asian
f2obs − f2fit
0.0057
0
−0.0057
Frequency
Z score

Fig. S15. f)

idx: 1047 Best model. adm: 2 Scoretest: 2296.45361754145

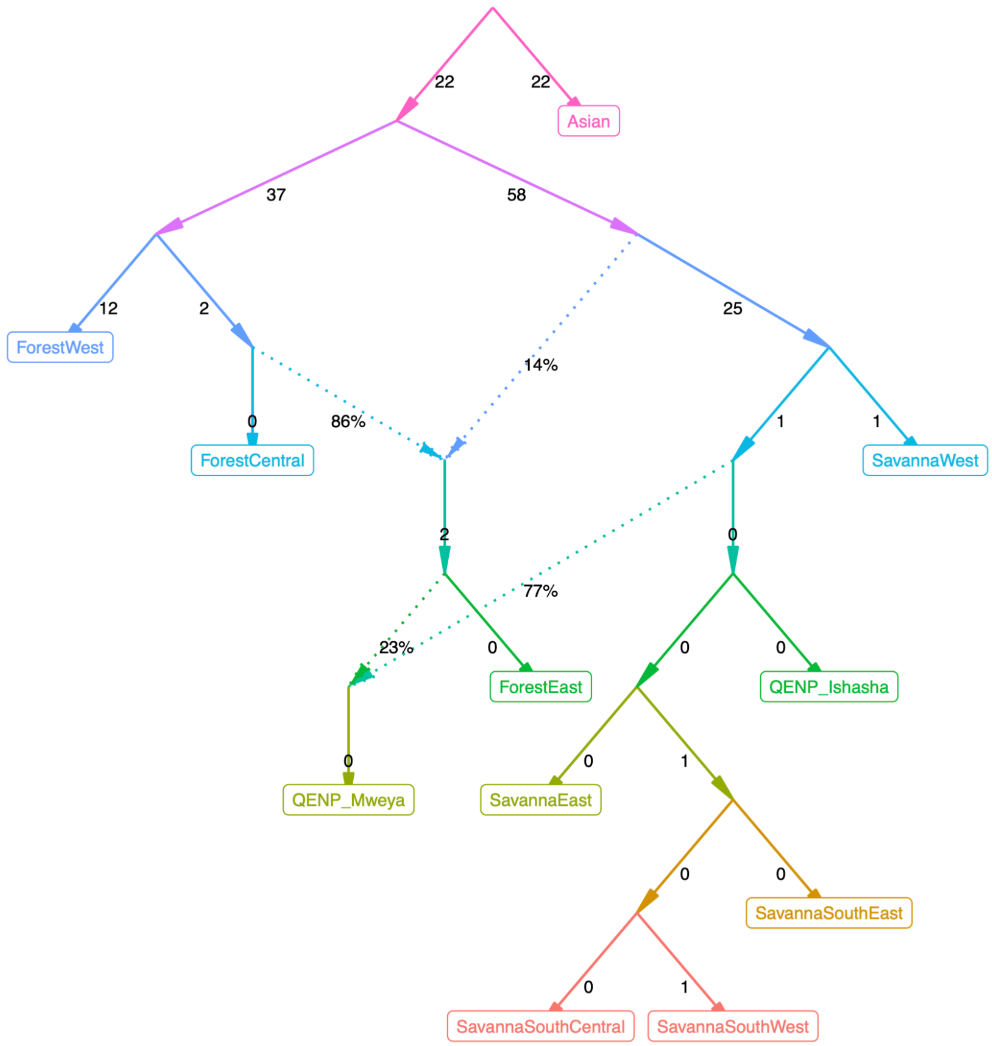

Fig. S15. g)

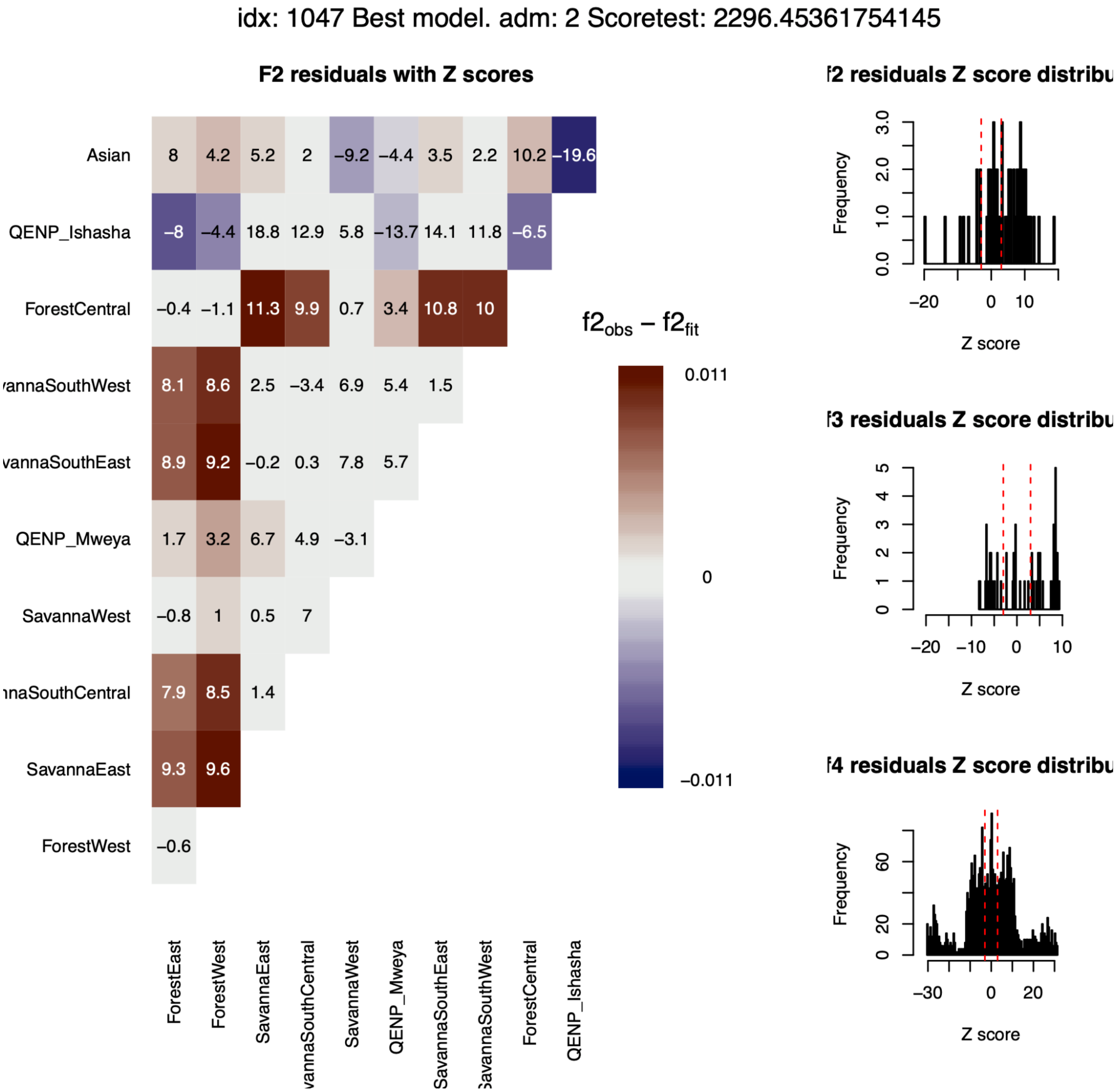
idx: 1047 Best model. adm: 2 Scoretest: 2296.45361754145
F2 residuals with Z scores
Asian
QENP_Ishasha
ForestCentral
ForestWest
SavannaEast
SavannaWest
QENP_Mweya
ForestEast
ForestWest
SavannaEast
SavannaWest
QENP_Mweya
SavannaSouthEast
ForestCentral
QENP_Ishasha
8
4.2
5.2
2
−9.2
−4.4
3.5
2.2
10.2
−19.6
−8
−4.4
18.8
12.9
5.8
−13.7
14.1
11.8
−6.5
−0.4
−1.1
11.3
9.9
0.7
3.4
10.8
10
8.1
8.6
2.5
−3.4
6.9
5.4
1.5
8.9
9.2
−0.2
0.3
7.8
5.7
1.7
3.2
6.7
4.9
−3.1
−0.8
1
0.5
7
7.9
8.5
1.4
9.3
9.6
−0.6
$f2_{obs} - f2_{fit}$
0.011
0
−0.011
Frequency
Z score
0.0
1.0
2.0
3.0
−20
0
10
0
1
2
3
4
5
−20
−10
0
10
0
20
60
−30
0
20

Fig. S15. h)

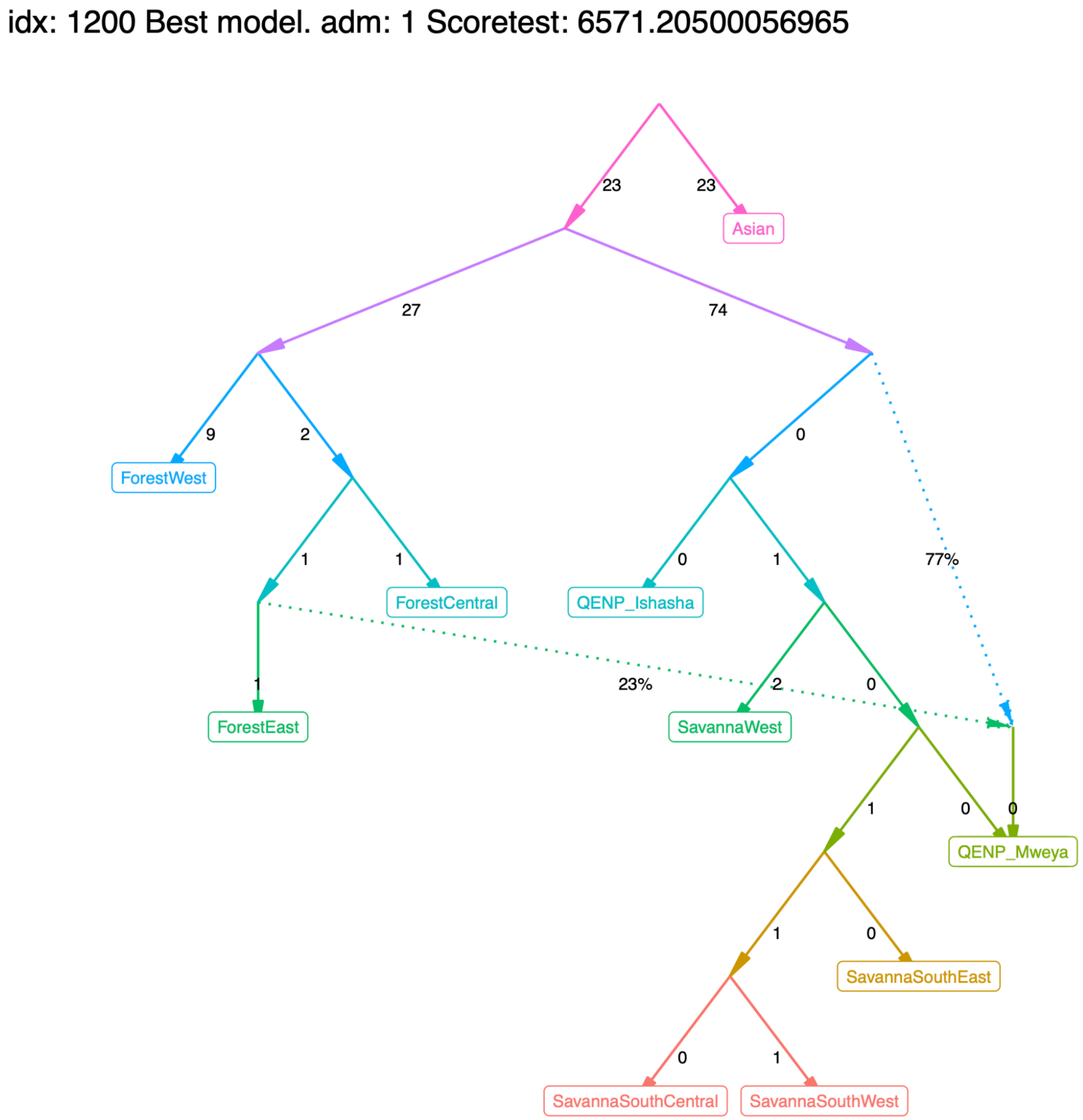
idx: 1200 Best model. adm: 1 Scoretest: 6571.20500056965
23
23
Asian
27
74
9
2
ForestWest
0
1
1
ForestCentral
0
1
QENP_Ishasha
77%
1
ForestEast
23%
2
SavannaWest
0
1
0
0
QENP_Mweya
1
0
SavannaSouthEast
0
1
SavannaSouthCentral
SavannaSouthWest

Fig. S15. i)

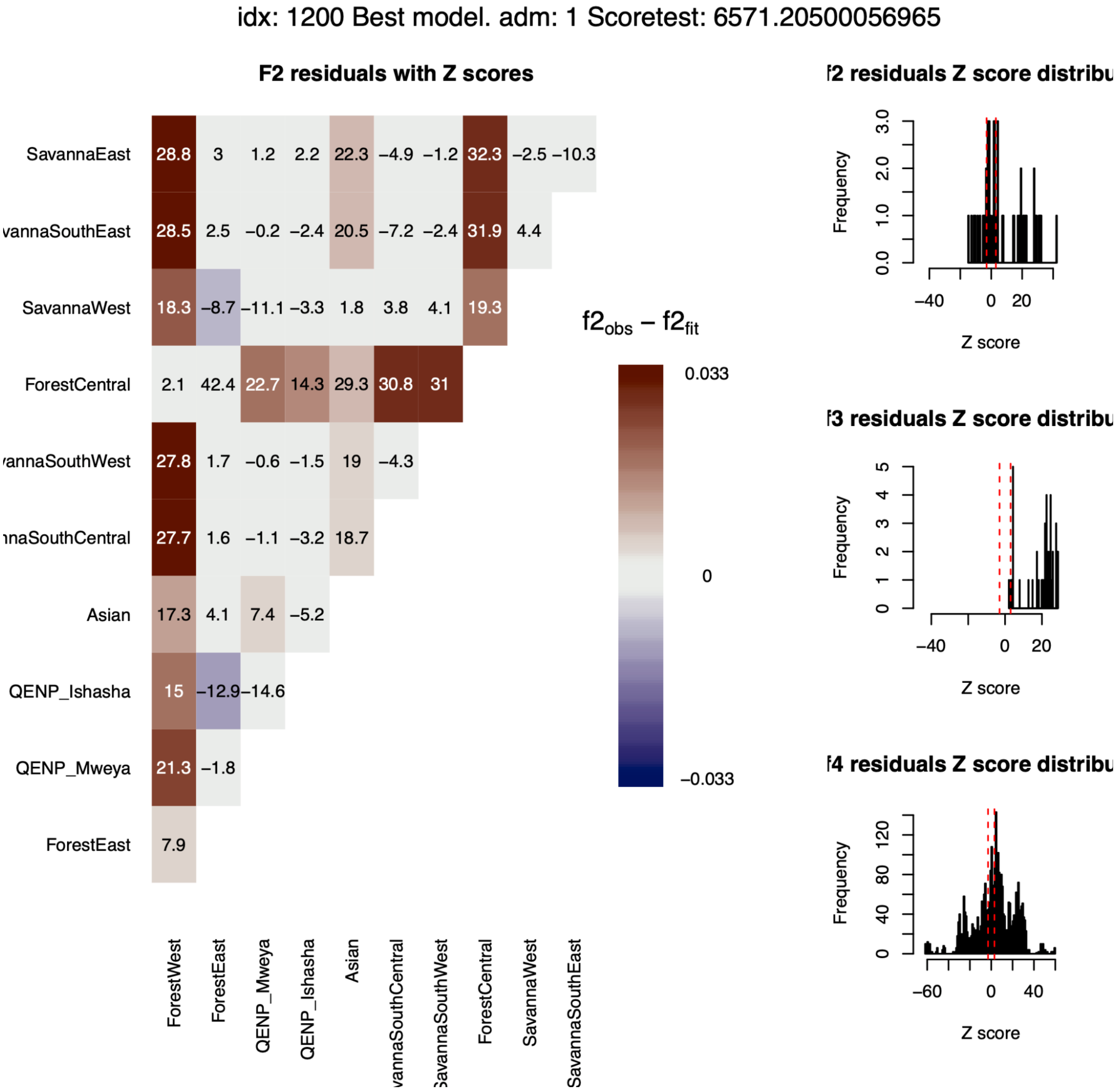
idx: 1200 Best model. adm: 1 Scoretest: 6571.20500056965
F2 residuals with Z scores
f2 residuals Z score distribu
f3 residuals Z score distribu
f4 residuals Z score distribu
Frequency
Z score
SavannaEast
ForestCentral
Asian
QENP_Ishasha
QENP_Mweya
ForestEast
ForestWest
SavannaWest

Fig. S15. j)

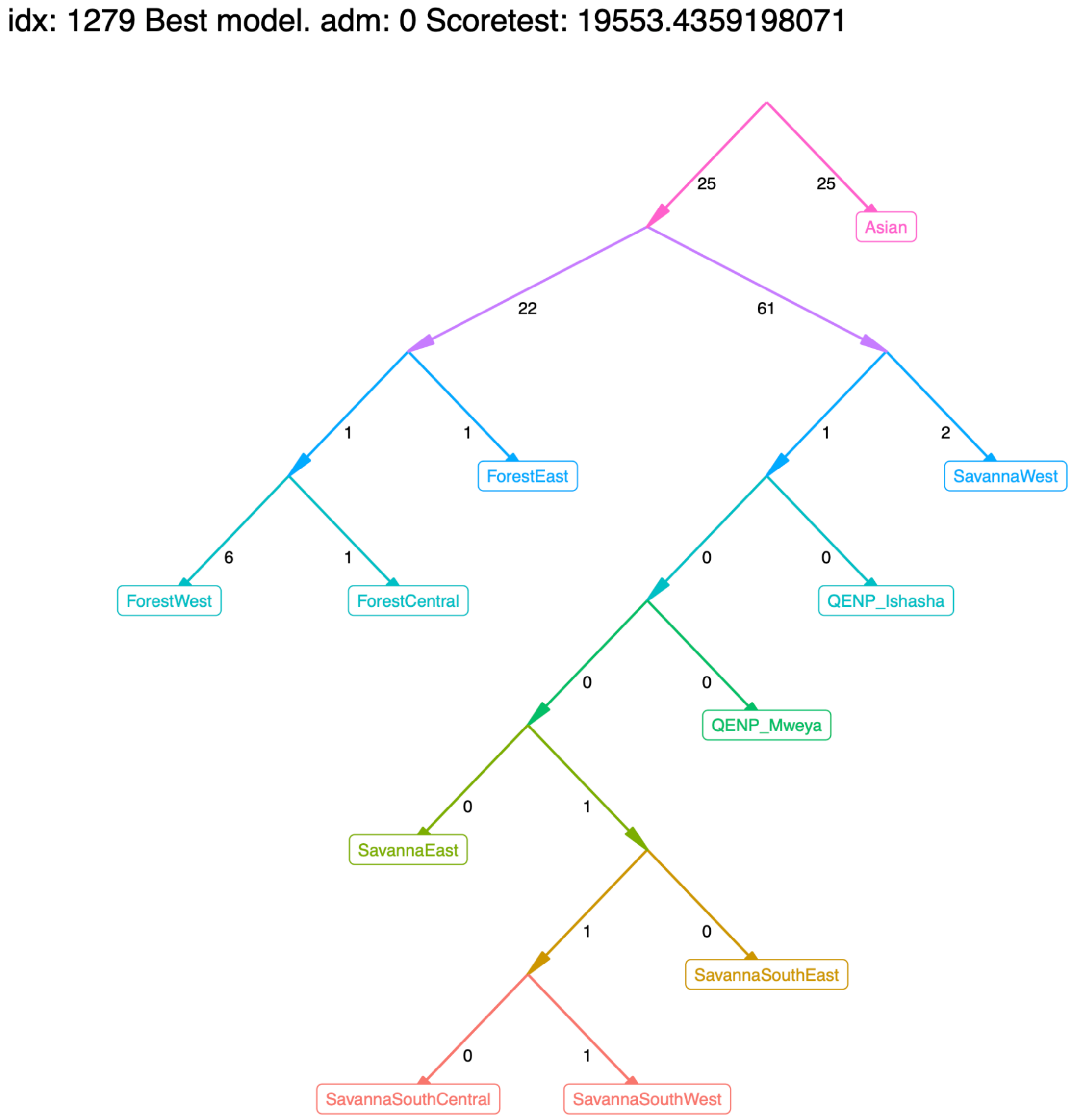
idx: 1279 Best model. adm: 0 Scoretest: 19553.4359198071
25
25
Asian
22
61
1
1
ForestEast
1
2
SavannaWest
6
1
ForestWest
ForestCentral
0
0
QENP_Ishasha
0
0
QENP_Mweya
0
1
SavannaEast
1
0
SavannaSouthEast
0
1
SavannaSouthCentral
SavannaSouthWest

Fig. S15. k)

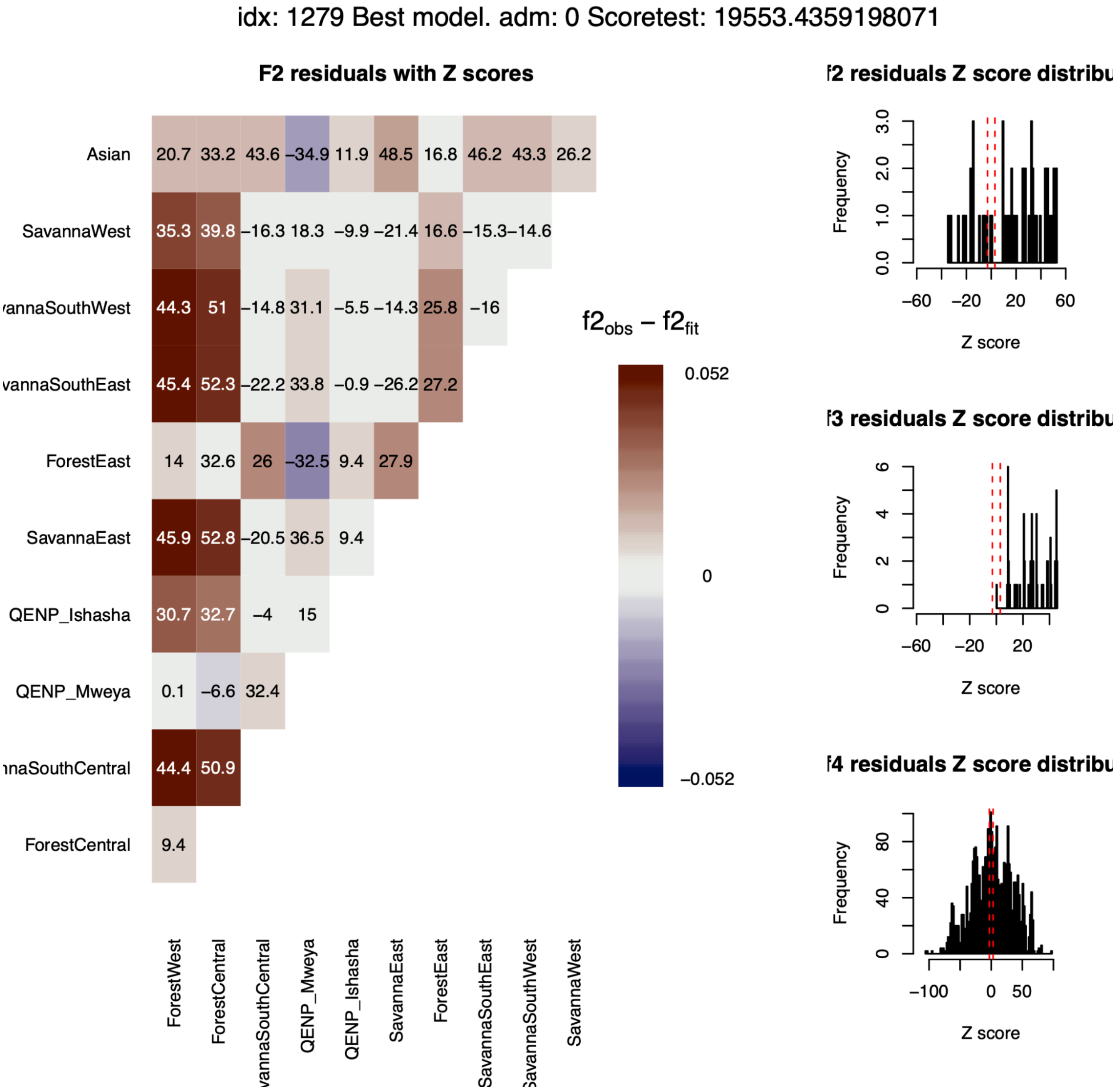
idx: 1279 Best model. adm: 0 Scoretest: 19553.4359198071
F2 residuals with Z scores
Asian
SavannaWest
ForestEast
SavannaEast
QENP_Ishasha
QENP_Mweya
ForestCentral
ForestWest
QENP_Mweya
QENP_Ishasha
SavannaEast
ForestEast
SavannaWest
f2obs − f2fit
0.052
0
−0.052
Frequency
Z score

Fig. S15. l)

a)

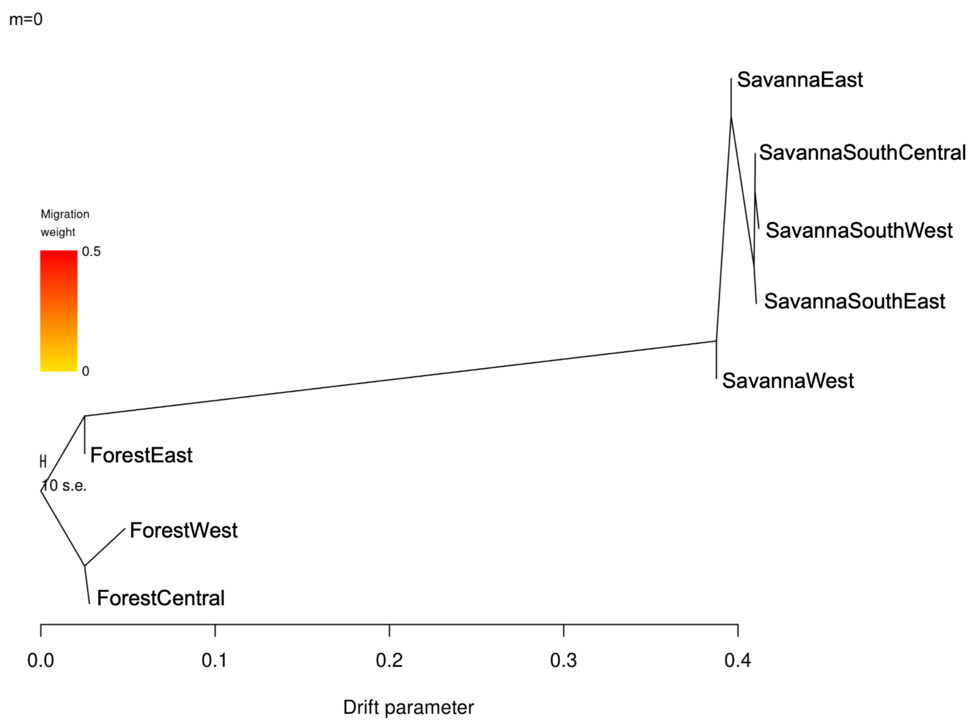

Fig. S16. Population splits and migration events using the imputed data for 224 elephant samples in TreeMix v1.13[26]. a) with 0 migration edges; b) with 1 migration edge; c) with 2 migration edges; d) with 3 migration edges; e) with 4 migration edges; f) with 5 migration edges; g) with 6 migration edges; h) with 7 migration edges.

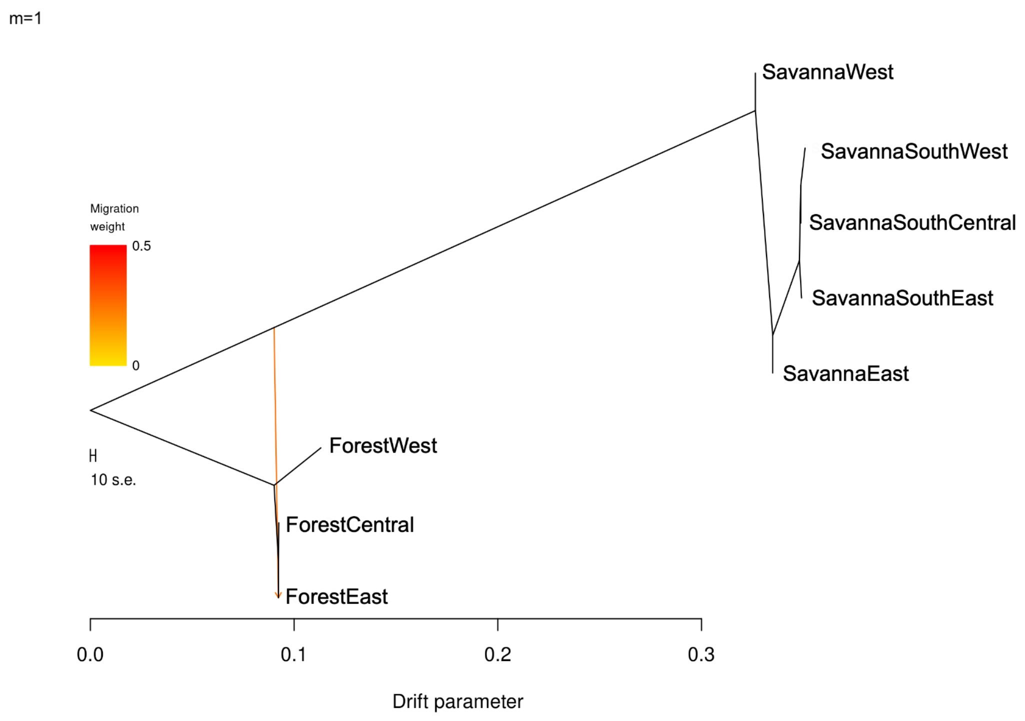

Fig. S16. b)

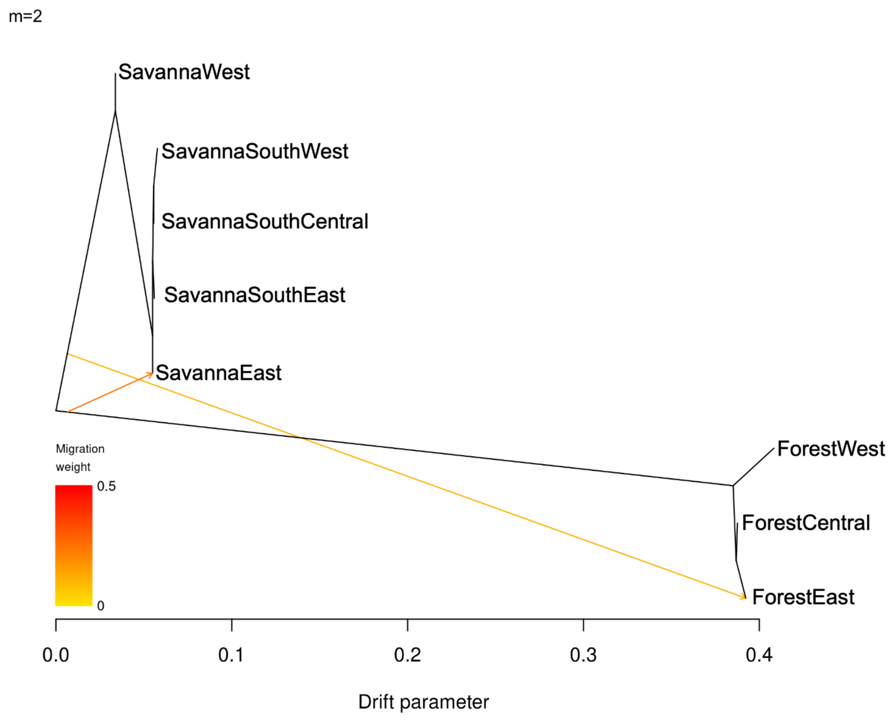

Fig. S16. c)

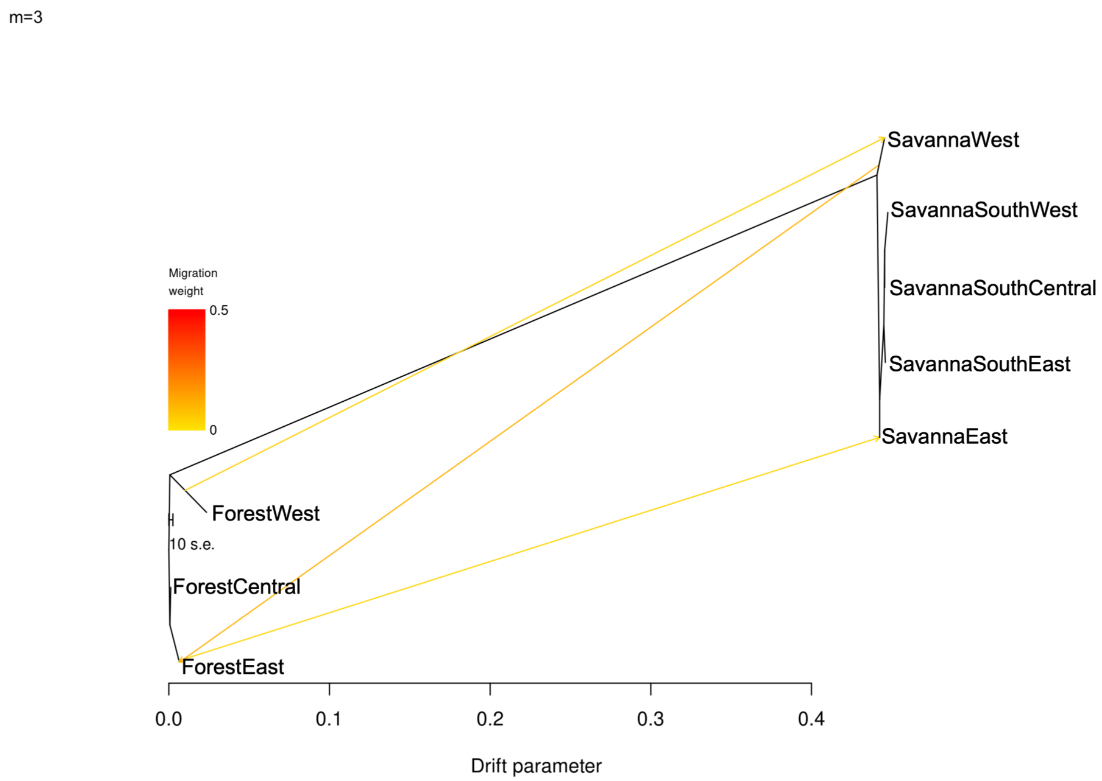

Fig. S16. d)

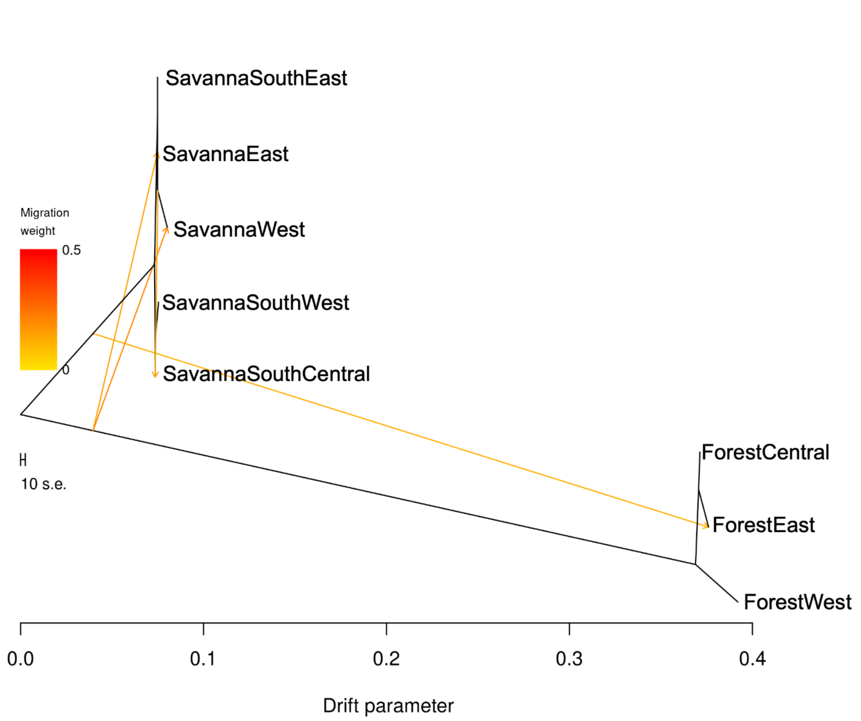

Fig. S16. e)

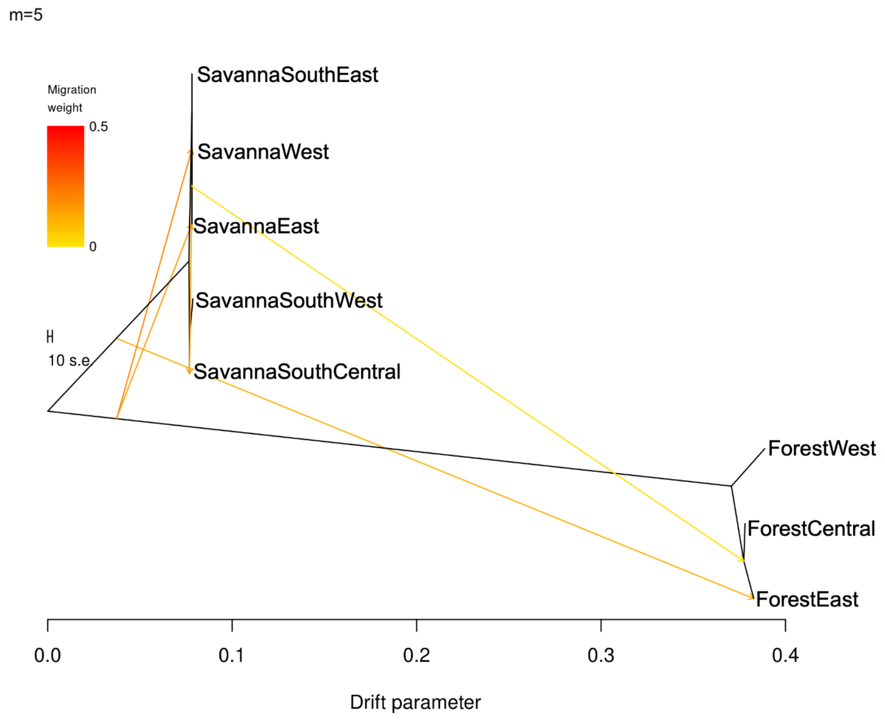

Fig. S16. f)

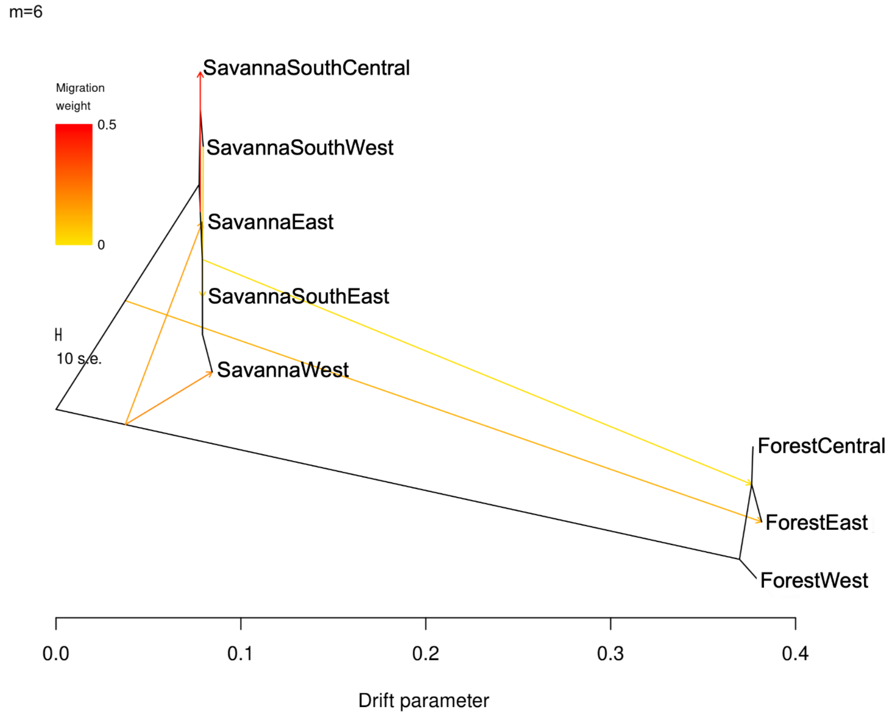

Fig. S16. g)

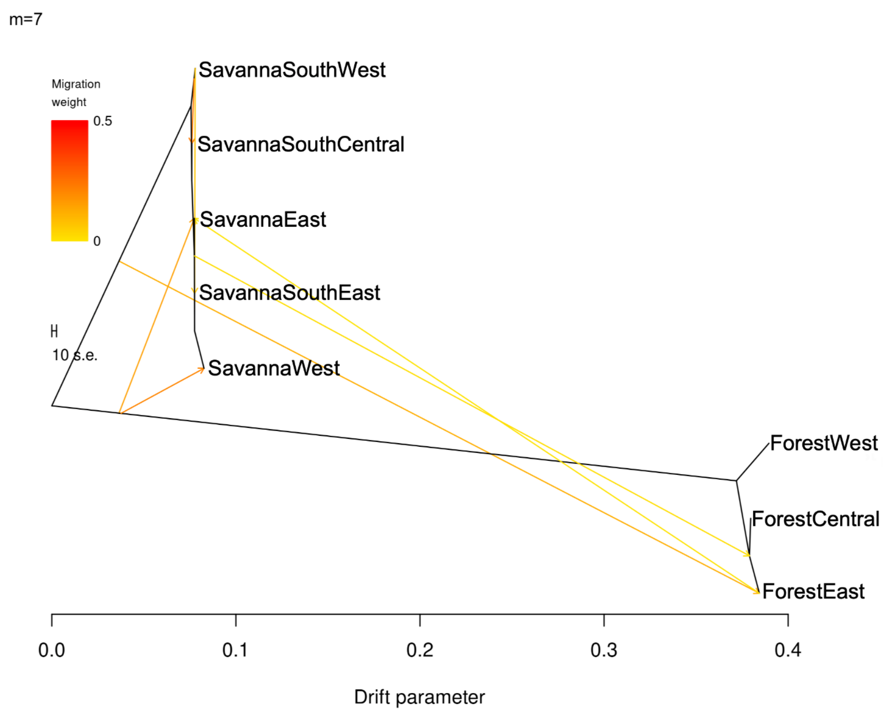
m=7
Migration weight
0.5
0
10 s.e.
SavannaSouthWest
SavannaSouthCentral
SavannaEast
SavannaSouthEast
SavannaWest
ForestWest
ForestCentral
ForestEast
0.0
0.1
0.2
0.3
0.4
Drift parameter

Fig. S16. h)

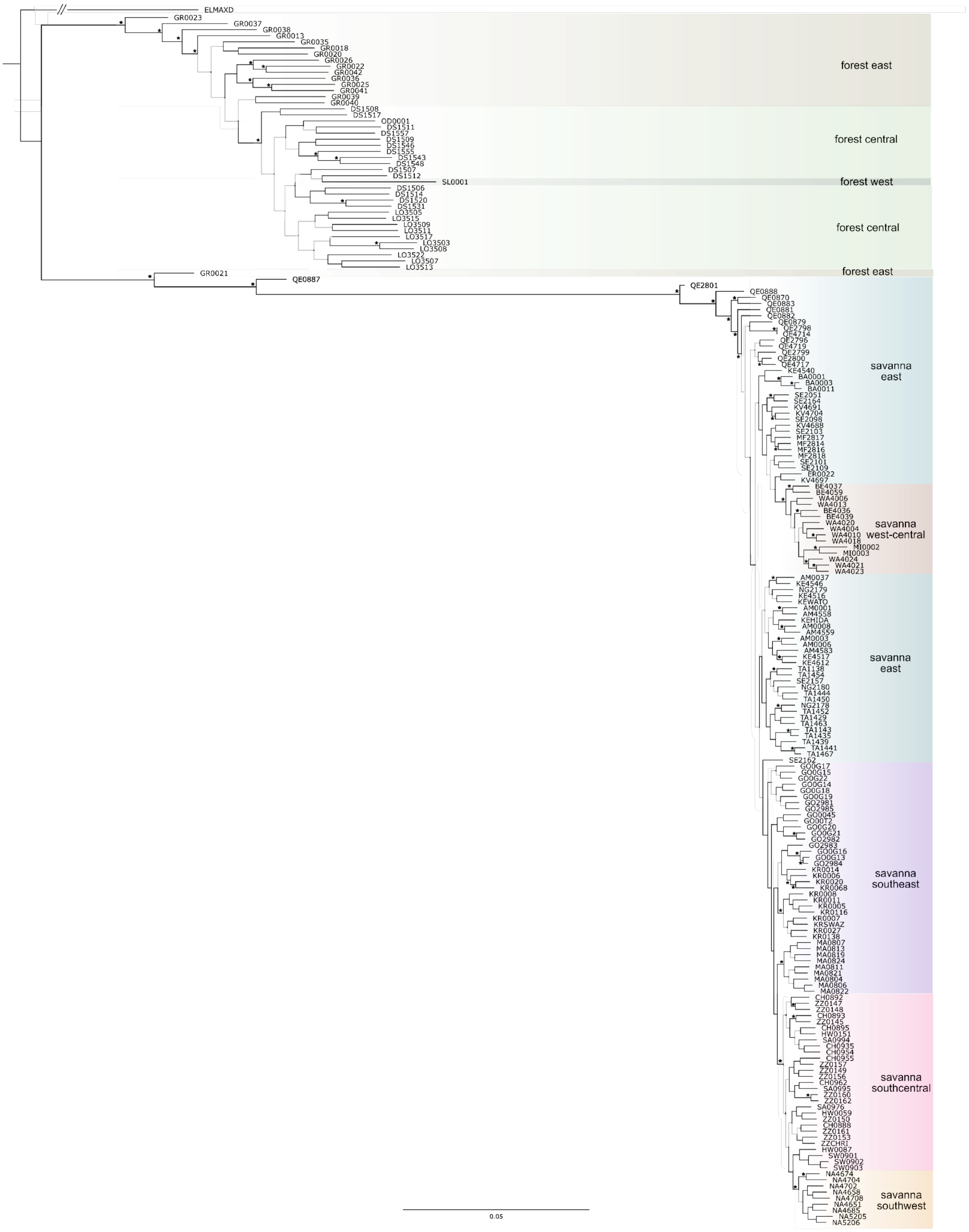

Fig. S17. A phylogenetic tree of African forest and savanna elephants inferred in IQ-TREE[27,28]. Light branches indicate bootstrap support below 0.75, asterisks indicate branches with support of at least 0.95. The outgroup branch of the Asian elephant was trimmed to increase visibility.

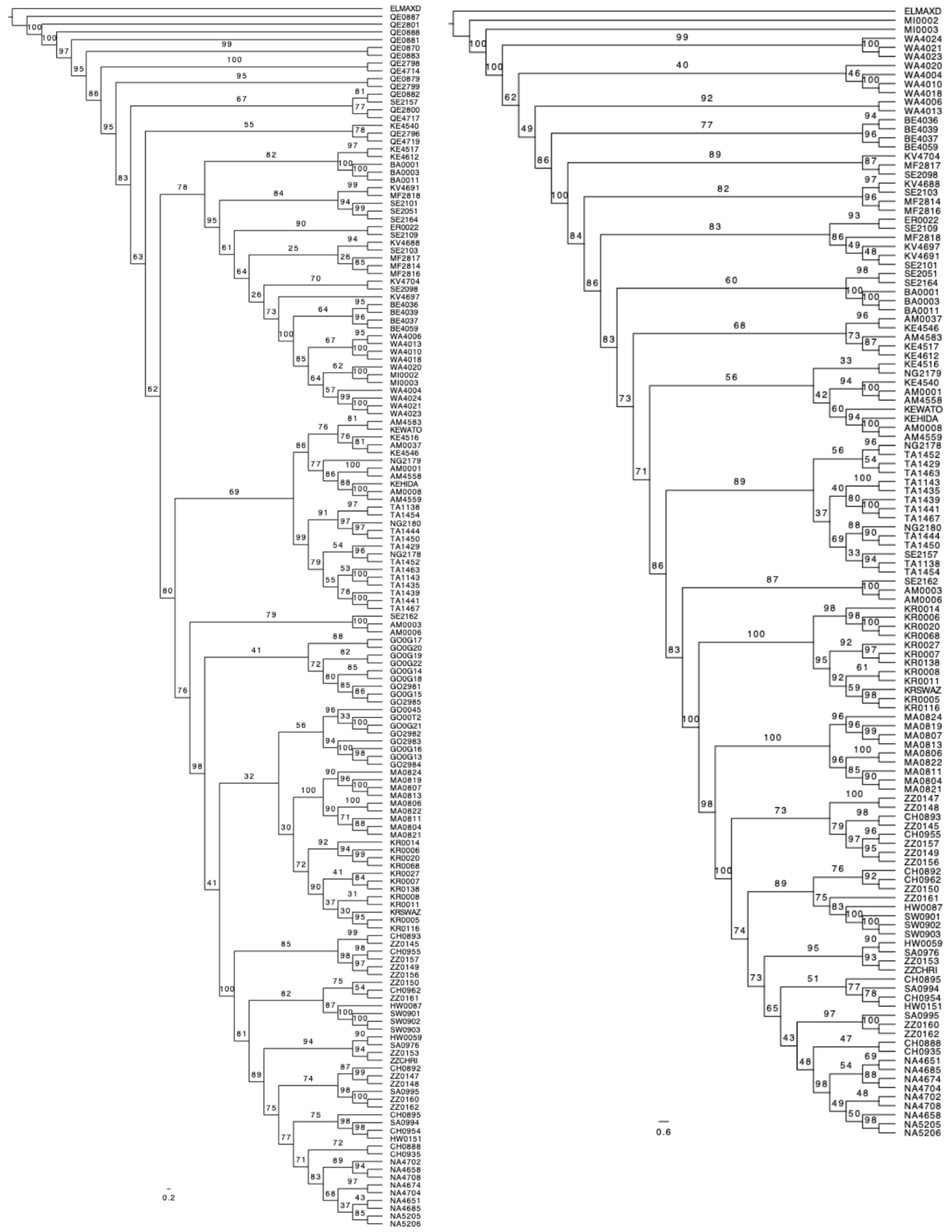

Fig. S18. Phylogenetic trees of savanna elephants inferred in IQ-TREE[27,28]. Since the branches are short, the trees are shown as cladograms to increase visibility. The left tree includes the hybrid populations from the Queen Elizabeth National Park (QE) and the right tree excludes these, directly affecting position of the west-central savanna elephants (WA, BE, MI).

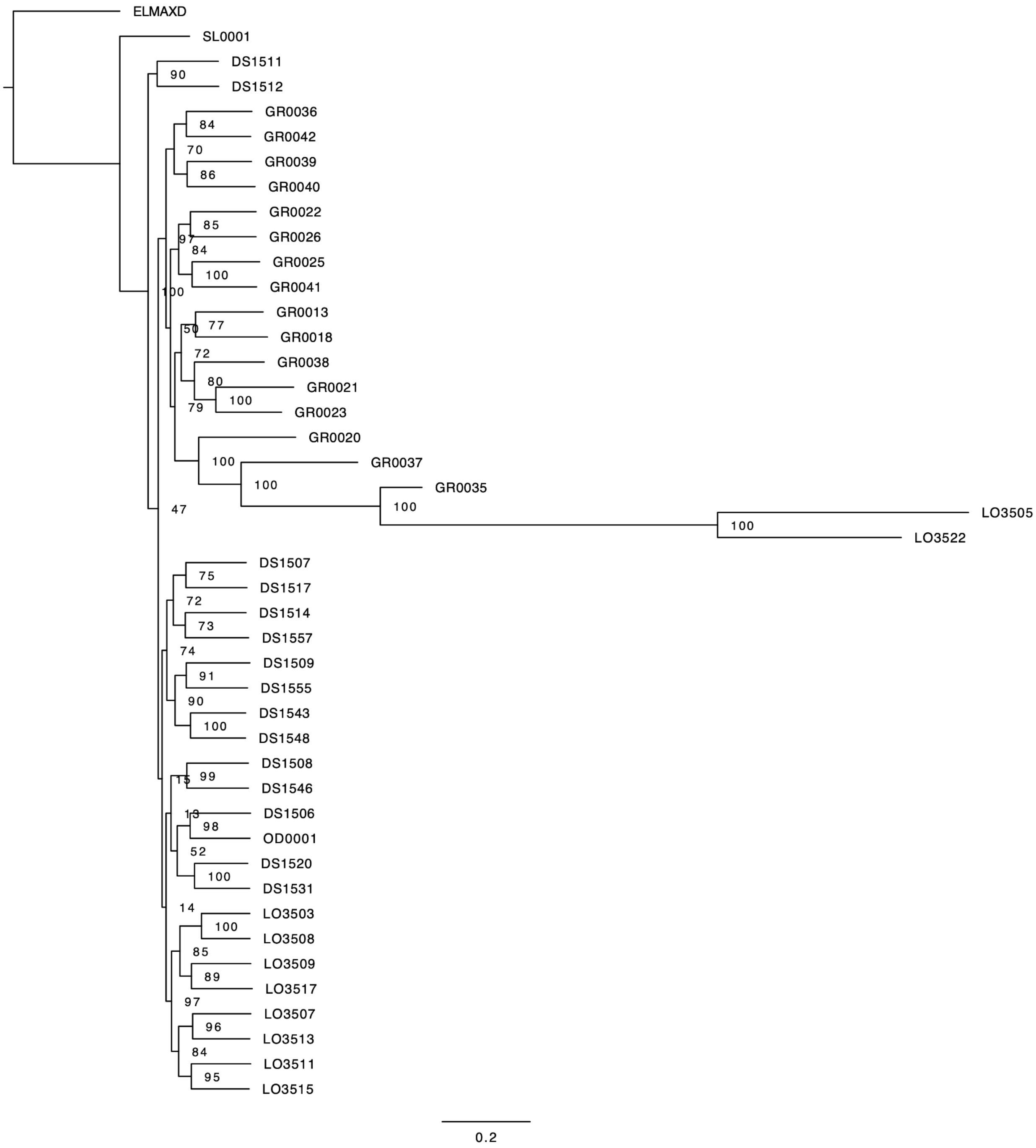

Fig. S19. A phylogenetic tree of forest elephants inferred in IQ-TREE[22,23].

a

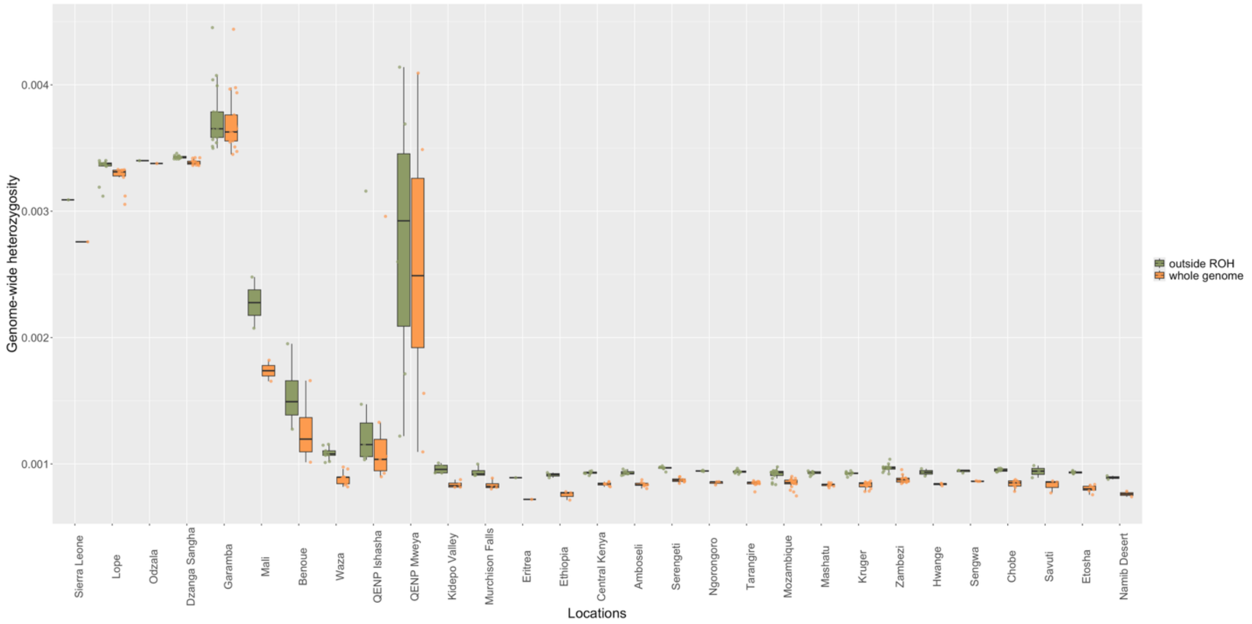

b

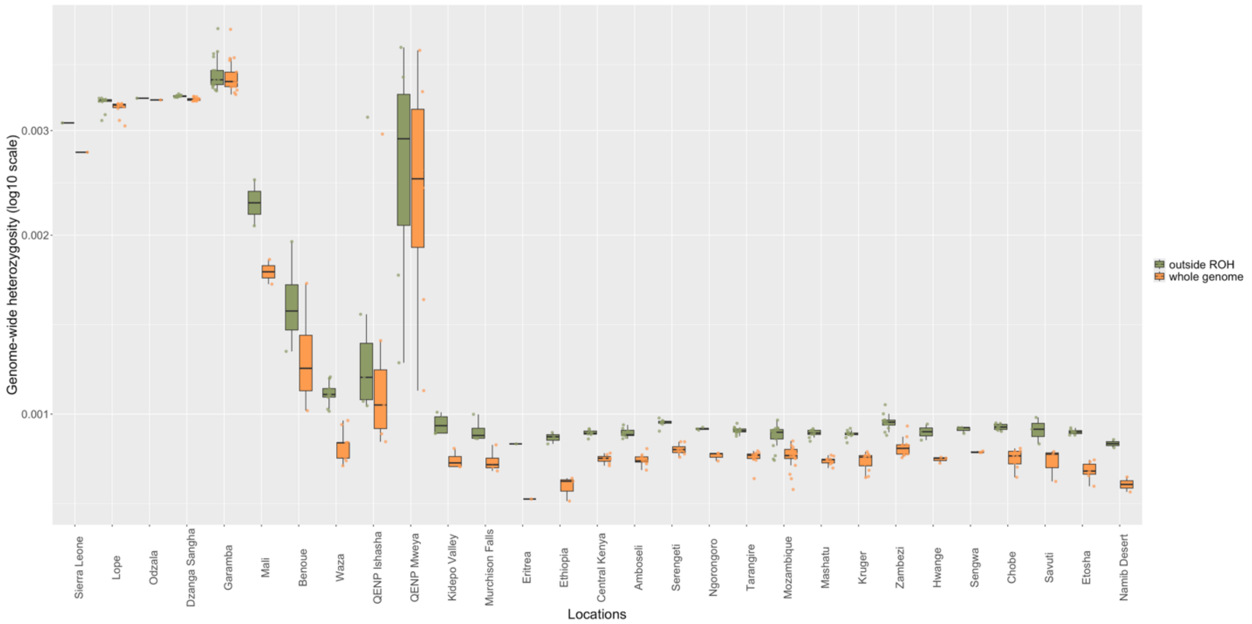

Fig. S20. a) Genome-wide heterozygosity including and excluding the runs of homozygosity (ROH). b) The same plotted in log10 scale. Boxplots show the median (central line) and 25th and 75th percentiles (box), whiskers extend to the minimum and maximum values within 1.5-times the interquartile range.

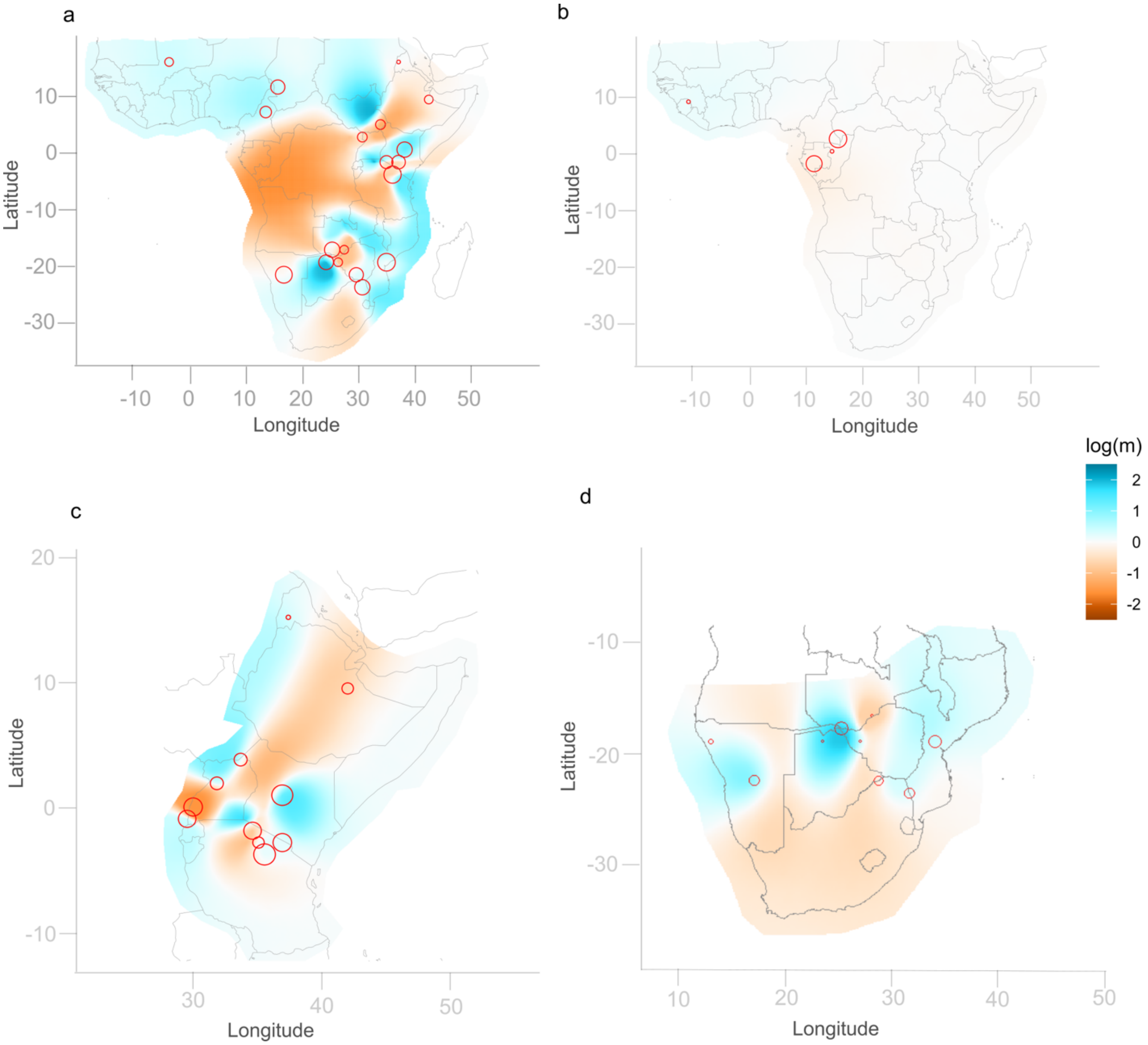

Fig. S21. Estimation of Effective Migration Surfaces (EEMS[29]) assessing connectivity between populations of each species. The plots were generated in R following the EEMS plotting pipeline[29]. The base maps were generated using data from Natural Earth (public domain) using the rworldmap package[30]. The EEMS analysis reveals the areas of relative genetic connectivity (blue) and barriers to gene flow (orange). a) All savanna elephants without the QENP (Ishasha, Mweya) hybrid populations. b) All forest elephants without the hybrid Garamba population. c) Eastern African savanna elephants (including QENP). d) Southern African savanna elephants.

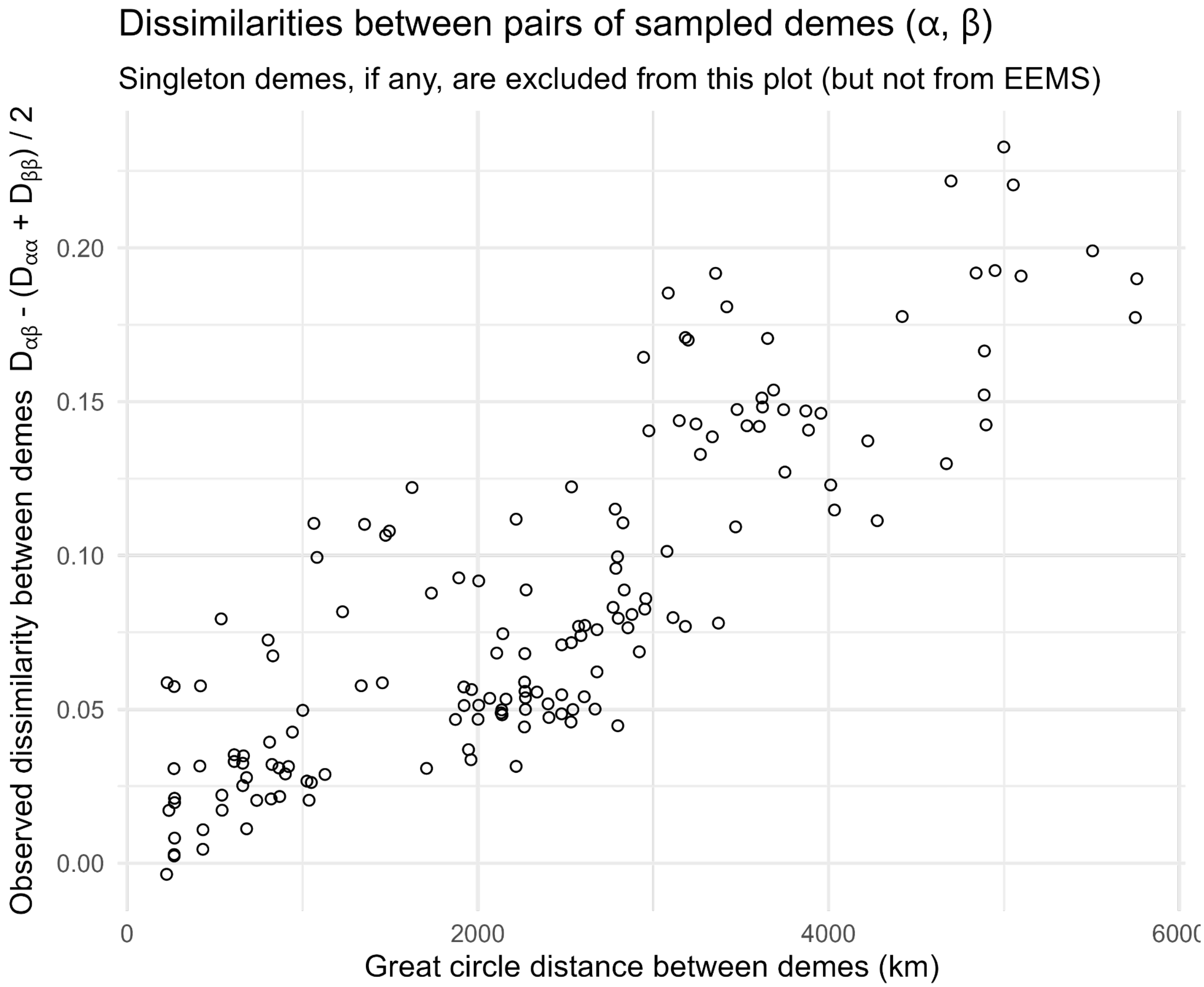

Fig. S22. Isolation-by-distance-like pattern observed when plotting genetic distance against geographic distance, as outputted by EEMS[29].

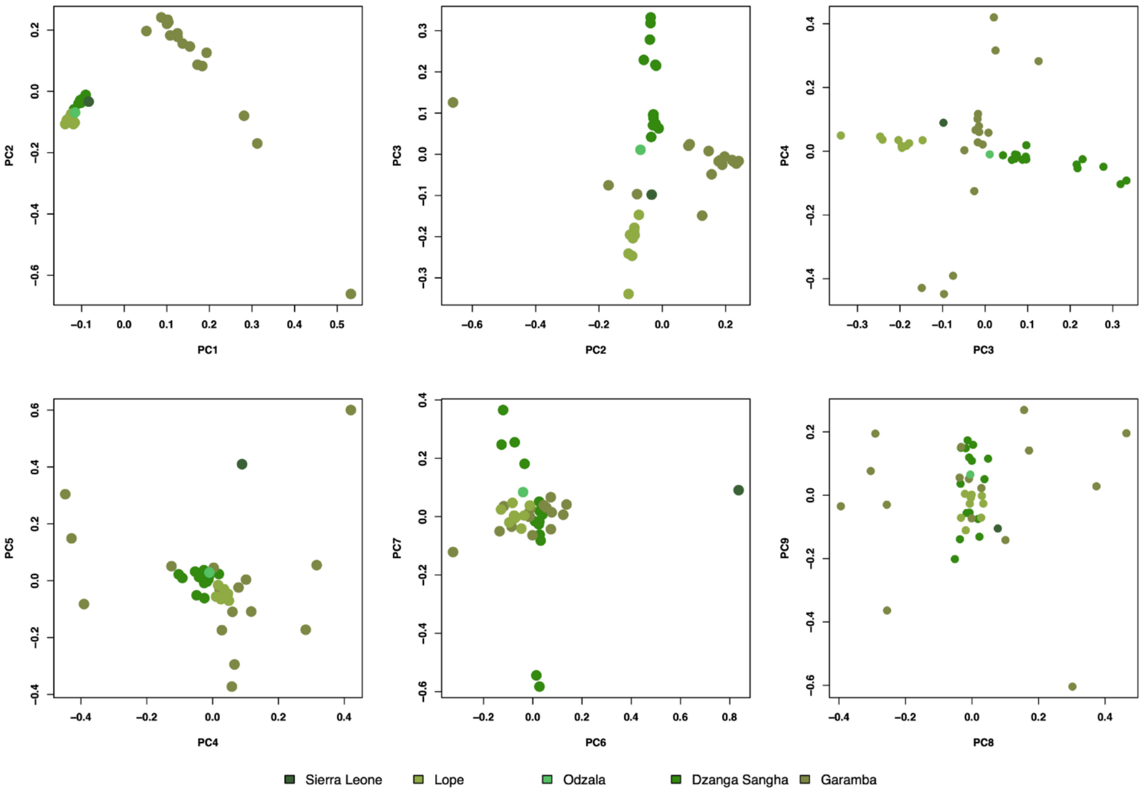

Fig. S23. Population structure of African forest elephants based on the called genotypes. Principal component analysis was performed in PLINK v1.9[8] and shows principal components (PCs) 1 to 9.

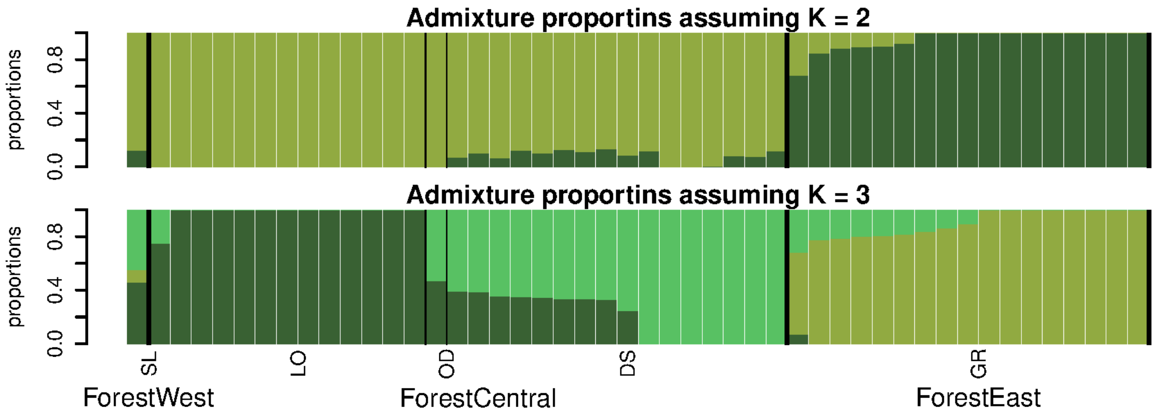

Fig. S24. Admixture analysis of African forest elephants performed in ADMIXTURE v1.3.0[9] on the imputed dataset. Analysis converged up to $K = 3$.

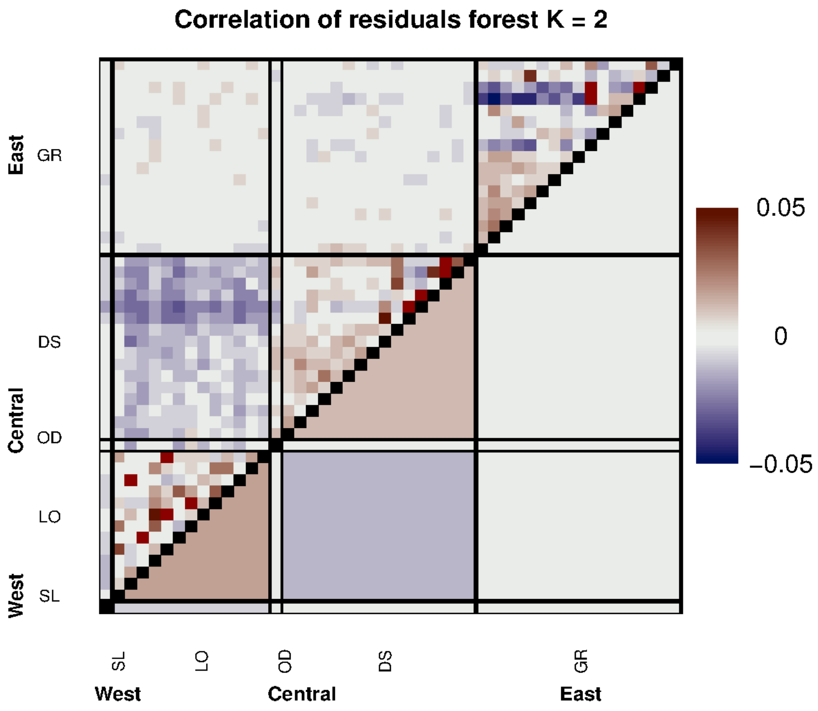

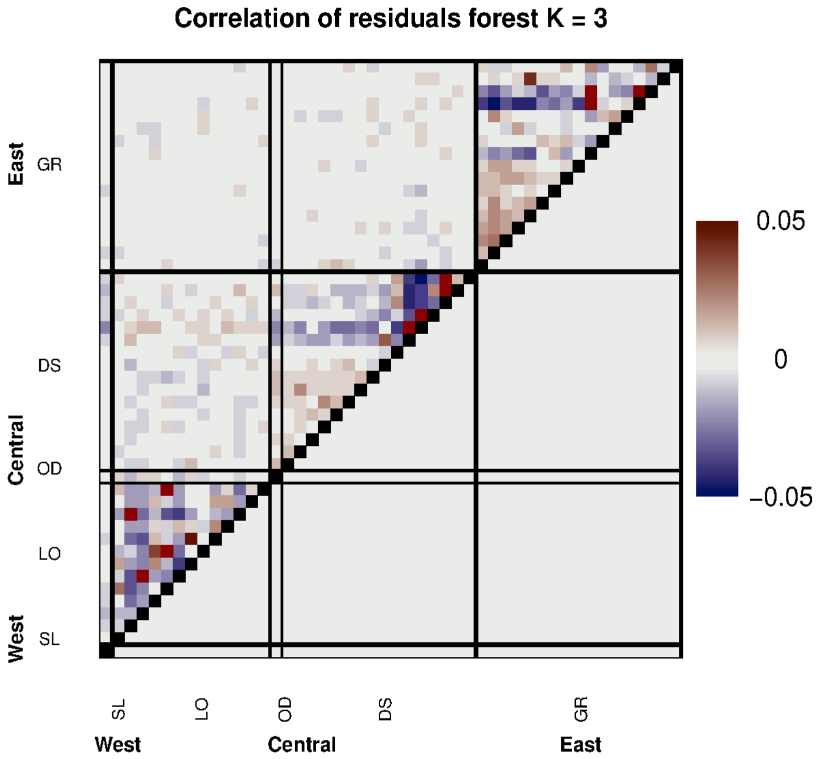

Fig. S25. Evaluation of the model fit of the inferred admixture proportions in forest elephants of the maximum likelihood run for each converged *K* performed in evalAdmix v0.962[10].

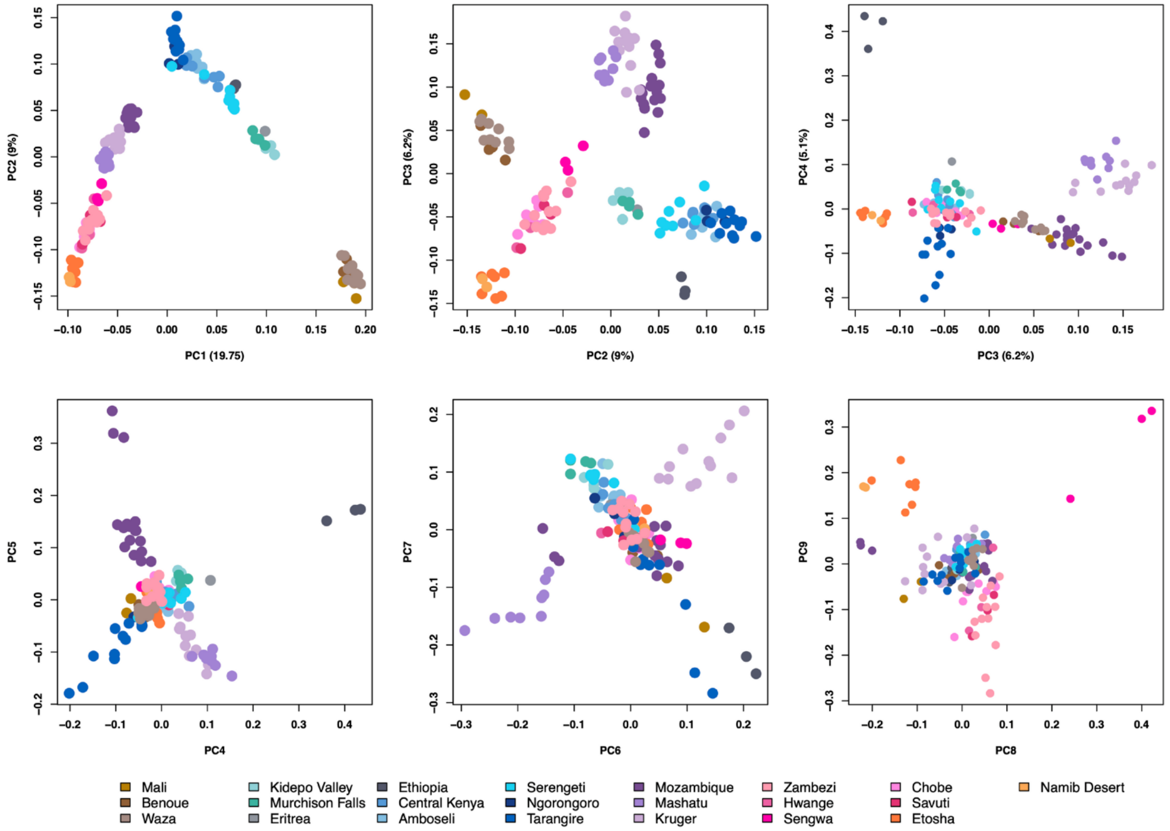

Fig. S26. Population structure of African savanna elephants based on the called genotypes. Principal component analysis was performed in PLINK v1.9[8] and shows principal components (PCs) 1 to 9.

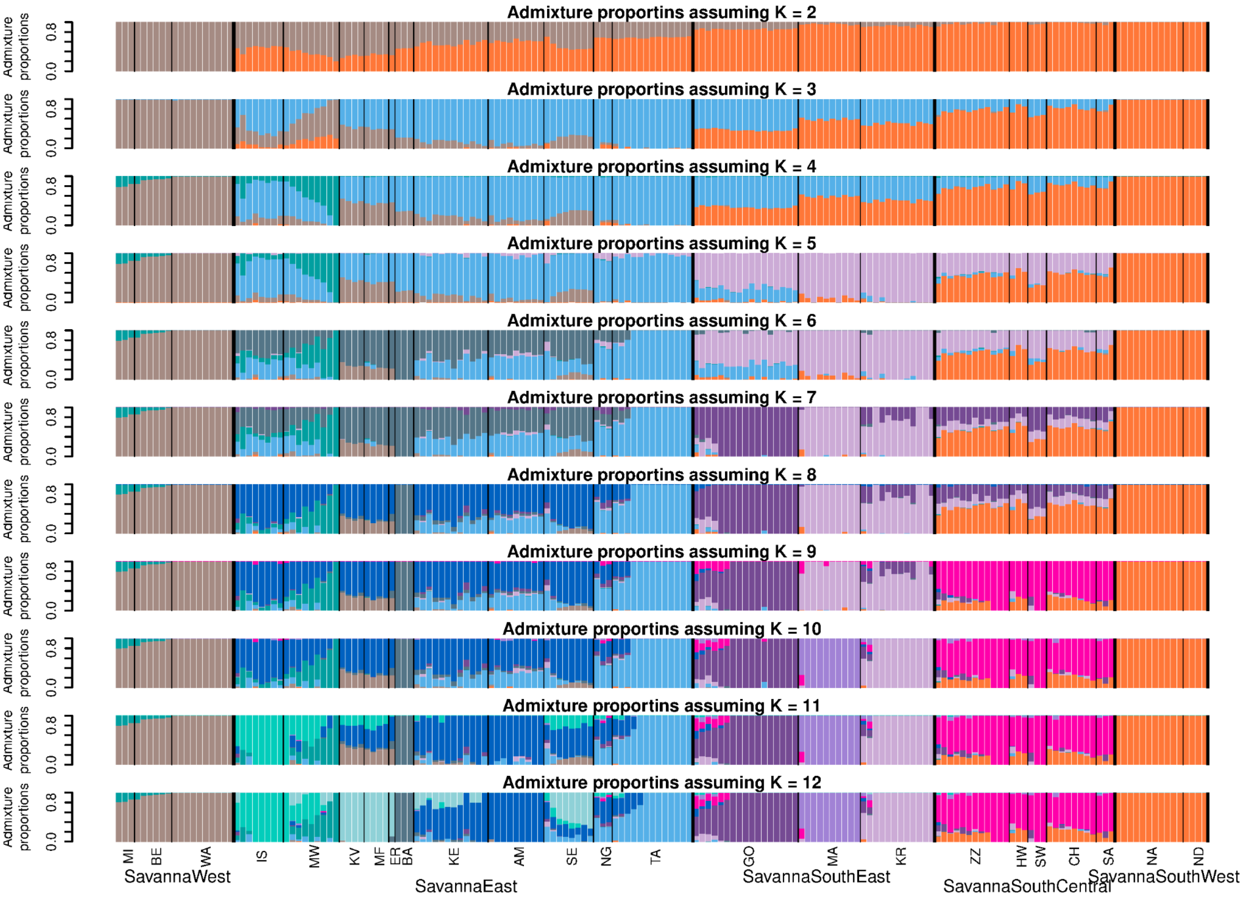

Fig. S27. Admixture analysis of African savanna elephants performed in ADMIXTURE v1.3.0[9] on the imputed dataset. Analysis converged up to *K*=12.

a)

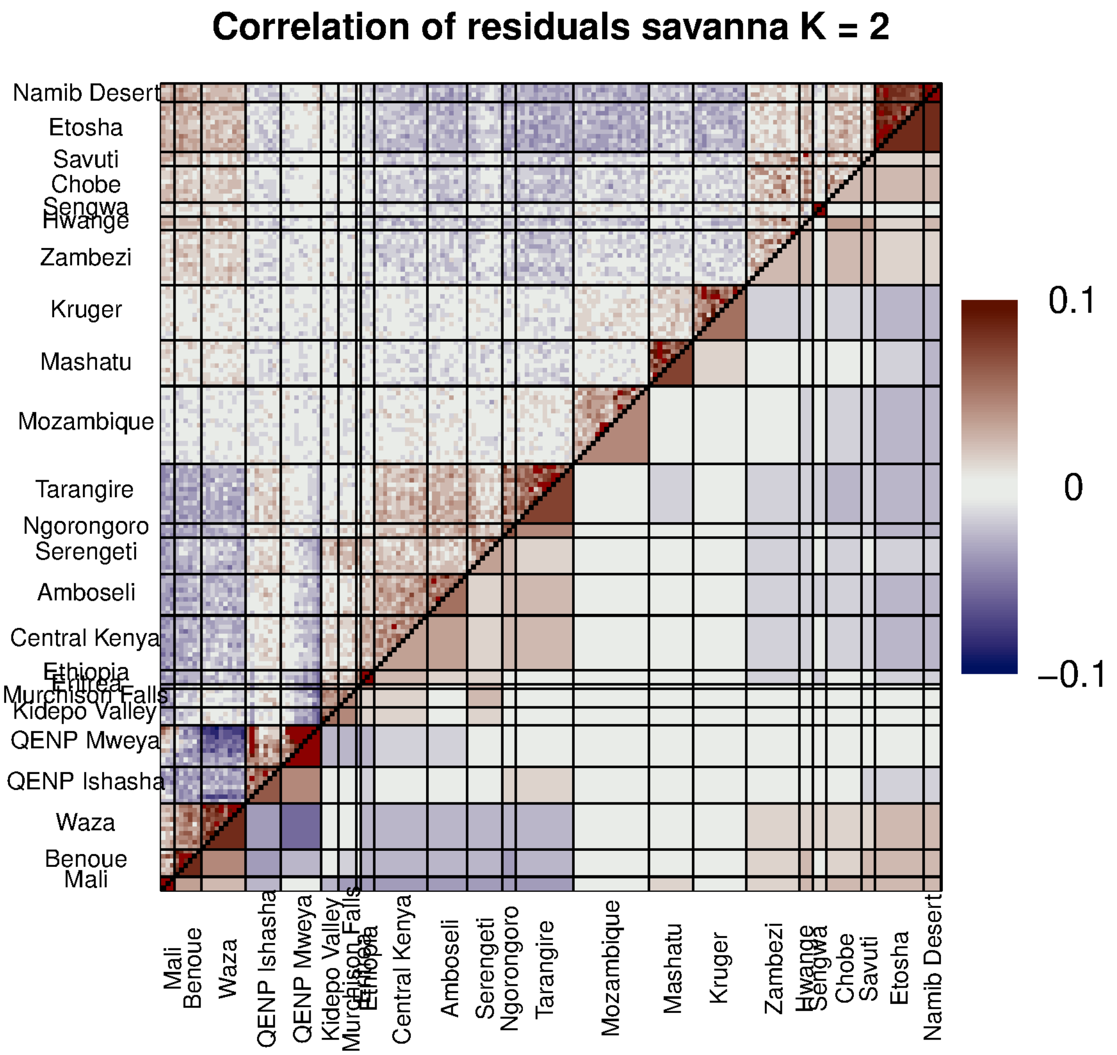

Fig. S28. Evaluation of the model fit of the inferred admixture proportions in savanna elephants of the maximum likelihood run for each converged *K* performed in evalAdmix v0.962[10]. Correlation of residuals for: a) K=2; b) K=3; c) K=4; d) K=5; e) K=6; f) K=7; g) K=8; h) K=9; i) K=10; j) K=11; k) K=12. Due to small sample sizes in some groups, the titles of some rows and columns are overlapping–the populations are ordered horizontally from top to bottom and vertically from right to left in the following order: "Namib Desert, Etosha, Savuti, Chobe, Sengwa, Hwange, Zambezi, Kruger, Mashatu, Mozambique, Tarangire, Ngorongoro, Serengeti, Amboseli, Central Kenya, Ethiopia, Eritrea, Murchison Falls, Kidepo Valley, QENP Mweya, QENP Ishasha, Waza, Benoue, Mali, Garamba, Dzanga Sangha, Odzala, Lope Sierra Leone".

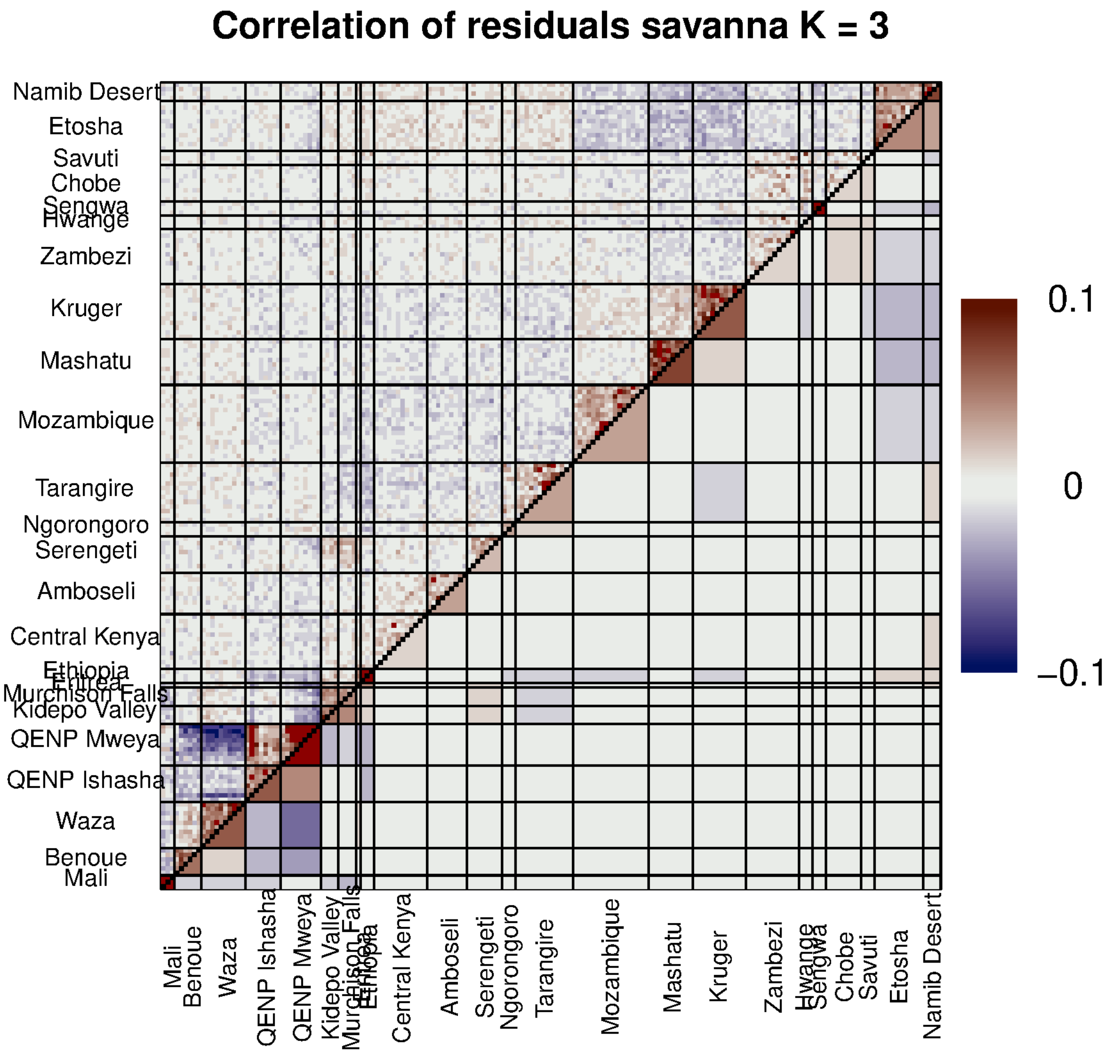
Correlation of residuals savanna K = 3
Namib Desert
Etosha
Savuti
Chobe
Sengwa
Hwange
Zambezi
Kruger
Mashatu
Mozambique
Tarangire
Ngorongoro
Serengeti
Amboseli
Central Kenya
Ethiopia
Eritrea
Murchison Falls
Kidepo Valley
QENP Mweya
QENP Ishasha
Waza
Benoue
Mali
0.1
0
−0.1

Fig. S28. b)

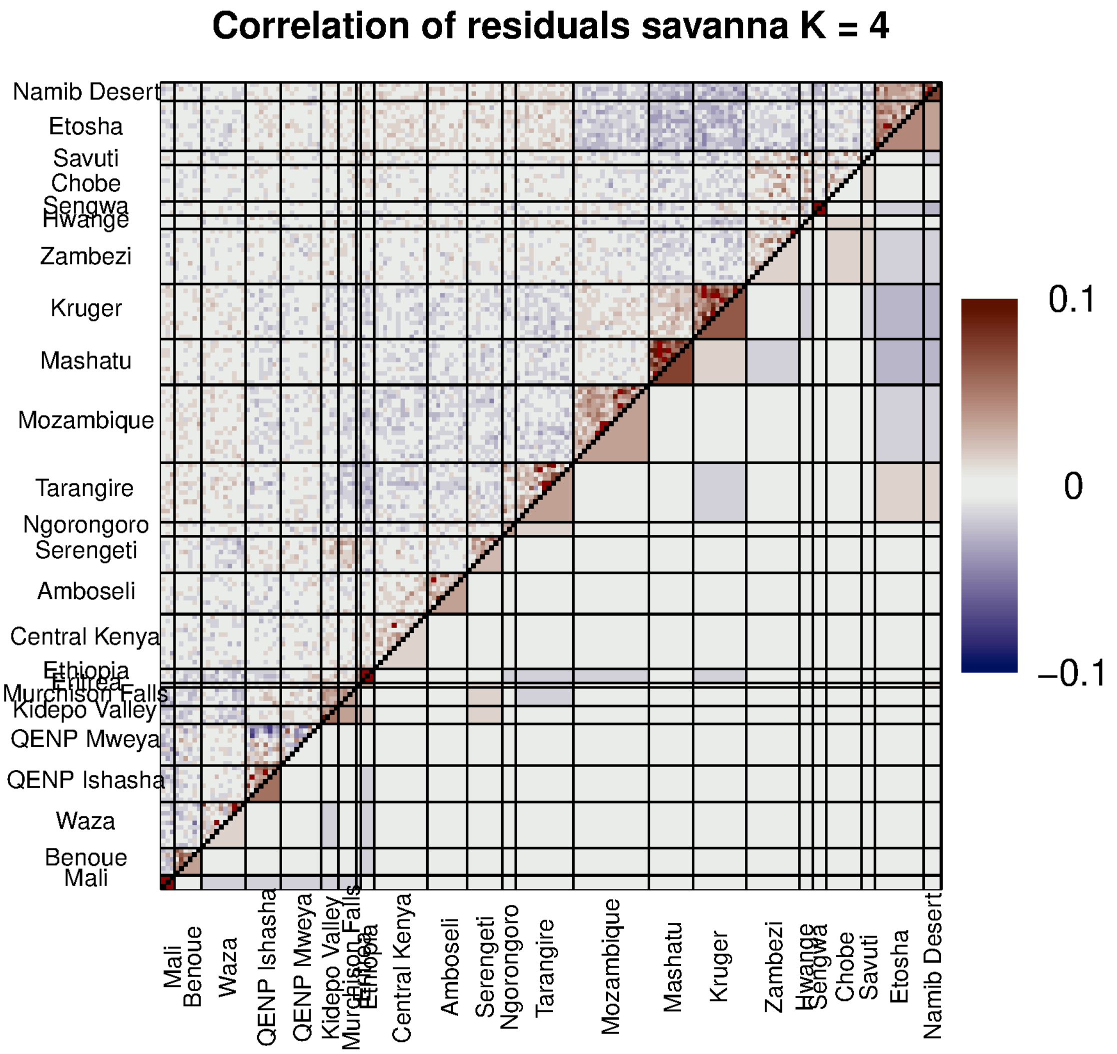

Correlation of residuals savanna K = 4
Namib Desert
Etosha
Savuti
Chobe
Sengwa
Hwange
Zambezi
Kruger
Mashatu
Mozambique
Tarangire
Ngorongoro
Serengeti
Amboseli
Central Kenya
Ethiopia
Eritrea
Murchison Falls
Kidepo Valley
QENP Mweya
QENP Ishasha
Waza
Benoue
Mali
0.1
0
−0.1

Fig. S28. c)

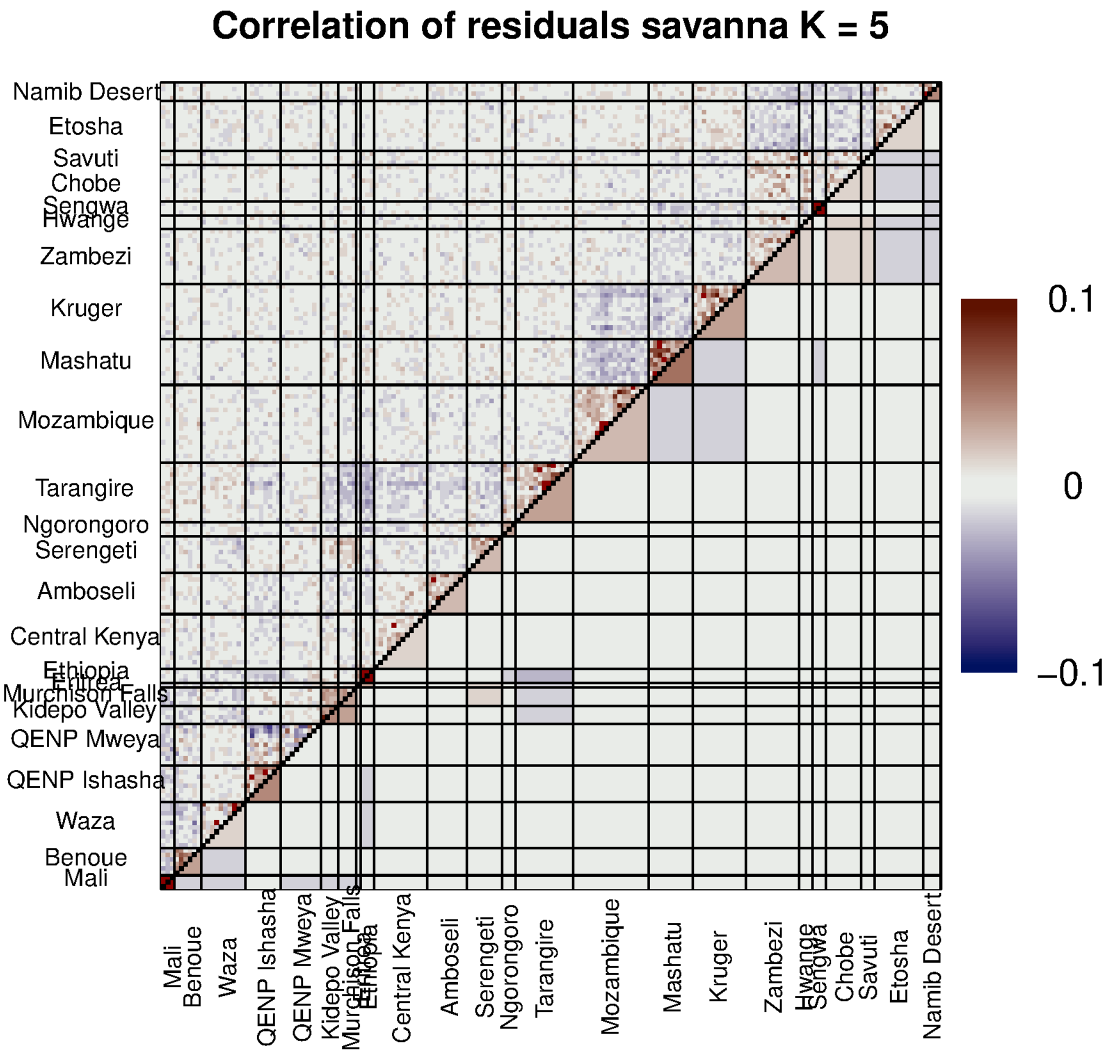

Correlation of residuals savanna K = 5
Namib Desert
Etosha
Savuti
Chobe
Sengwa
Hwange
Zambezi
Kruger
Mashatu
Mozambique
Tarangire
Ngorongoro
Serengeti
Amboseli
Central Kenya
Ethiopia
Eritrea
Murchison Falls
Kidepo Valley
QENP Mweya
QENP Ishasha
Waza
Benoue
Mali
0.1
0
−0.1

Fig. S28. d)

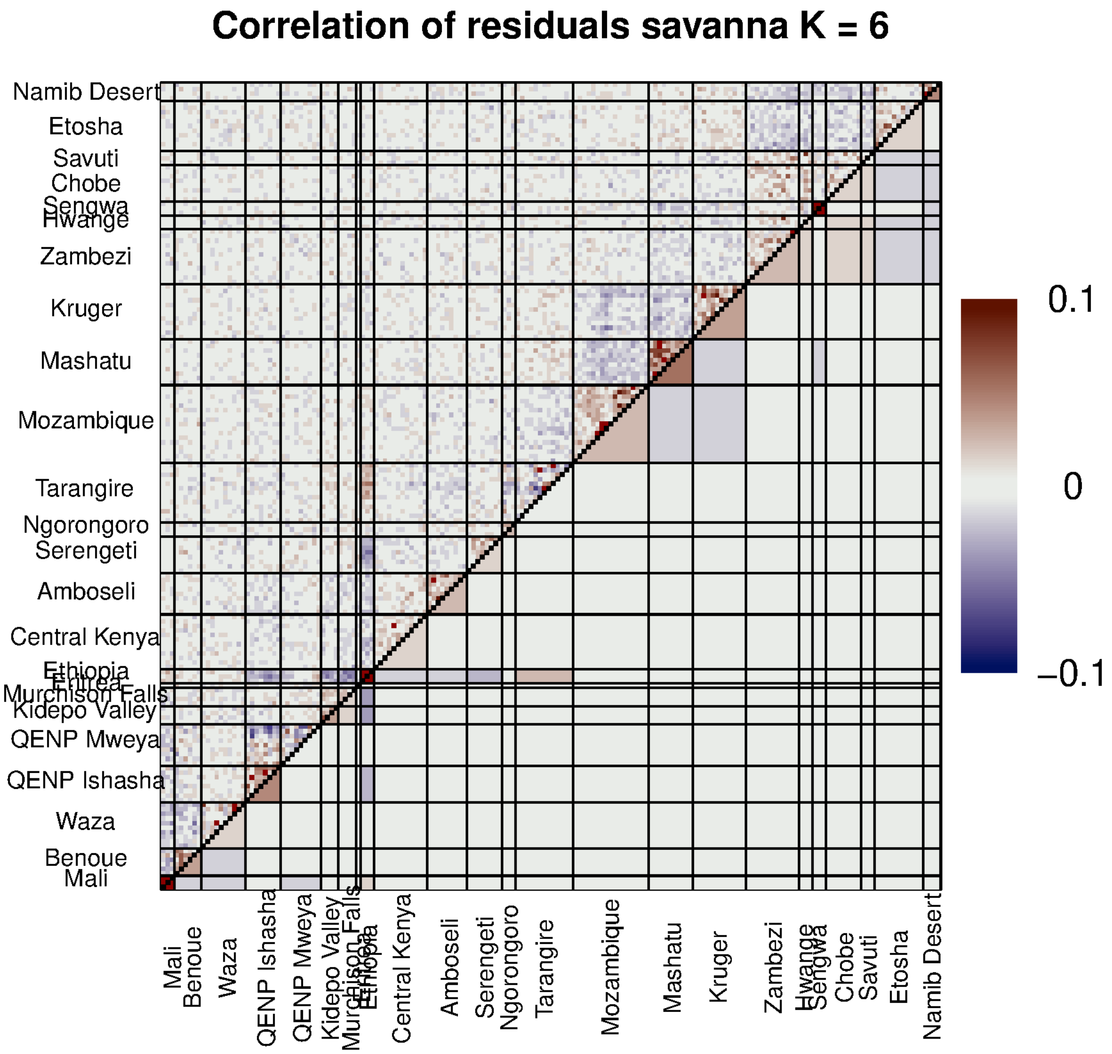
Correlation of residuals savanna K = 6
Namib Desert
Etosha
Savuti
Chobe
Sengwa
Hwange
Zambezi
Kruger
Mashatu
Mozambique
Tarangire
Ngorongoro
Serengeti
Amboseli
Central Kenya
Ethiopia
Eritrea
Murchison Falls
Kidepo Valley
QENP Mweya
QENP Ishasha
Waza
Benoue
Mali
0.1
0
−0.1

Fig. S28. e)

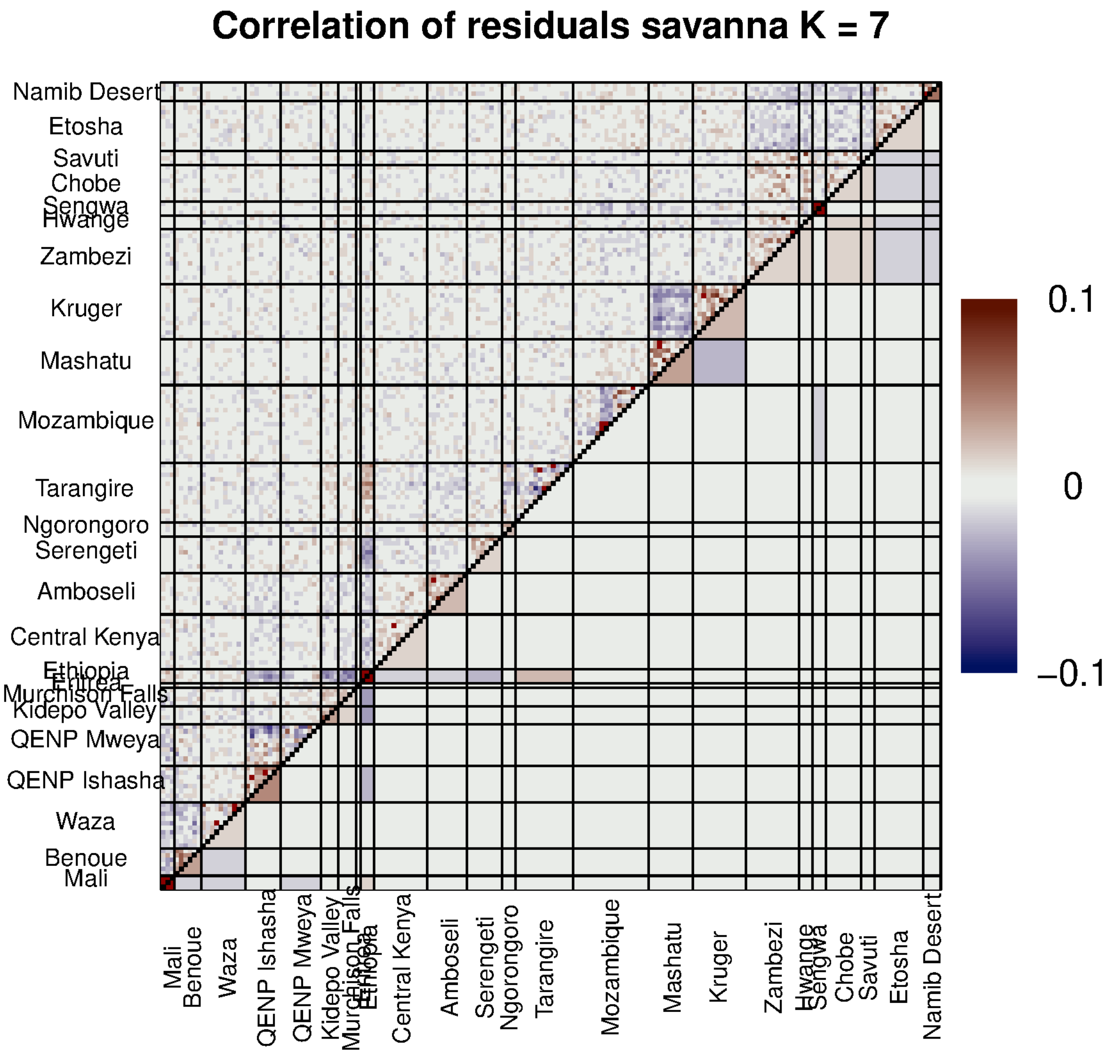

Correlation of residuals savanna K = 7
Namib Desert
Etosha
Savuti
Chobe
Sengwa
Hwange
Zambezi
Kruger
Mashatu
Mozambique
Tarangire
Ngorongoro
Serengeti
Amboseli
Central Kenya
Ethiopia
Eritrea
Murchison Falls
Kidepo Valley
QENP Mweya
QENP Ishasha
Waza
Benoue
Mali
0.1
0
−0.1

Fig. S28. f)

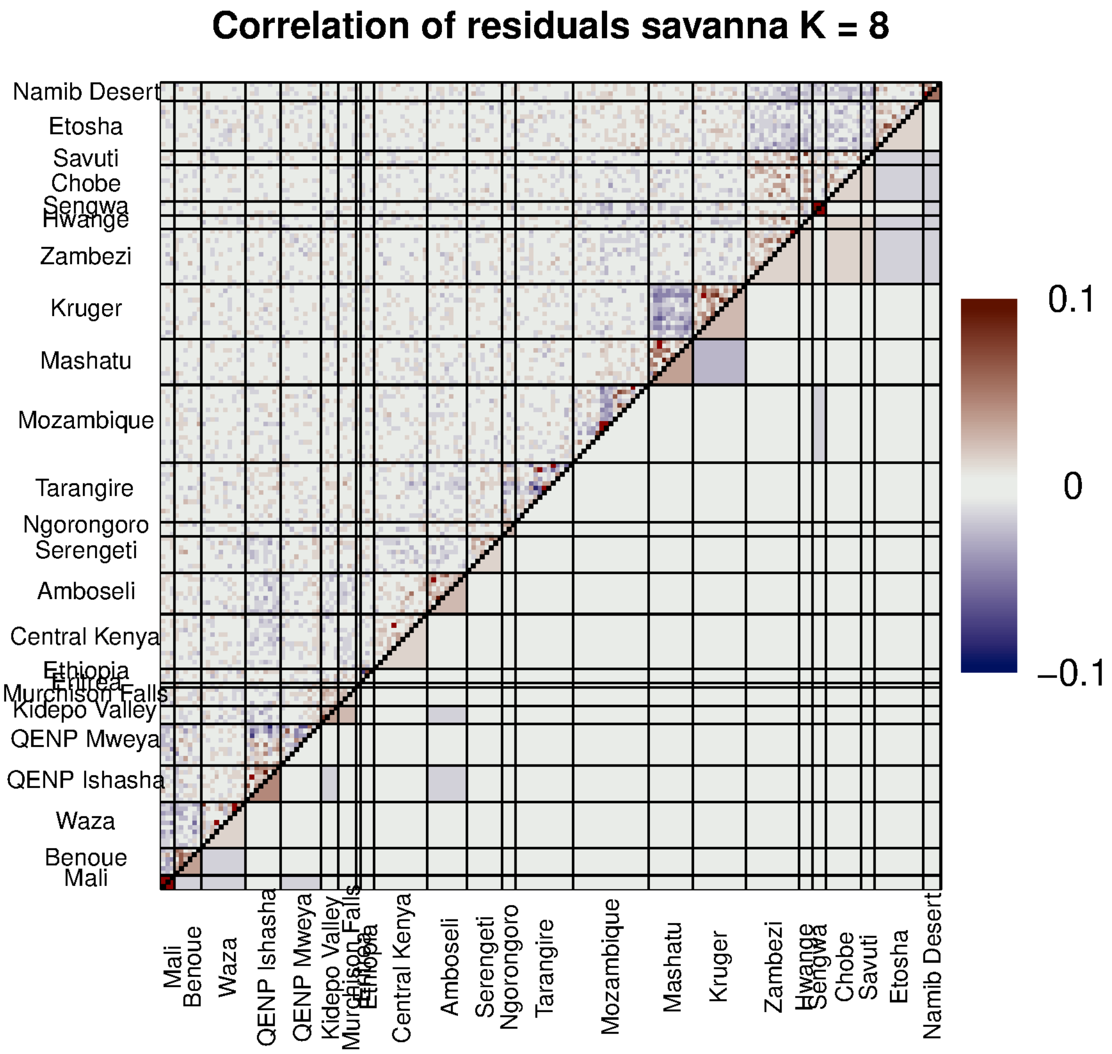

Correlation of residuals savanna K = 8
Namib Desert
Etosha
Savuti
Chobe
Sengwa
Hwange
Zambezi
Kruger
Mashatu
Mozambique
Tarangire
Ngorongoro
Serengeti
Amboseli
Central Kenya
Ethiopia
Eritrea
Murchison Falls
Kidepo Valley
QENP Mweya
QENP Ishasha
Waza
Benoue
Mali
0.1
0
−0.1

Fig. S28. g)

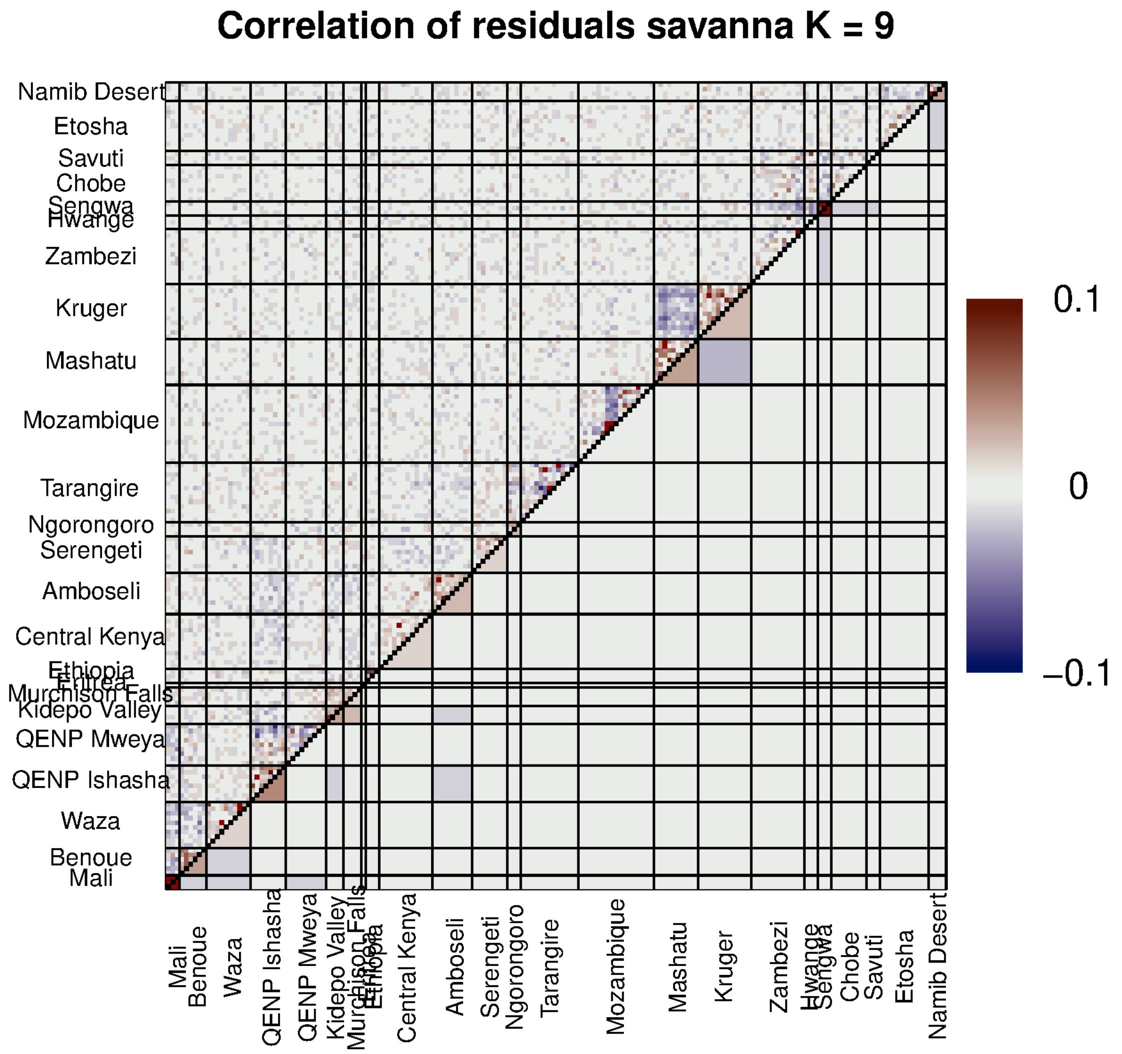

Correlation of residuals savanna K = 9
Namib Desert
Etosha
Savuti
Chobe
Sengwa
Hwange
Zambezi
Kruger
Mashatu
Mozambique
Tarangire
Ngorongoro
Serengeti
Amboseli
Central Kenya
Ethiopia
Eritrea
Murchison Falls
Kidepo Valley
QENP Mweya
QENP Ishasha
Waza
Benoue
Mali
0.1
0
−0.1

Fig. S28. h)

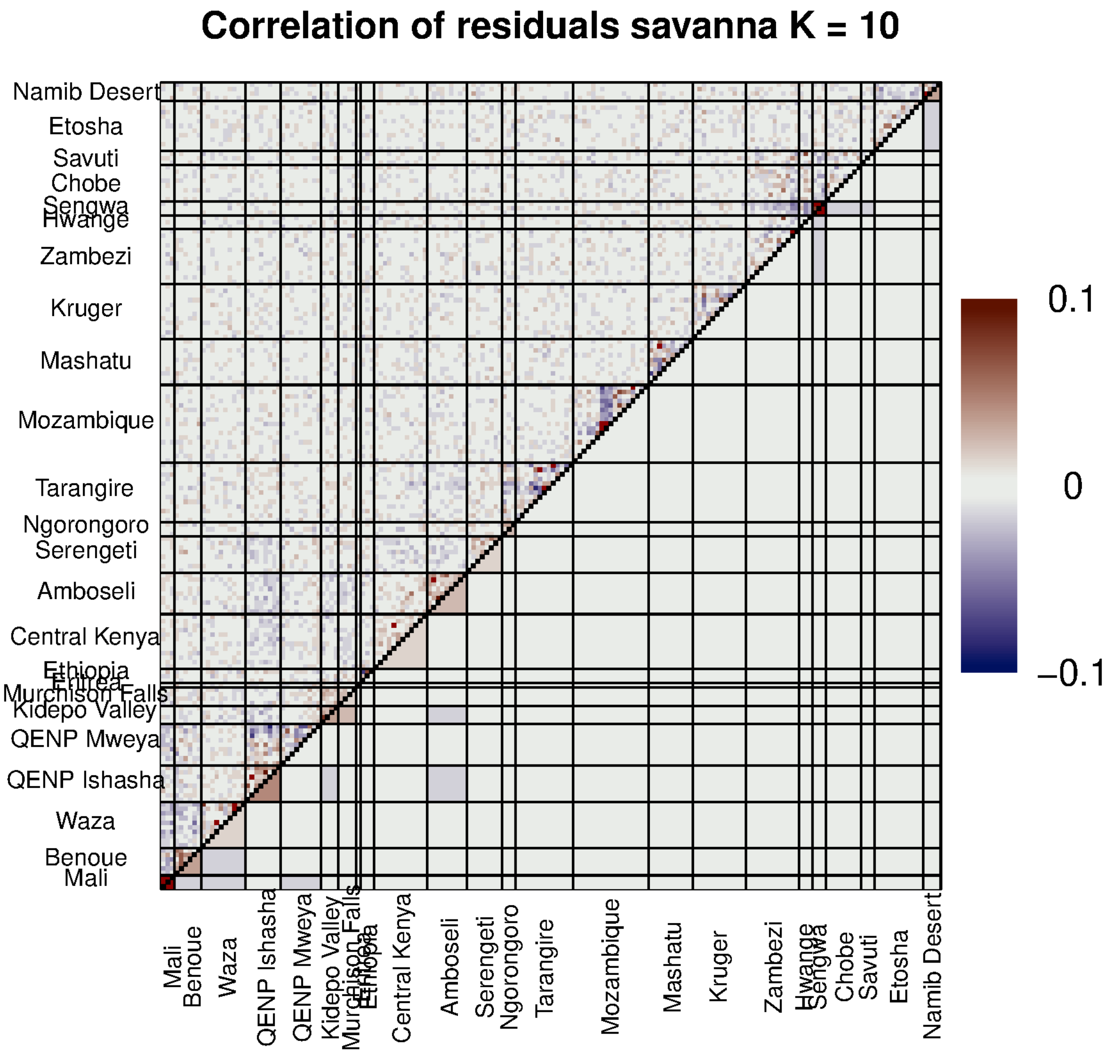
Correlation of residuals savanna K = 10
Namib Desert
Etosha
Savuti
Chobe
Sengwa
Hwange
Zambezi
Kruger
Mashatu
Mozambique
Tarangire
Ngorongoro
Serengeti
Amboseli
Central Kenya
Ethiopia
Eritrea
Murchison Falls
Kidepo Valley
QENP Mweya
QENP Ishasha
Waza
Benoue
Mali
0.1
0
−0.1

Fig. S28. i)

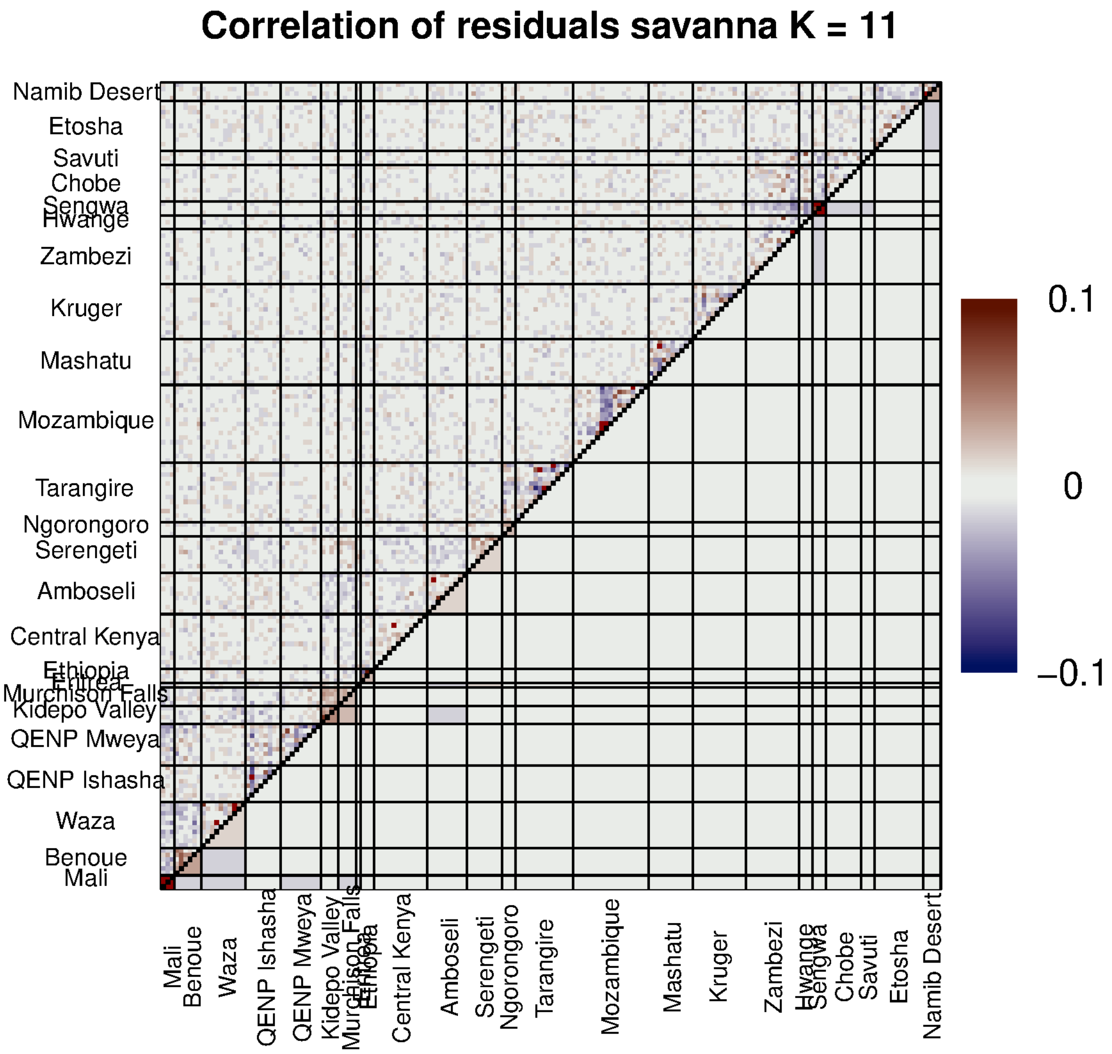
Correlation of residuals savanna K = 11
Namib Desert
Etosha
Savuti
Chobe
Sengwa
Hwange
Zambezi
Kruger
Mashatu
Mozambique
Tarangire
Ngorongoro
Serengeti
Amboseli
Central Kenya
Ethiopia
Eritrea
Murchison Falls
Kidepo Valley
QENP Mweya
QENP Ishasha
Waza
Benoue
Mali
0.1
0
−0.1

Fig. S28. j)

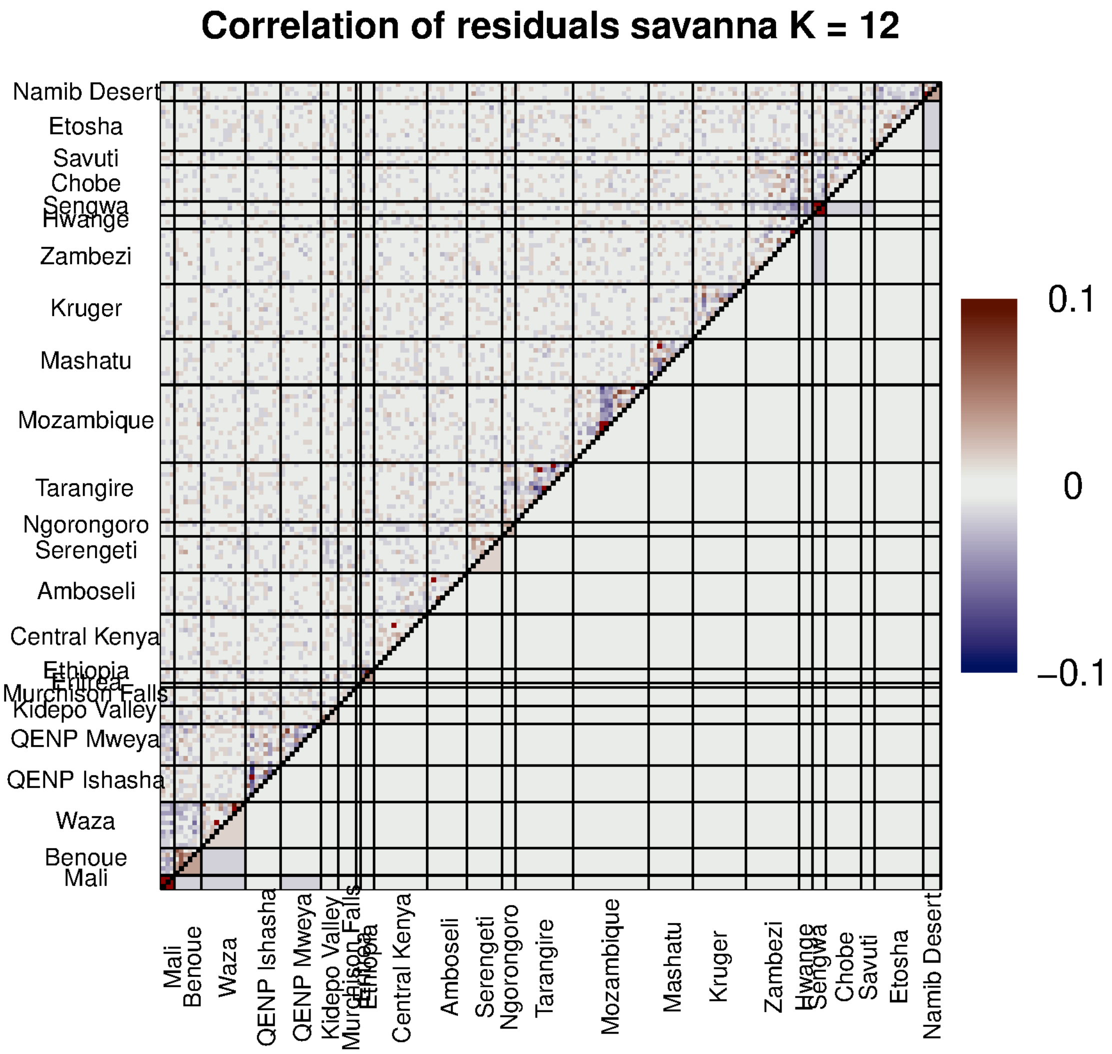
Correlation of residuals savanna K = 12
Namib Desert
Etosha
Savuti
Chobe
Sengwa
Hwange
Zambezi
Kruger
Mashatu
Mozambique
Tarangire
Ngorongoro
Serengeti
Amboseli
Central Kenya
Ethiopia
Eritrea
Murchison Falls
Kidepo Valley
QENP Mweya
QENP Ishasha
Waza
Benoue
Mali
0.1
0
−0.1

Fig. S28. k)

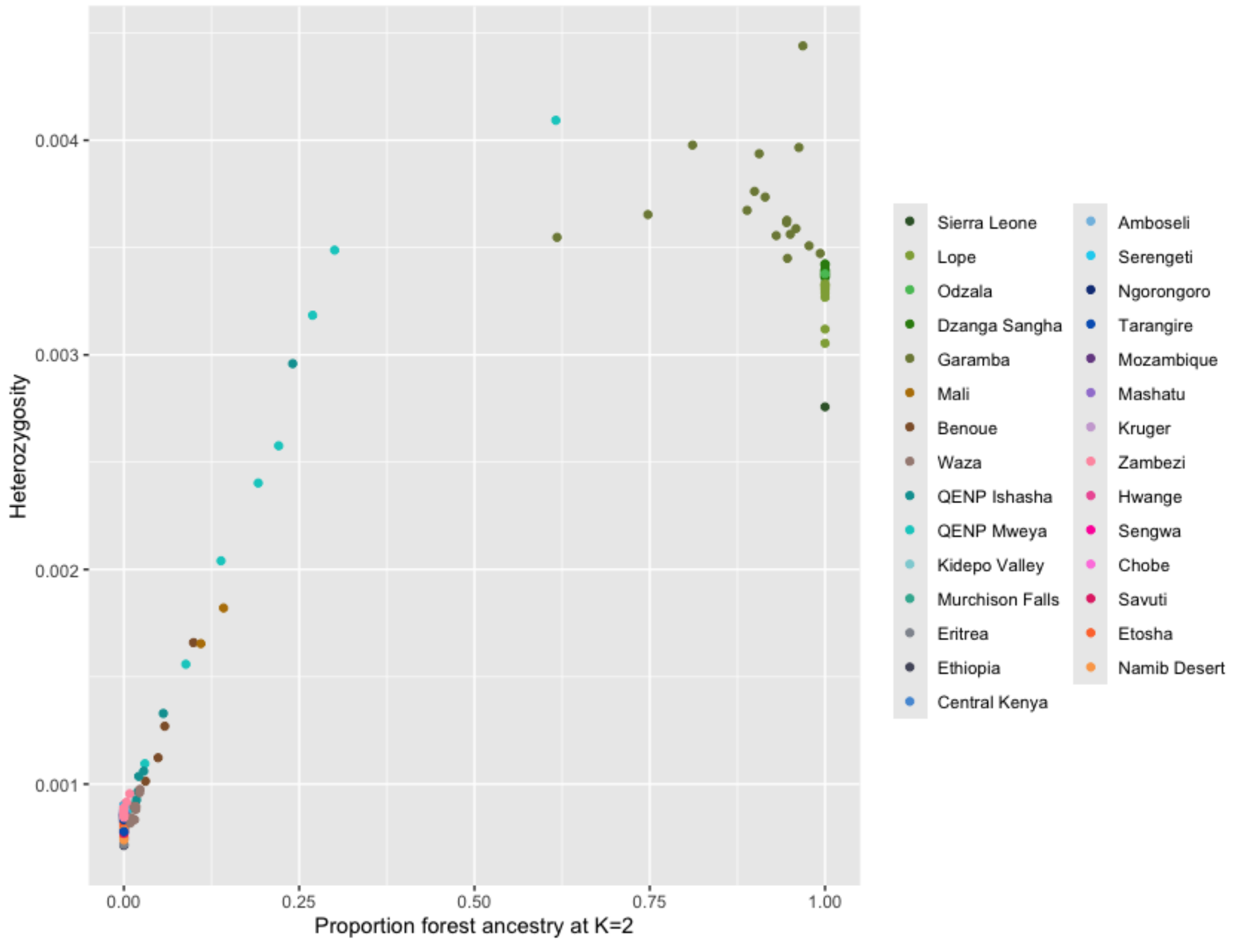

Fig. S29. Genome-wide heterozygosity plotted against the proportion of forest ancestry as estimated at K = 2 in the Admixture analyses of all African elephants, highlighting that the elevated levels of heterozygosity of certain savanna elephant individuals are proportional to their forest ancestry.

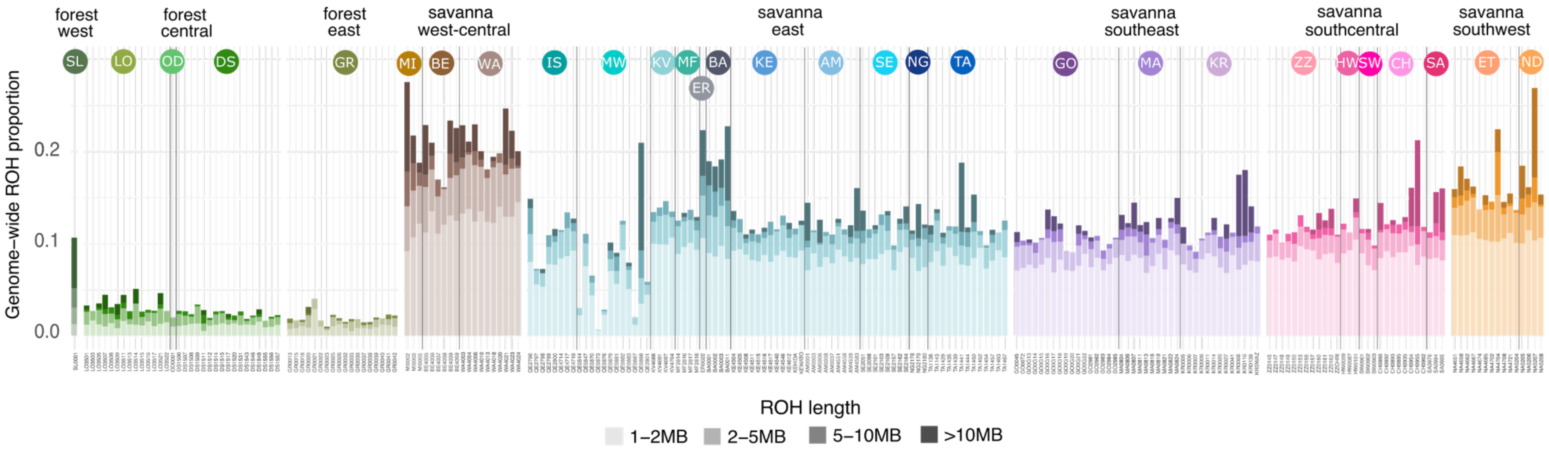

Fig. S30. Individually-labeled runs of homozygosity (ROH) estimated in PLINK v1.9[8]. Savanna elephants have overall higher levels of inbreeding than forest elephants. Populations known to be more isolated (Sierra Leone: SL, Ethiopia: BA, Eritrea: ER, Namibia: ET, ND) have a higher proportion of ROH. Population codes correspond to Fig. S1.

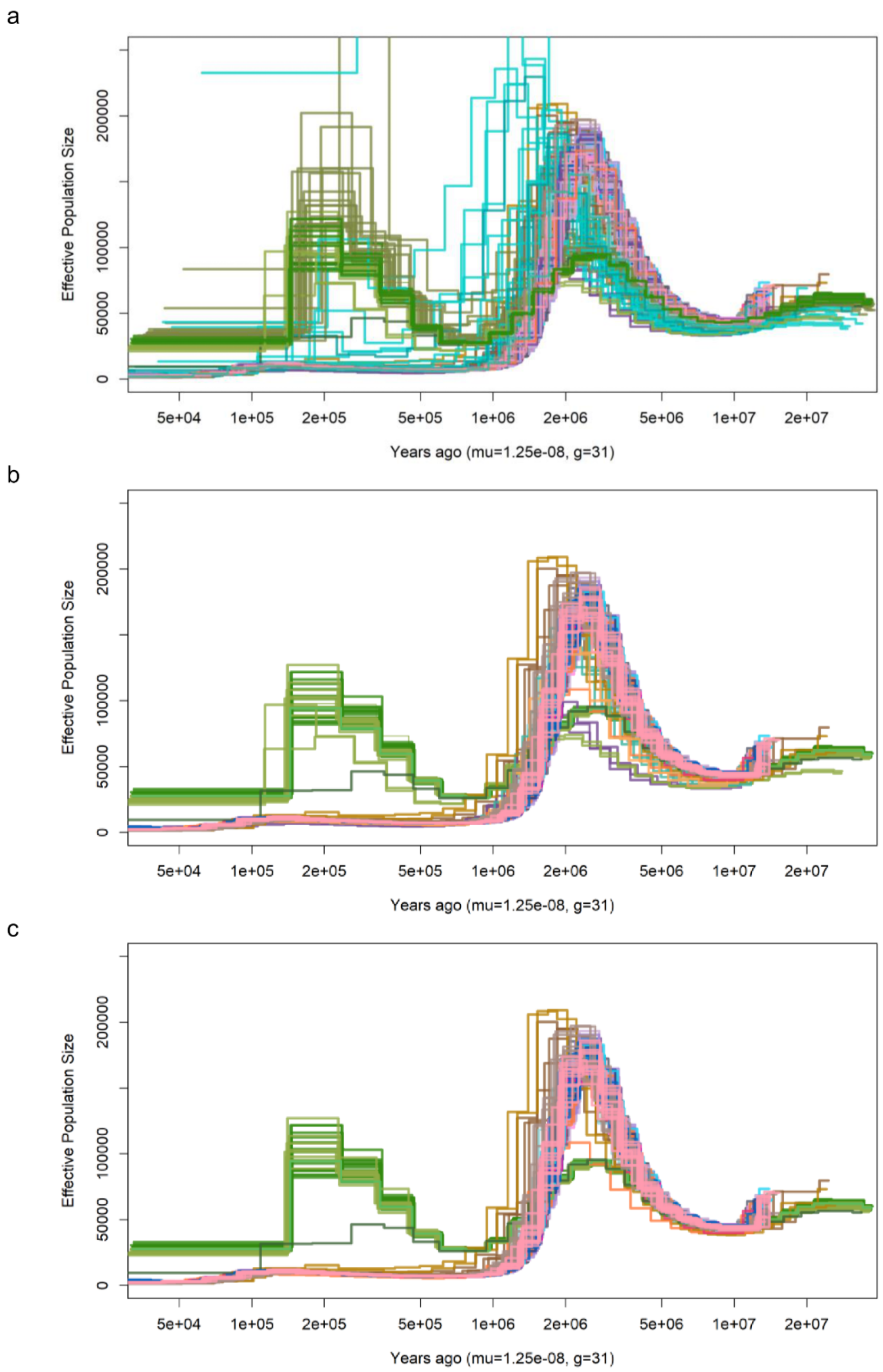

Fig. S31. Changes in effective population size over time estimated using the pairwise sequentially Markovian coalescent (PSMC)[31] method. Colors correspond to population locations as shown in Fig. 1 and Fig. S1. a) All high coverage samples. b) Excluding hybrid populations (Garamba and QENP). c) Excluding samples below 15X and hybrid populations.

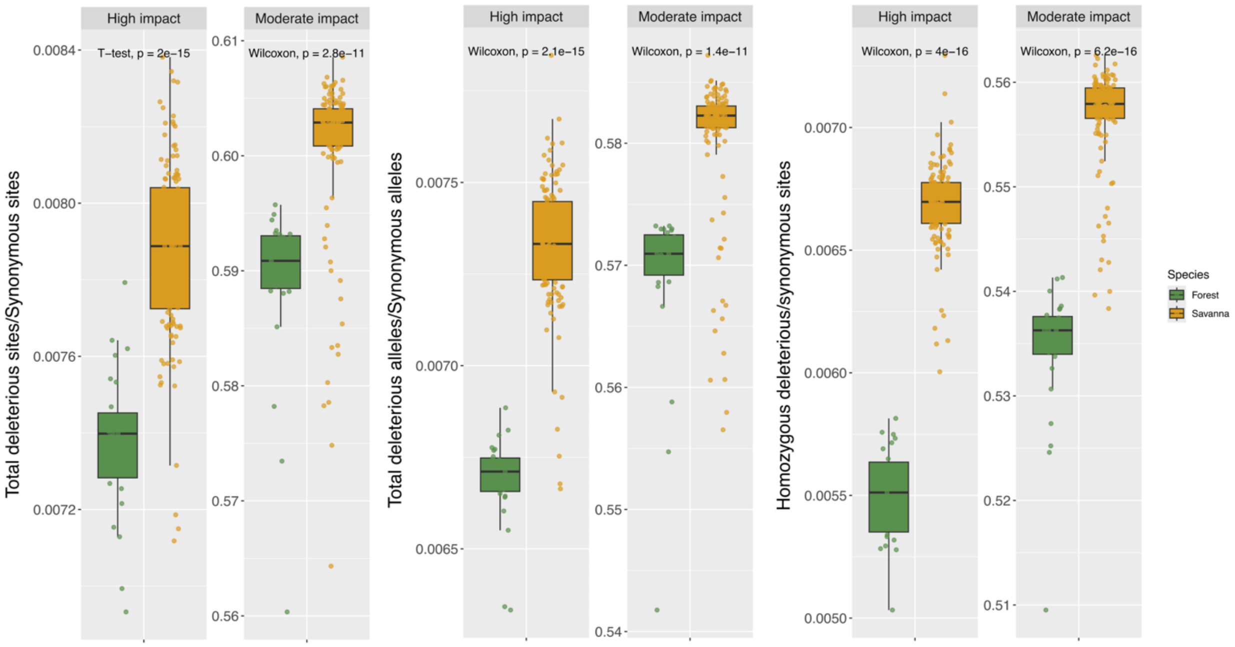

Fig. S32. Genetic load measured as the number of high impact (loss-of-function) and moderate impact (missense) heterozygous derived sites normalized by the heterozygous synonymous derived alleles as inferred in SnpEff [32] compared between species (forest elephant in green and savanna elephant in orange). Populations with larger proportions of mixed ancestry (Garamba, QENP, Benoue, Waza, and Mali) were removed from the comparison. Boxplot shows the median (central line) and 25th and 75th percentiles (box), whiskers extend to the minimum and maximum values within 1.5-times the interquartile range. The two species significantly differ in both the high and moderate impact load. Normality was assessed using Shapiro–Wilk tests; two-sided Welch's t-tests were used for normally distributed data, and Wilcoxon rank-sum tests were applied when normality assumptions were violated.

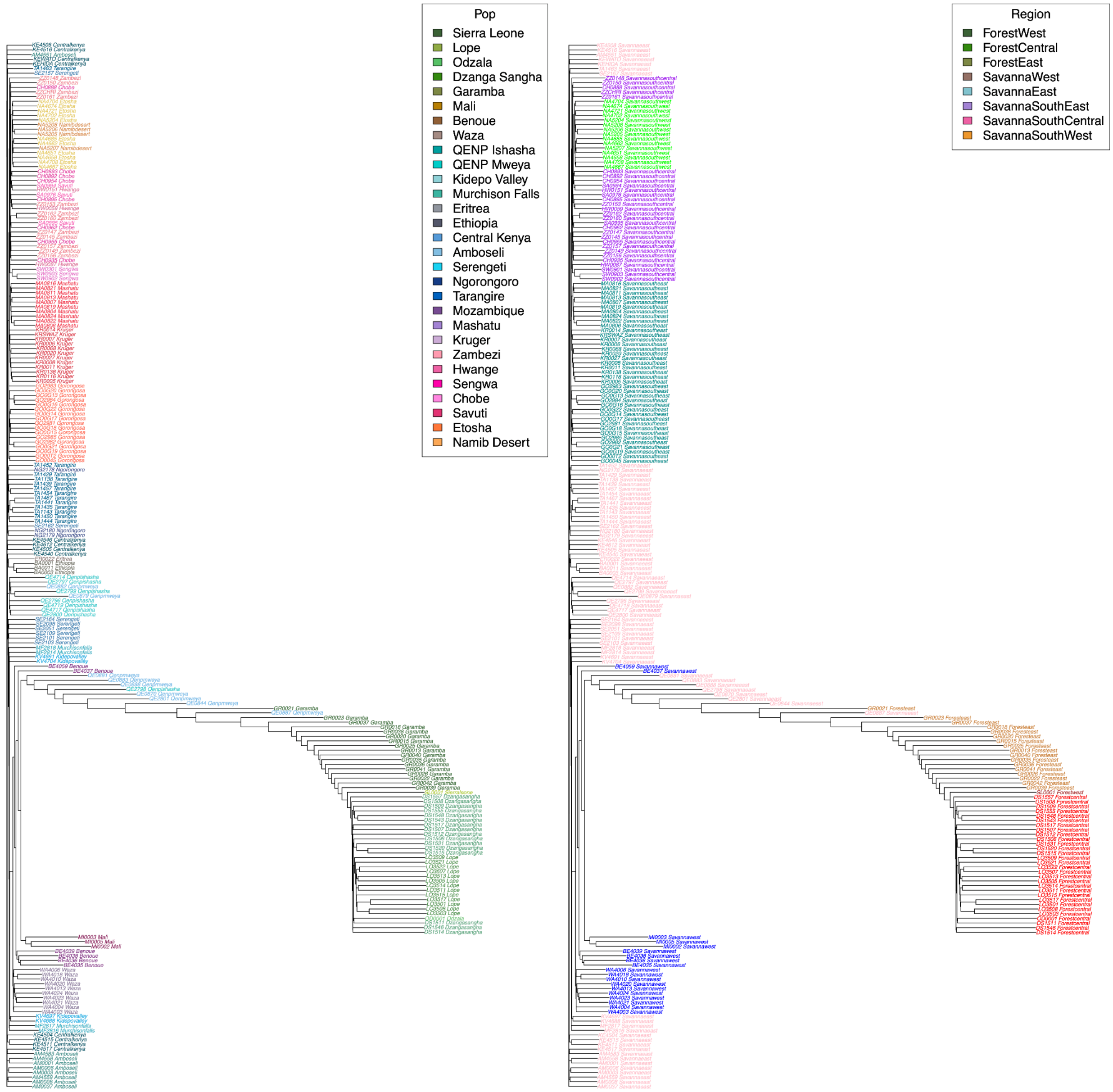

Fig. S33. A neighbor joining (NJ) tree was constructed from an identity-by-state (IBS) matrix as a measure of the pairwise genetic distance between individuals in PLINK v1.9.0[8].

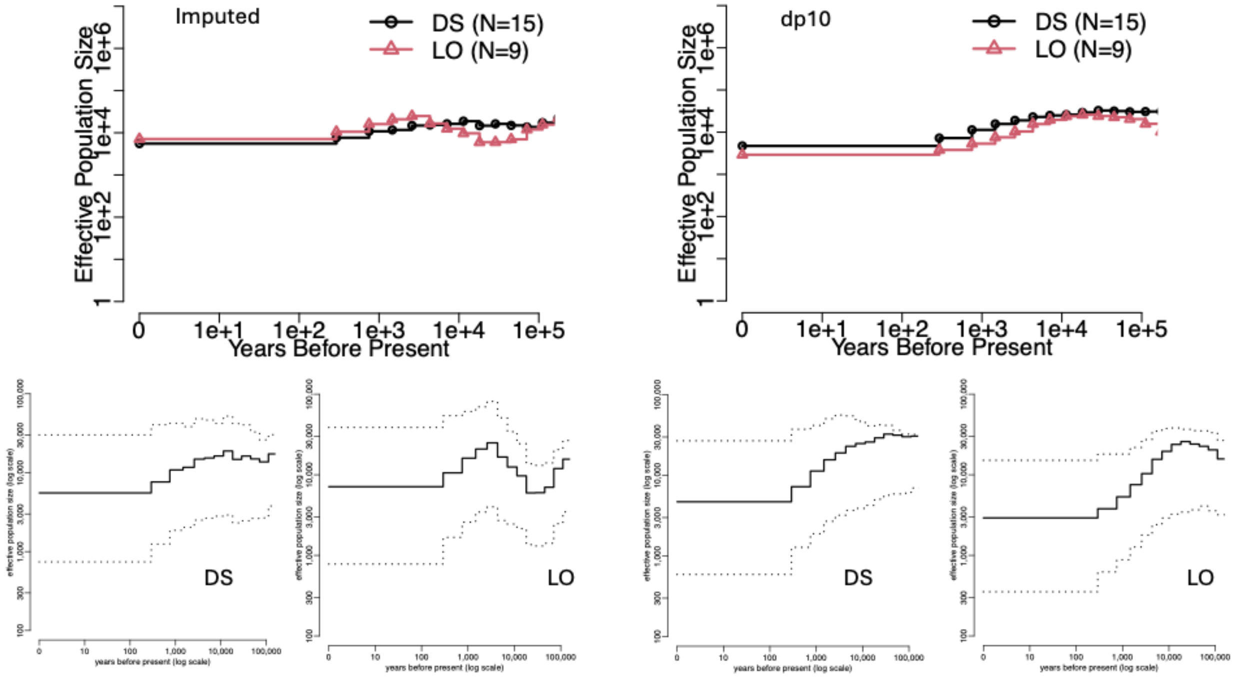

Fig. S34. Recent demographic history inferred in popSizeABC[33] for two populations of forest elephants that had genomic data for a sufficient number of individuals (Lope and Dzanga Sangha). Each panel shows the population trajectory through time. The top row includes both populations (LO and DS), while the bottom panel shows them separately. The left side shows the analysis based on the imputed dataset, while the right side shows results based on called genotypes.

Supplementary References

1. Sayre, R. G. *et al.* A new map of standardized terrestrial ecosystems of Africa. *Afr. Geogr. Rev.* (2013).
2. Gobush, K. S. *et al. Loxodonta africana* (amended version of 2021 assessment). *The IUCN Red List of Threatened Species 2022* (2022) doi:10.2305/iucn.uk.2021-1.rlts.t181008073a181022663.en.
3. Gobush, K. S. *et al. Loxodonta cyclotis* (errata version published in 2021). *The IUCN Red List of Threatened Species 2021* (2021) doi:10.2305/iucn.uk.2021-1.rlts.t181007989a181019888.en.
4. Esri. Terrain with labels. (2024).
5. Esri. Terrain. (2024).
6. Esri. Africa Terrestrial Ecosystems. (2019).
7. Esri Africa. Africa countries. (2018).
8. Purcell, S. *et al.* PLINK: a tool set for whole-genome association and population-based linkage analyses. *Am. J. Hum. Genet.* **81**, 559–575 (2007).
9. Alexander, D. H., Novembre, J. & Lange, K. Fast model-based estimation of ancestry in unrelated individuals. *Genome Res* **19**, 1655–1664 (2009).
10. Garcia-Erill, G. & Albrechtsen, A. Evaluation of model fit of inferred admixture proportions. *Mol. Ecol. Resour.* **20**, 936–949 (2020).
11. Bhatia, G., Patterson, N., Sankararaman, S. & Price, A. L. Estimating and interpreting FST: the impact of rare variants. *Genome Res.* **23**, 1514–1521 (2013).
12. Liu, X. *et al.* Introgression and disruption of migration routes have shaped the genetic integrity of wildebeest populations. *Nat. Commun.* **15**, 2921 (2024).
13. Durand, E. Y., Patterson, N., Reich, D. & Slatkin, M. Testing for ancient admixture between closely related populations. *Mol. Biol. Evol.* **28**, 2239–2252 (2011).
14. Green, R. E. *et al.* A draft sequence of the Neandertal genome. *Science* **328**, 710–722 (2010).
15. R Core Team. R: A Language and Environment for Statistical Computing (2025).
16. Wickham, H. ggplot2: Elegant Graphics for Data Analysis (2016).

17. Garcia-Erill, G., Hanghøj, K., Heller, R., Wiuf, C. & Albrechtsen, A. Estimating admixture pedigrees of recent hybrids without a contiguous reference genome. *Mol. Ecol. Resour.* **23**, 1604–1619 (2023).

18. Posit team. RStudio: Integrated Development Environment for R. Preprint at http://www.posit.co/ (2025).

19. Oksanen, J. *et al.* The Vegan Package: Community Ecology Package (2008).

20. Olson, D. M. *et al.* Terrestrial ecoregions of the world: A new map of life on earth. Bioscience **51**, 933 (2001).

21. Wickham, H., François, R., Henry, L., Müller, K. & Vaughan, D. dplyr: A Grammar of Data Manipulation (2025).

22. Pebesma, E. Simple Features for R: Standardized Support for Spatial Vector Data. *The R Journal* vol. 10 439–446 (2018).

23. Pebesma, E. & Bivand, R. *Spatial Data Science*. (Chapman and Hall/CRC, New York, 2023).

24. Hijmans, R. geosphere: Spherical Trigonometry (2024).

25. Patterson, N. *et al.* Ancient Admixture in Human History. *Genetics* **192**, 1065–1093 (2012).

26. Pickrell, J. K. & Pritchard, J. K. Inference of population splits and mixtures from genome-wide allele frequency data. *PLoS Genet.* **8**, e1002967 (2012).

27. Nguyen, L.-T., Schmidt, H. A., von Haeseler, A. & Minh, B. Q. IQ-TREE: a fast and effective stochastic algorithm for estimating maximum-likelihood phylogenies. *Mol. Biol. Evol.* **32**, 268–274 (2015).

28. Kalyaanamoorthy, S., Minh, B. Q., Wong, T. K. F., von Haeseler, A. & Jermiin, L. S. ModelFinder: fast model selection for accurate phylogenetic estimates. *Nat. Methods* **14**, 587–589 (2017).

29. Petkova, D., Novembre, J. & Stephens, M. Visualizing spatial population structure with estimated effective migration surfaces. *Nat. Genet.* **48**, 94–100 (2016).

30. South, A. rworldmap: A New R package for Mapping Global Data. The R Journal, 3(1), 35-43 (2011).

31. Li, H. & Durbin, R. Inference of human population history from individual whole-genome sequences. *Nature* **475**, 493–496 (2011).

32. Cingolani, P. *et al.* A program for annotating and predicting the effects of single nucleotide polymorphisms, SnpEff: SNPs in the genome of *Drosophila melanogaster* strain w1118; iso-2; iso-3. *Fly* **6**, 80–92 (2012).

33. Boitard, S., Rodríguez, W., Jay, F., Mona, S. & Austerlitz, F. Inferring Population Size History from Large Samples of Genome-Wide Molecular Data - An Approximate Bayesian Computation Approach. *PLoS Genet* **12**, e1005877 (2016).
