# Supplementary material for: The genomic impact of population connectivity and decline in Africa’s elephants
Source: Nat Commun. 2026 Apr 16;17:3223. doi: 10.1038/s41467-026-71262-w (PMC13086863; doi:10.1038/s41467-026-71262-w)
Supplement: Supplementary file 2 — Description of Additional Supplementary Files [file 41467_2026_71262_MOESM2_ESM.pdf]

Description of Additional Supplementary Files

Supplementary data 1. Information about the 249 samples analyzed in this study.

Supplementary data 2. A summary of the data quality control checks.

Supplementary data 3. Details of settings used in analyses in this study.

Supplementary data 4. Proportion of forest ancestry estimated from F4 ratio test, the Admixture analyses at K=2 and from PC1.

Supplementary data 5. Results of the recent parental admixture analysis in apoh.

Supplementary data 6. Genome-wide heterozygosity estimates including and excluding ROH.

Supplementary data 7. Inference of runs of homozygosity in PLINK.

Supplementary data 8. Genetic load estimated in SnpEff.

Supplementary data 9. Sequencing setup.

Supplementary data 10. Details on accession codes for the sequence data generated in this study.
