# Supplementary material for: The genomic impact of population connectivity and decline in Africa’s elephants
Source: Nat Commun. 2026 Apr 16;17:3223. doi: 10.1038/s41467-026-71262-w (PMC13086863; doi:10.1038/s41467-026-71262-w)
Supplement: Supplementary file 4 — Reporting Summary [file 41467_2026_71262_MOESM4_ESM.pdf]

nature portfolio

Corresponding author(s): Patrícia Pečnerová

Last updated by author(s): Jan 20, 2026

Reporting Summary

Nature Portfolio wishes to improve the reproducibility of the work that we publish. This form provides structure for consistency and transparency in reporting. For further information on Nature Portfolio policies, see our Editorial Policies and the Editorial Policy Checklist.

Statistics

For all statistical analyses, confirm that the following items are present in the figure legend, table legend, main text, or Methods section.

n/a	Confirmed	
☐	☒	The exact sample size (*n*) for each experimental group/condition, given as a discrete number and unit of measurement
☐	☒	A statement on whether measurements were taken from distinct samples or whether the same sample was measured repeatedly
☐	☒	The statistical test(s) used AND whether they are one- or two-sided<br>*Only common tests should be described solely by name; describe more complex techniques in the Methods section.*
☒	☐	A description of all covariates tested
☐	☒	A description of any assumptions or corrections, such as tests of normality and adjustment for multiple comparisons
☐	☒	A full description of the statistical parameters including central tendency (e.g. means) or other basic estimates (e.g. regression coefficient) AND variation (e.g. standard deviation) or associated estimates of uncertainty (e.g. confidence intervals)
☐	☒	For null hypothesis testing, the test statistic (e.g. *F*, *t*, *r*) with confidence intervals, effect sizes, degrees of freedom and *P* value noted<br>*Give P values as exact values whenever suitable.*
☐	☒	For Bayesian analysis, information on the choice of priors and Markov chain Monte Carlo settings
☒	☐	For hierarchical and complex designs, identification of the appropriate level for tests and full reporting of outcomes
☐	☒	Estimates of effect sizes (e.g. Cohen's *d*, Pearson's *r*), indicating how they were calculated

Our web collection on statistics for biologists contains articles on many of the points above.

Software and code

Policy information about availability of computer code

Data collection	No code was used for data collection.
Data analysis	Software and code used to analyze the data is described in detail in the Methods section of the manuscript and the scripts used to run analyses will be made available at https://github.com/patriciapecnerova/STAMPEDE. No new code was developed for the purposes of the study. Previously developed pipelines were used for QC and mapping: https://github.com/popgenDK/seqAfrica_bushpigs, and genetic load analysis: https://github.com/ndussex/Crater_lion_genomics/tree/main. In brief, the following tools were used: FastQC v0.11.9; MultiQC v1.13; PALEOMIX BAM pipeline (branch 'pub/2022/africa'); AdapterRemoval v2.3.1; BWA-mem v0.7.17; SAMtools v1.13; BCFtools v1.17; RepeatModeler v2.0.1; RepeatMasker v4.1.0; GEM v20130406-045632; Angsd v0.925; PCAngsd v0.99; NGSadmix (2020.04.20); ngsRelate v2; BEAGLE v3.3.2; winsfs v0.7.0; ADMIXTURE v1.3.0; evalAdmix v0,962; EEMS v0.0.0.9000; ADMIXTOOLS2; PSMC v0.6.5; PLINK v1.9; popSizeABC (2016.10.20); TreeMix v1.13; apoh (commit 3f6491fa55d9820f558368ecfc97eac4e1277a0f); ngsRemix v1.1.0; SnpEff v5.2f; SnpSift v5.2f; Rstudio v2024.12.1.563, R v4.5.1.

For manuscripts utilizing custom algorithms or software that are central to the research but not yet described in published literature, software must be made available to editors and reviewers. We strongly encourage code deposition in a community repository (e.g. GitHub). See the Nature Portfolio guidelines for submitting code & software for further information.

Data

Policy information about availability of data

All manuscripts must include a data availability statement. This statement should provide the following information, where applicable:

- Accession codes, unique identifiers, or web links for publicly available datasets
- A description of any restrictions on data availability
- For clinical datasets or third party data, please ensure that the statement adheres to our policy

The new sequence data is available from the European Nucleotide Archive under accession code PRJEB77639. The study also used published data available from ENA repositories: PRJNA761769, PRJEB24361, PRJNA622303, and PRJEB59491. The data was mapped to the following reference genomes: Asian elephant mEleMax1 available from NCBI project PRJNA850184, mitogenome available from GenBank ID NC_000934.1, and African savanna elephant Loxafr4 available from ftp://ftp.broadinstitute.org/pub/assemblies/mammals/elephant/loxAfr4/

Research involving human participants, their data, or biological material

Policy information about studies with human participants or human data. See also policy information about sex, gender (identity/presentation), and sexual orientation and race, ethnicity and racism.

Reporting on sex and gender	n/a
Reporting on race, ethnicity, or other socially relevant groupings	n/a
Population characteristics	n/a
Recruitment	n/a
Ethics oversight	n/a

Note that full information on the approval of the study protocol must also be provided in the manuscript.

Field-specific reporting

Please select the one below that is the best fit for your research. If you are not sure, read the appropriate sections before making your selection.

☐ Life sciences ☐ Behavioural & social sciences ☒ Ecological, evolutionary & environmental sciences

For a reference copy of the document with all sections, see nature.com/documents/nr-reporting-summary-flat.pdf

Ecological, evolutionary & environmental sciences study design

All studies must disclose on these points even when the disclosure is negative.

Study description	The study aims to describe the contemporary genomic diversity of African elephants by generating high-quality genomes from broad geographic representation of both species.
Research sample	Genomes were generated primarily from collections of blood and tissue samples of African savanna and forest elephants, in total for 226 individuals. Together with published data, 232 samples passed our QC and were used in analyses. These consisted of 181 African savanna elephants and 51 African forest elephants in a total of 29 locations in 17 African countries.
Sampling strategy	Samples were selected from the collections at the University of Copenhagen and University of Illinois based on quality with the aim of covering the broad geographic range of the species, while also targeting at least one location per region with higher number of samples (10+) to improve the power of allele-frequency based analyses.
Data collection	Genomes in this study represent biobanked samples that were collected for previous studies mainly in 1990s and early 2000s. Majority of samples was collected using biopsy darting, some samples were obtained from blood, dry skin, scrapings from carcass remains, and dung. The samples were collected in a way to minimize relatedness from different family groups and solitary males. The samples were imported in compliance with CITES permits, and were collected before the Nagoya protocol came to force.
Timing and spatial scale	Information on sampling dates and locations in available in Supplementary Table 1.
Data exclusions	17 samples were excluded from the analyses due to low quality - ow mapping rate, low coverage, high error rate, high GC content, relatedness, and outlier levels of heterozygosity.
Reproducibility	No experiments were performed as this is an observation study.

Randomization	No experiments were performed as this is an observation study.
Blinding	No experiments were performed as this is an observation study.

Did the study involve field work? ☐ Yes ☒ No

Reporting for specific materials, systems and methods

We require information from authors about some types of materials, experimental systems and methods used in many studies. Here, indicate whether each material, system or method listed is relevant to your study. If you are not sure if a list item applies to your research, read the appropriate section before selecting a response.

Materials & experimental systems

n/a	Involved in the study	
☒	☐	Antibodies
☒	☐	Eukaryotic cell lines
☒	☐	Palaeontology and archaeology
☐	☒	Animals and other organisms
☒	☐	Clinical data
☒	☐	Dual use research of concern
☒	☐	Plants

Methods

n/a	Involved in the study	
☒	☐	ChIP-seq
☒	☐	Flow cytometry
☒	☐	MRI-based neuroimaging

Animals and other research organisms

Policy information about studies involving animals; ARRIVE guidelines recommended for reporting animal research, and Sex and Gender in Research

Laboratory animals	n/a
Wild animals	Samples in this study originate from biobanked scientific collections, and no field work was carried out on wild analyses specifically for the purposes of this study.
Reporting on sex	Where the information was available, we report the sex of the animals in Supplementary Table 1. Sex identification wasn't performed specifically for the purposes of this study.
Field-collected samples	The study did not involve samples collected in the field for this study.
Ethics oversight	Samples were collected complying with local and international legislation. All sample collection predates the Nagoya protocol. The research was carried out in compliance with the Code of Conduct for Responsible Research of the University of Copenhagen and the University of Illinois Institutional Animal Care and Use Committee approved protocol number 24029. The tissue samples were imported to Copenhagen, Denmark under CITES import permit IM 1208-989/05.

Note that full information on the approval of the study protocol must also be provided in the manuscript.

Plants

Seed stocks	n/a
Novel plant genotypes	n/a
Authentication	n/a
